# A heuristic underlies the search for relief in *Drosophila melanogaster*

**DOI:** 10.1111/nyas.14730

**Published:** 2021-12-20

**Authors:** Nicola Meda, Giulio Maria Menti, Aram Megighian, Mauro Agostino Zordan

**Affiliations:** ^1^ Department of Biomedical Sciences University of Padova Padova Italy; ^2^ Padova Neuroscience Center University of Padova Padova Italy; ^3^ Department of Biology University of Padova Padova Italy

**Keywords:** nearest neighbor rule, local search, visual learning, navigation, hierarchical behavior, decision making

## Abstract

Humans rely on multiple types of sensory information to make decisions, and strategies that shorten decision‐making time by taking into account fewer but essential elements of information are preferred to strategies that require complex analyses. Such shortcuts to decision making are known as heuristics. The identification of heuristic principles in species phylogenetically distant to humans would shed light on the evolutionary origin of speed–accuracy trade‐offs and offer the possibility for investigating the brain representations of such trade‐offs, urgency and uncertainty. By performing experiments on spatial learning in the invertebrate *Drosophila melanogaster*, we show that the fly's search strategies conform to a spatial heuristic—the nearest neighbor rule—to avoid bitter taste (a negative stimulation). That is, *Drosophila* visits a salient location closest to its current position to stop the negative stimulation; only if this strategy proves unsuccessful does the fly use other learned associations to avoid bitter taste. Characterizing a heuristic in *D. melanogaster* supports the view that invertebrates can, when making choices, operate on economic principles, as well as the conclusion that heuristic decision making dates to at least 600 million years ago.

## Graphical abstract

By performing experiments on spatial learning in the invertebrate *Drosophila melanogaster*, we show that the fly's search strategies conform to a spatial heuristic—the nearest neighbor rule—to avoid bitter taste (a negative stimulation). Characterizing a heuristic in *D. melanogaster* supports the view that invertebrates can, when making choices, operate on economic principles, as well as the conclusion that heuristic decision making dates to at least 600 million years ago.

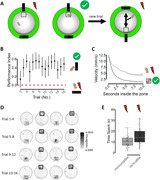

## Introduction

Navigating an environment is a complex process, and several organisms across different taxa have been studied to address how animals apply diverse strategies to move in their surrounding environments.^1^, ^2^ The picture that emerges from these studies is that different animals can apply analogous behaviors during similar navigation demands to reach their targets,^3^, ^4^, ^5^ such as food, shelter, or peers. To achieve their goals, animals frequently have to make adequate decisions by leveraging different sources of information.^6^, ^7^ However, considering multiple cues before making a choice is an effortful strategy that may hinder the chances of survival.^8^ Instead of a piecemeal, in‐depth analysis of sensory information, humans appear to privilege strategies that minimize cognitive load (i.e., use the least amount of information) and shorten decision‐making time, but still lead to appropriate and adaptive behavior.^9^, ^10^ Such strategies are heuristics.^11^ For example, option discrimination can rely on a heuristic termed *take the best*; according to this heuristic, only the most reliable cue in relation to discriminatory capacity between different outcomes^12^ (e.g., punishment or reward) is used for inference, while cues with lower predictive value are not taken into consideration.^13^, ^14^ Several suboptimal choices made by nonhuman animals could be deemed to be based on heuristic principles, yet such behaviors are rarely adequately acknowledged.^15^

Given that heuristics are shortcuts to decisions, animals are expected to implement these strategies under uncertainty,^16^ during highly complex tasks,^17^ or in urgency. Only later, environmental conditions allowing, or if the goal has not been achieved, animals may adjust their decisions accordingly.^18^ Moreover, diverse spatial heuristics (strategies applied specifically during navigation) have been described in humans, thus a comparative search for the same strategies in other animals could be relatively straightforward.^17^, ^19^ One of these heuristics is the nearest neighbor rule (NNR), also known as the proximity rule. According to the NNR, a moving animal searching for food (or other relevant stimuli) visits the closest location to its current position first, then travels to the next closest position, and so on.^20^ Most of the research on the subject has been conducted in the field of behavioral economics^21^ (for a historical reference, see Ref. 22).

There is a knowledge gap in the origin of heuristic principles and their conservation through evolutionary time, whereas this information could be a fruitful entry point to investigate uncertainty and urgency in nonhuman animals. This lag is understandable considering the complexity of adapting tasks specifically for humans to study in other animals. We hypothesized that short‐range navigation tasks (i.e., those conducted in a laboratory) satisfy the criteria for investigating heuristic principles from a comparative standpoint: animals can be forced to choose under uncertainty by controlling the explorable environment^23^ and stimulating them negatively, or by designing more complex tasks.^24^

Identifying heuristic principles in phylogenetically distant species would shed light on the origin of speed–accuracy trade‐offs.^25^, ^26^ The recognition of use of the same/similar strategic principles in model organisms would provide an entry point for investigating brain representations associated with such trade‐offs and cognitive load, urgency, and uncertainty, and might also provide insight into the use of heuristic techniques for solving scheduling tasks in the field of artificial intelligence.

In our study here on spatial learning, we show that *Drosophila melanogaster* are able to associate relief from bitter taste (a negative stimulation that is innately associated with a threat of intoxication) to a specific visual landmark and then search for an outcome (i.e., relief) in the proximity of the landmark. Specifically, we observed that in order to relieve an unpleasant stimulation, *Drosophila* first search near their current position and then, shortly after, approach an appropriate landmark. We conducted a set of experiments aimed at investigating whether this behavior was the result of insufficient learning or was a trial‐and‐error strategy. The data suggest that initial search behavior of fruit flies can be explained by the application of the NNR—a heuristic strategy in line with what has been shown for pigeons,^27^ primates,^25^ rats, and humans^28^—whereas learned visual information is used only if the NNR is unsuccessful.

## Results

**Figure 1:**
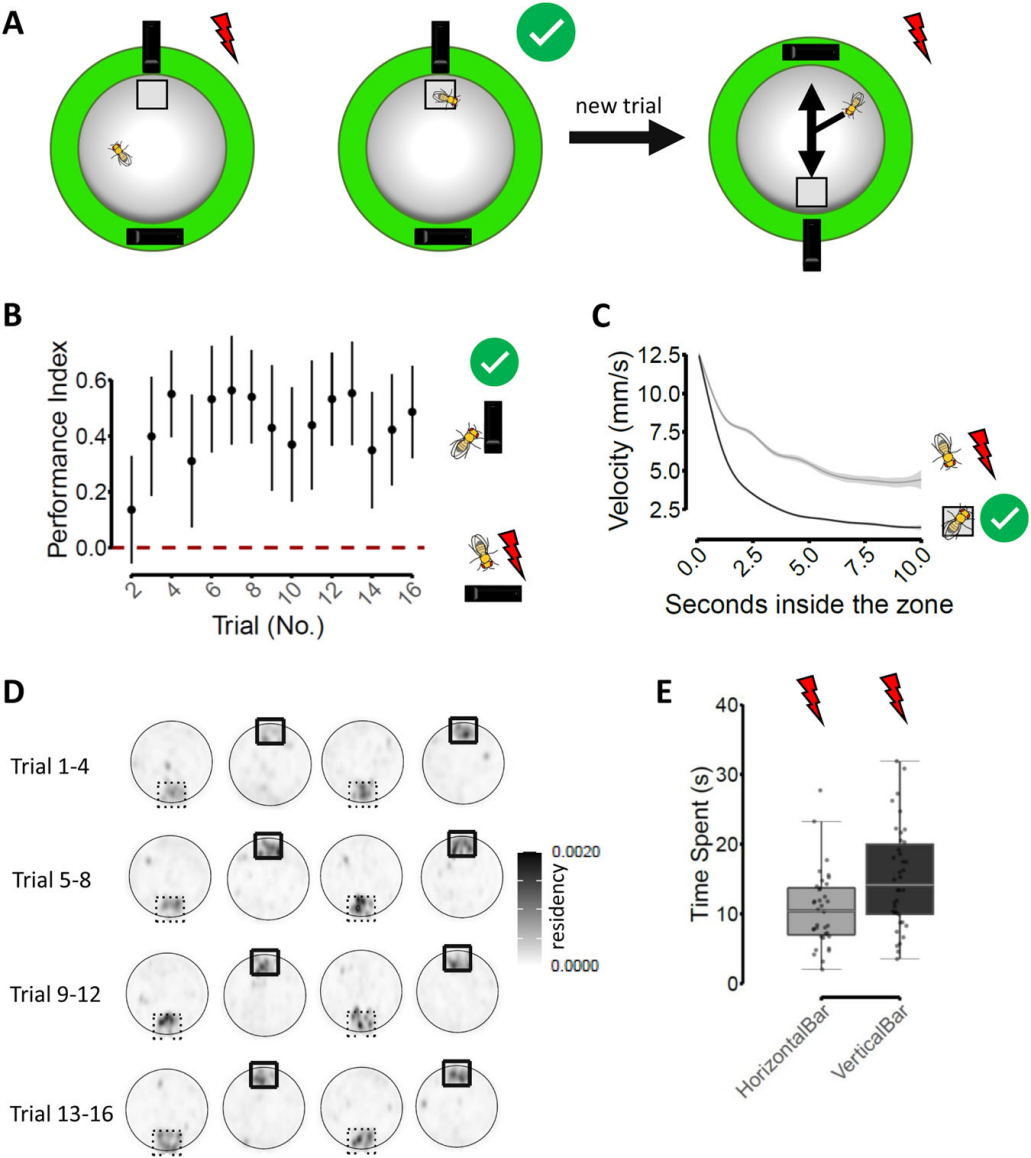
Behavioral paradigm and fruit flies learning. Black bars represent the vertical or horizontal stripe; the gray square represents the safe zone; the red bolt represents that the fly is being negatively stimulated; and tick on green background represents that the fly is safe from the negative stimulation. (A) Summary of the behavioral paradigm and the experimental question. (B) Performance index (PI) as a function of training trials. PI is the difference between the time spent in the safe zone and the time spent in the previous (location of the) safe zone, divided by the total time spent in the two zones. PI can range from 1 to –1. A value of zero indicates no zone preference. PI was not computed for trial 1, given the absence of a previous safe zone during that trial (see also Table S3, online only). Point range is the mean ± confidence interval around the mean. (C) Velocity profile of fruit flies after entering a zone, where relief is provided (black line is fly on square with tick on green background) compared with the profile (gray line) after entering the nonsafe zone (fly with red bolt; see Table S4, online only). (D) Density plots describing the residency of flies during the training session, when bitter‐taste stimulation is triggered if flies leave the safe zone (squared). (E) During the probe session, the vertical bar was located either at the northern, southern, eastern, or western end of the arena (*n* = 10 flies were tested for each location; no. of observations (flies * zone) = 75; vertical bar – horizontal bar mean difference in time spent (s) 5.03, SE = 1.47, *z* ratio = 3.14, *P* = 0.0017).

*D. melanogaster* can be trained to distinguish identical objects with different orientations.^29^, ^30^, ^31^ A blinded experimenter used a circular arena to train single flies (*n* = 40) to differentiate between a black vertical stripe, which marked the presence of a “safe zone” (6 cm^2^ surface) linked to relief from optogenetically induced bitter taste, from a diametrically opposed horizontal stripe, which was not associated with relief. The training session was composed of 16 trials (i.e., repetitions), each 3 min long. During the first 30 s of each trial, the fly was free to explore the arena in complete darkness; for the next 30 s, the fly could explore the arena in the presence of the diametrically opposed visual patterns (black horizontal/vertical bar on a homogenously lit background). In the last 2 min, the fly could experience bitter taste according to its position in the arena, while still in the presence of the visual patterns. At the beginning of each new trial, the positions of the matched vertical stripe safe zone and horizontal stripe were switched (Fig. 1A; details regarding Materials and Methods are in Supplementary Materials, online only; this file also includes data Figs. S1–S5 and Tables S1–S8, online only). We considered the time spent in the safe zone throughout the training session (Fig. 1B and Table S3, online only) and the preference for the vertical bar (measured in relation to time spent in its proximity) during the probe session (Fig. 1E) as a proxy for learning (see Figs. S1 and S2, Tables S1 and S2 for control experiments, online only). At the start of each new trial, given the switch of the horizontal bar and vertical bar‐safe zone positions, the flies could experience two potentially conflicting sources of information as to the current spatial location of the safe zone: it could be located either in the spatial location, where relief was last experienced during the preceding trial (where the horizontal stripe was currently displayed), or in the proximity of the vertical bar. In this cue–conflict paradigm, the flies could use either a local search strategy centered on the spatial location most recently associated with relief or the learned visual association between the vertical stripe and relief to approach the safe zone. Analysis of local search strategies was conducted by considering only the trials during which each fly was not already inside one of the two areas of interest, that is, the current safe zone (CSZ) or the previous safe zone (PSZ), at the onset of stimulation (the flies already inside the CSZ would not experience any negative stimulation; the first zone approached by the flies already inside the PSZ would clearly be the PSZ itself).

**Figure 2:**
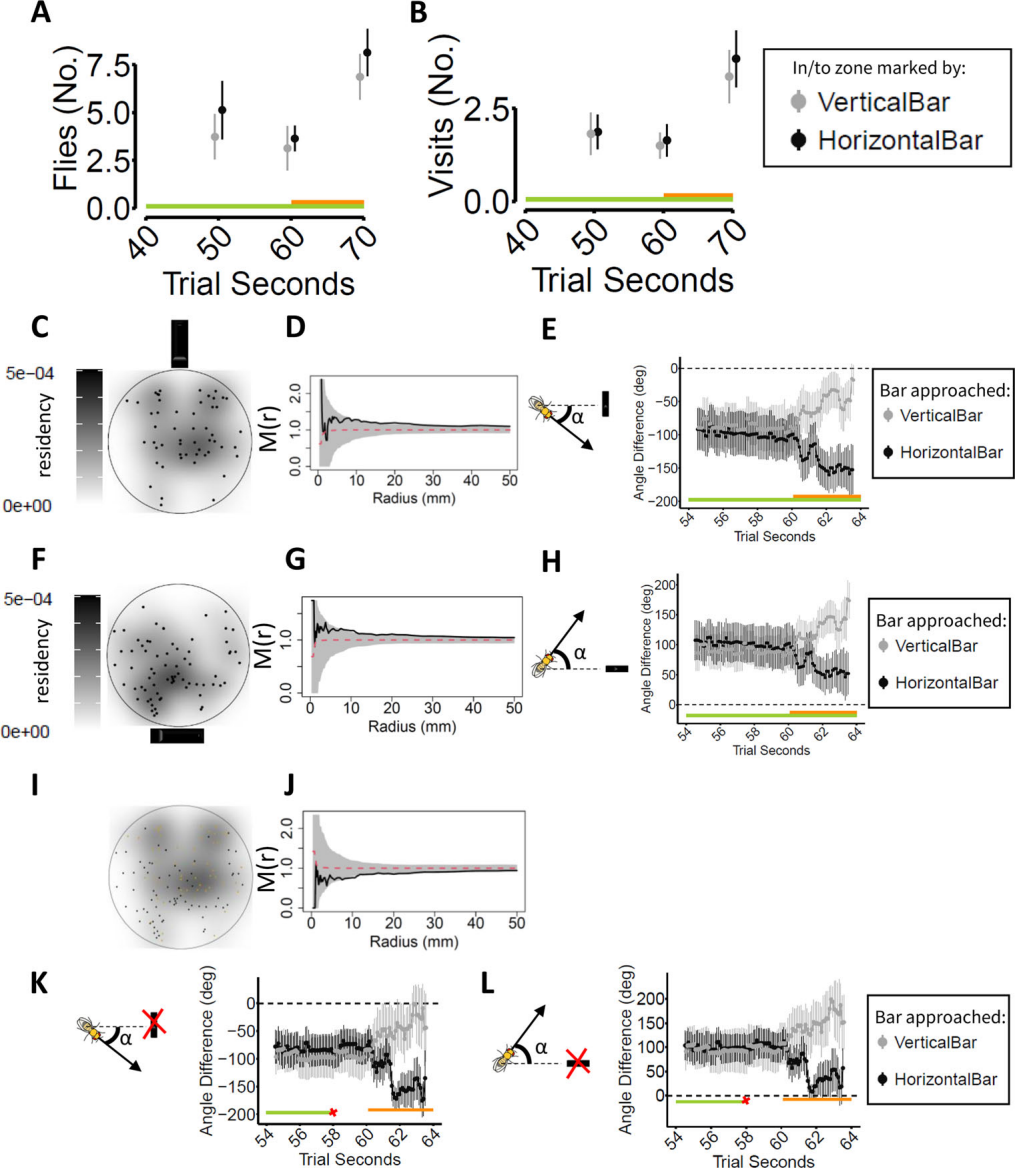
*Drosophila* follow the NNR soon after bitter stimulation onset. (A) In the 10 s after the onset of optogenetic stimulation, the difference between the number of flies that approached either one of the two zones is not significant (Table S4, online only). (B) There is no difference between the mean number of visits to the two zones in the periods considered (Table S4, online only). (C) Spatial position of flies, at second 60.1 (when the first pulse of optogenetic stimulation is delivered) that first entered the safe zone, marked by the vertical stripe. (D) Marcon and Puech's M function value (black line) represents the distance between the observed positions of flies compared with 10,000 random distribution simulations (red dashed line and gray shading). A value greater than 1 suggests aggregation. (E) Flies that entered the safe zone oriented themselves toward that zone after the onset of the bitter stimulation. (F) Spatial distribution at second 60.1 of flies that will approach the previous safe zone. (G) M function value is significantly greater than expected under the null hypothesis, suggesting aggregation of flies. (H) The flies that entered the previous safe zone oriented themselves toward it (see also Table S4, online only). (I) Spatial position of both groups of flies at second 60.1. (J) M function value assessing whether the two distributions of flies’ positions reported in C, F, or I consist of two distinct aggregates. The M function is <1, thus suggesting spatial repulsion between the two groups of flies. (K) Flies reorient themselves toward the expected location closest to the landmark (in this case, the vertical stripe, even though the landmark is occluded from vision: black bar with a red cross on top). (L) Same as K; in this case, the horizontal stripe would be the closest landmark that is occluded from vision. Even‐numbered trials. Green bar represents visual patterns displayed; orange bar represents stimulation triggered according to fly position. Point range is mean ± confidence interval around the mean.

We found that flies significantly increased the number of visits to both zones (PSZ and CSZ) during the first 10 s of optogenetic stimulation (Fig. 2A and Table S4, online only; number of observed visits = 151, model estimate mean [log‐scale] = 0.883, SE = 0.15, *z* ratio = 5.61, *P* < 0.0001) with no preference for either of the two zones. This increase is also reflected by the higher mean number of flies that can be found in both zones during this period of time, with respect to what was observed 10 s before the onset of stimulation (Fig. 2B and Table S4, online only; number of observed flies = 89, model estimate mean difference = 0.80, SE = 0.12, *z* ratio = 6.619, *P* < 0.0001). The fact that the PSZ and CSZ were visited in the same measure by the same number of flies suggests that flies did not take into consideration which visual marker they were approaching (although they learn to differentiate between two identical shapes with different orientations, see Fig. 1 and Refs. 29, 30, 31). Thus, we hypothesized that the animals first tend to direct themselves to the zone closest to their current position (a rapid behavior) and only later take into consideration the orientation of the visual marker (a slower but more accurate response).^14^, ^32^

In order to evaluate whether this was the case, we proceeded to identify the positions of flies at the onset of optogenetic stimulation and separated the animals into two groups according to which was the first zone entered. Figure 2C and F shows the positions of flies that entered the CSZ or PSZ (marked by the horizontal bar), respectively, during the trials in which the CSZ was located at the north end of the arena (for ease of readability, data on trials in which the CSZ was located at the south end are presented in Fig. S3, online only). These density plots suggest that flies tended to enter the zone closest to them at the onset of the optogenetic stimulation, a behavior consistent with the use of the NNR.

Next, we tested whether the positions of each group of flies for each set of trials (i.e., subdivided according to whether the CSZ was at the northern or southern end of the arena) were more aggregated than expected under the null hypothesis of a random distribution. To do this, we applied Marcon and Puech's M function^33^, ^34^, ^35^ and tested if the observed positions of the flies were more aggregated than expected based on the generation of 10,000 simulated random positions. For both sets of trials, and for both groups of flies (i.e., the flies grouped according to which zone was entered first), animals showed evidence for significantly greater aggregation than expected (see Fig. 2D, goodness‐of‐fit test testing for aggregation *P* = 0.012 (meaning M(r) > 1), for *n* = 63 positions tested; Fig. 2G, goodness‐of‐fit test *P* = 0.030, for *n* = 62 positions; and Fig. S3, online only). Moreover, if the position of a fly in the arena was a reasonably good predictor of the first zone visited, the position of flies predicted to enter the PSZ should be spatially segregated from the position of flies predicted to enter the CSZ. In fact, we found that the positions of the two groups of flies show significant segregation in both sets of trials (see Fig. 2J, goodness‐of‐fit test testing for spatial repulsion *P* = 0.031 (meaning M(r) < 1) for *n* = 125 positions; and Fig. S3, online only).

If the observed behavior of the flies is truly the result of the application of an NNR model, furthermore, the flies would not enter a given zone by chance, that is, behavior better explained by random navigation.^19^ In other words, by the NNR model, the flies should first orient themselves toward the closest zone, irrespective of the visual marker associated with it, and then travel toward it. Our data showed that, shortly after the beginning of the optogenetic stimulation, flies reorient themselves toward the closest zone before approaching it (for flies approaching the vertical bar, see Fig. 2E, number of orientations tested = 8937, mean orientation difference after‐before the onset of stimulation in degrees = 25.5, SE = 2.35, *z* ratio = 10.84, *P* < 0.0001; for flies approaching the horizontal bar, see Fig. 2H mean orientation difference = 36.2, SE = 2.14, *z* ratio = 16.86, *P* < 0.0001; and Fig. S3, online only). Moreover, by the NNR model, the target toward which a fly orients itself should depend on the position of the fly in the arena. If this is, indeed, the case, the orientation of the flies at the onset of stimulation (second 60.1), after controlling for position, would not be a good predictor of the zone, which will then be approached.

To test this, we modeled the zone that each fly would eventually enter on the basis of its position alone or on both position and orientation (Table S4, online only). We found that the fly position in the arena was necessary and sufficient to predict which zone it would enter first; the addition of orientation did not provide a better explanation of the observed data.

Last, we tested flies (*n* = 30) under a different paradigm: during each trial, 2 s before the onset of optogenetic stimulation and 4 after the onset, the flies were presented solely with a homogeneously lit background, without landmarks (in this situation, the stripes were present until second 58 of the trial and reappeared after second 64). We ascertained that flies reoriented themselves toward the supposed location of the landmark and traveled toward the expected position, although no stripe was present (see Fig. 2K, number of orientations tested = 6917, mean orientation difference after‐before the onset of stimulation in degrees = 44.3, SE = 3.09, *z* ratio = 14.36, *P* < 0.0001; Fig. 2L, mean orientation difference = 37.2, SE = 2.4, *z* ratio = 15.45, *P* < 0.0001; and Fig. S3, online only, for odd‐numbered trials). As previously reported, this behavior can be explained by considering that the fly is using the memory of the landmark position in the environment to guide its goal‐directed navigation.^36^ We conducted a replicate set of the experiments with a new group of flies (*n* = 41; see Fig. S4, Tables S5 and S6, online only). We also conducted an additional set of experiments with 40 more flies in which they faced a visual environment with two identical and diametrically opposed vertical bars. We alternated the position of the safe zone between the two bars on different trials. The results suggested that the initial search strategy (again, resorting to the NNR) used by the flies appeared to be independent of the width or orientation of the visual cues (see Fig. S5, Tables S7 and S8, online only). These experiments further confirmed that the zone first entered by a fly can be predicted by its spatial location at the onset of the negative stimulation, consistent with behavior following the spatial heuristic known as the NNR.^19^, ^24^, ^37^, ^38^

## Discussion

In order to survive, animals have to evaluate environmental and internal information and make decisions rapidly. However, rapidity comes with imprecision, and the trade‐off between speed and accuracy differentiates between a perfect but inapplicable strategy, a fast and frugal strategy, and an immediate but unsuccessful approach.^8^, ^14^, ^32^ Heuristics are shortcuts extensively used by humans and characterized by an optimal balance between speed and accuracy.^38^, ^39^, ^40^ The use of these shortcuts can be elicited by pressing the animal with urgency, uncertainty, or cognitive load (e.g., by forcing it to accomplish two tasks simultaneously).^14^, ^17^ In contrast to the analysis of heuristics in human cognitive psychology and behavioral economics, the investigation of these strategies in behavioral neuroscience has lagged behind, possibly because of the inapplicability of the economic paradigms in animals other than humans, but probably also because heuristics, when unacknowledged, are regarded as behavioral “noise” or as being due to insufficient learning.^15^ During experiments on spatial learning, conceptually similar to the paradigms described in Refs. 23 and 37, we showed that fruit flies learned the spatial association between a vertical bar and relief from an unpleasant stimulation produced via optogenetic stimulation of bitter‐sensing neurons. However, we also acknowledged that the search behavior of *D. melanogaster* at the onset of the stimulation resembled a spatial heuristic. We fortuitously elicited this spatial heuristic probably because we exposed the flies to bitter taste, which is a negative but ecologically relevant stimulation (thus providing urgency) that, nonetheless, can be leveraged in a learning paradigm.^41^ Moreover, under these conditions, flies faced conflicting cues regarding where the relief from the stimulation could take place (uncertainty). Urgency and uncertainty are two circumstances that trigger the emergence of heuristic behavior.^9^, ^39^ With this paradigm, we observed that fruit flies initially adopt the heuristic known as the NNR to escape punishment, and only if this proves unsuccessful, they resort to other strategies. According to the NNR, a moving animal seeking food or ecologically relevant information should visit the closest location to its current position first, and then travel to the next closest position, and so on.^20^ In our experiments, the fruit flies approached the closest visual marker to their current position, or the closest position, where they expect the landmark to be (even in the absence of a landmark, Fig. 2K and L).^36^ Only if this goal‐oriented navigation proved unsuccessful, the animals resorted to the learned visual information in order to locate the safe zone. This heuristic was already described in mammals,^38^ as well as other vertebrates,^24^ but the evidence herein supports the idea^26^ that insects also employ “shortcuts” to decision making, and that from an evolutionary point of view, the existence of heuristics, in particular of the NNR, might date to the last common ancestor of arthropods and vertebrates.^42^

## Data and materials availability

Datasets are available at 10.17632/9frwpy5vz9.1; for customized MatLab script, see Ref. 43.

## Author contributions

Conceptualization and methodology: N.M., G.M.M., A.M., and M.A.Z.; software: M.A.Z.; investigation: N.M. and G.M.M.; formal analysis, visualization, and writing of original draft: N.M.; funding acquisition and upervision: A.M. and M.A.Z.; writing, review, and editing: G.M.M., A.M., and M.A.Z.

## Competing interests

The authors declare no competing interests.

### Peer review

The peer review history for this article is available at https://publons.com/publon/10.1111/nyas.14730.

## Supporting information


**Supplementary Figure S1**. Flies reared on food without retinal are not affected by red light stimulation.
**Table S1**. Related to Suppl. Figure S1B. Performance index, for each trial, for flies reared on food without retinal.
**Supplementary Figure S2**. Gr66a‐Gal4 > CsChrimson flies that cannot achieve relief do not “naturally” search in proximity to a specific landmark.
**Table S2**. Related to Suppl. Figure S2B. Performance index for each trial.
**Table S3**. Related to (main text) Figure 1B. Performance index for each trial.
**Supplementary Figure S3**. Fruit flies resort to the nearest neighbor rule soon after bitter stimulation onset. Odd‐numbered trials.
**Table S4**. Models details related to Figures 1 and 2, and Suppl. Figure S3.
**Supplementary Figure S4, part 1**. Fruit fly training–replication set
**Supplementary Figure S4, part 2**. Fruit flies resort to the nearest neighbor rule soon after bitter stimulation onset–replication set
**Table S5**. Related to Suppl. Figure S4. Performance index for each trial.
**Table S6**. Models details related to the replication set described in Suppl. Figure 4.
**Supplementary Figure S5, part 1**. The nearest neighbor rule is applied even in an ambiguous visual environment.
**Supplementary Figure S5, part 2**. The nearest neighbor rule is applied even in an ambiguous visual environment.
**Table S7**. Related to Figure S5B. Performance index of flies trained in an ambiguous visual environment.
**Table S8**. Models details related to Suppl. Figure S5.
