# Supplementary material for: A heuristic underlies the search for relief in Drosophila melanogaster
Source: Ann N Y Acad Sci. 2021 Dec 20;1510(1):158–66. doi: 10.1111/nyas.14730 (PMC9300192; doi:10.1111/nyas.14730)
Supplement: Supplementary file 1 — Supplementary Figure S1. Flies reared on food without retinal are not affected by red light stimulation. Table S1. Related to Suppl. Figure S1B. Performance index, for each trial, for flies reared on food without retinal. Supplementary Figure S2. Gr66a‐Gal4 > CsChrimson flies that cannot achieve relief do not “naturally” search in proximity to a specific landmark. Table S2. Related to Suppl. Figure S2B. Performance index for each trial. Table S3. Related to (main text) Figure 1B. Performance index for each trial. Supplementary Figure S3. Fruit flies resort to the nearest neighbor rule soon after bitter stimulation onset. Odd‐numbered trials. Table S4. Models details related to Figures 1 and 2, and Suppl. Figure S3. Supplementary Figure S4, part 1. Fruit fly training–replication set Supplementary Figure S4, part 2. Fruit flies resort to the nearest neighbor rule soon after bitter stimulation onset–replication set Table S5. Related to Suppl. Figure S4. Performance index for each trial. Table S6. Models details related to the replication set described in Suppl. Figure 4. Supplementary Figure S5, part 1. The nearest neighbor rule is applied even in an ambiguous visual environment. Supplementary Figure S5, part 2. The nearest neighbor rule is applied even in an ambiguous visual environment. Table S7. Related to Figure S5B. Performance index of flies trained in an ambiguous visual environment. Table S8. Models details related to Suppl. Figure S5. [file NYAS-1510-158-s001.docx]

Supplementary Materials

A spatial heuristic underlies the search for relief in fruit flies

Nicola Meda, Giulio M. Menti, Aram Megighian, Mauro A. Zordan

Correspondence to: [aram.megighian@](mailto:aram.megighian@)unipd.it

**This File includes**

Supplementary Methods…..…………...…...………………….………………………...……………….……… 2

Supplementary Figure S1.…………........………………………...……....…………………………………. ....11

Supplementary Table S1..…………………………………………...…………………...….……………….… 12

Supplementary Figure S2.…………………………………………...……………………………………….… 13

Supplementary Table S2…………………………………………...…………..…………………………….… 14

Supplementary Table S3…………………………………………...……………..………………………….… 15

Supplementary Figure S3..………………………………………...………………...………………………..... 16

Supplementary Table S4..………………………………………………...………………………………….… 18

Supplementary Figure S4..………………………………………………...………….…………………….….. 21

Supplementary Table S5..………………………………………………...……………………………………. 25

Supplementary Table S6..………………………………………………...……………………………………. 26

Supplementary Figure S5..………………………………………...…………………….……………….....….. 29

Supplementary Table S7..……………………………………………….………………………………….….. 32

Supplementary Table S8..………………………………………………...……………..………………….….. 33

**Supplementary Methods**

The procedures described herein had also been implemented in Ref. 43.

**Experimental Models**

We obtained the experimental progeny from crossing Blooming Stock Center (BSC) line 57670 (genotype: *w*[*]; *wg*[Sp-1]/*CyO*; P{w[+mC]=*Gr66a*-*GAL4*.1.8}1/*TM3*, *Sb*[1]), characterised by bitter-sensing-neuron-restricted expression of Gal4, to a UAS-CsChrimson construct-bearing line (BSC line 55135; genotype: *w*[1118]; P{y[+t7.7] w[+mC]=*20XUAS-IVS-CsChrimson.mVenus*}attP40). The aforementioned Rhodopsin requires all*-trans-*retinal to be operated by red-shifted light (625nm) ^44,45^. For our behavioural experiments we opted to use only adult male flies. Offspring selected for experiments were *CyO*-negative.

Parental lines were reared in plastic vials (height 13cm, diameter 2,5cm) containing 10ml of standard cornmeal medium supplemented with 0.2mM of all-trans-retinal (Sigma-Aldrich, CAS: 116-31-4). Parental lines (10 males from 57670 line and 5 virgin females for each cross – selected 1-2 days after birth) were kept in the same vial to mate for 4-5 days. After 4-5 days of mating, the parental flies were moved to another clean vial to mate and lay more eggs, and this procedure was repeated 3-4 times.

Offspring birth was monitored every day (between 9.00 am and 11.00 am), and hatched adult individuals were selected according to phenotypic markers. 8-10 adult male individuals of such selected offspring were put in the same vial. The vial for rearing offspring was filled with 5ml of standard cornmeal medium supplied with 0.4mM all*-trans-*retinal. The vials were then put in a black TJENA box (Ikea, SW). Flies were kept in complete darkness inside a black box until the day of the experiment to avoid all*-trans-*retinal degradation.

**Optogenetics apparatus**

We loaded flies in a transparent resin arena covered with a glass cover (0.3mm thick), both having a diameter of 109 mm. The arena presents a flat inner circular area of 5.5 cm diameter. Within this area, the distance between the bottom of the arena and the cover is 3.5mm; the arena then gently slopes upward towards the edge with an 11° degree angle, thus gradually reducing the distance between the cover and the base. This angle is necessary to avoid the presence of a “walkable rim”. Arena and cover were designed with AutoCAD® 2015, and 3D printed using transparent resin by iMaterialise, BE. The arena was placed on a raised platform; 15 cm underneath this platform, an infrared light-emitting LED lamp (LIU850A, Thorlabs Inc., USA) illuminates the arena lower surface. We placed 3 sheets of tracing paper between the arena and the lamp to diffuse and reduce the infrared light intensity. 36 cm above the arena, a Chameleon 3 camera (CM3-U3-13S2C-CS-BD, FLIR, USA), equipped with an IR bandpass filter, was used to record fly movements. A cylinder composed of 48 panels, each consisting of an 8x8 grid of green (520nm) LEDs (JF-MR-FA0001 Rev B, IO Rodeo, USA), encircled the arena and was used to display visual stimuli. We controlled individual LEDs to display specific lighting patterns through a MATLAB script, uploaded to the LED controller via SD Card. 30 cm above, and on opposing sides of the arena, 3-red-emitting-leds (SR-03-D2050, LuxeonStar, USA) per side were controlled by a programmable Genuino circuit board (UNO REV3, Arduino, IT) and constantly delivered pulses of 0.24mW/cm^2^ red light (measured with a Photodiode Power Sensor, model PM16-120, by Thorlabs, USA) stimulation according to the position of the fly, which was being tracked live.

Each component of this setup was placed inside a chamber covered by a thick black cloth, thus isolating the whole setup from environmental light. In order to deliver time-specific optogenetic stimulation according to the position of the fly in the arena, the animals were tracked live using a modified version of the Motion-Based Multiple Object Tracking script by MathWorks® (<https://it.mathworks.com/help/vision/examples/motion-based-multiple-object-tracking.html>), customized by us to meet our specific requirements. In particular, we used a live tracking system to record fly movements at 11 frames-per-second. The script used real-time fly coordinates to drive the Genuino board's output signals (and consequently the optogenetic stimulation) as follows: whenever the (x; y) coordinates of the centroid representing the fly were within a "safe zone" in the arena, the optogenetic stimulus was turned off; as soon as the fly left the safe zone, the stimulus was turned on.

**Behavioural experiments**

Adult male flies aged 6-10 days, were aspirated singularly from the rearing vial and loaded into the arena. The temperature inside the box was assessed with a PTC-10 thermistor (NPI electronic, DE) and varied between 21-23°C degrees; Humidity inside the box varied between 35-55% (measured with a TH-50 probe by Hama, DE).

After loading the fly, the arena was placed on the raised platform, and the cylindrical LED display lowered around the arena. The lower row of cylinder LEDs was below the level of the arena in order to ensure the homogeneous diffusion of light. The whole apparatus was then covered with black cloth and the customised MATLAB script executed. For the set of experiments described in the main text, the experimenter (V.T., see the acknowledgements) was blinded to the experimental condition being run.

Behavioural experiments consisted of two parts, a training session and a probe session. Our analyses were based solely on the training session, if not otherwise specified. Each training session was made up of 16 trials. For the Nearest Neighbour Rule investigation (e.g., in the main text Figure 2), we did not consider the first trial for our analysis, given that in this case no “Previous Safe Zone” could be defined. For testing flies training/learning, we analysed 16 trials. Each trial lasted 3 minutes: during the first 30 seconds the fly was free to explore the arena in complete darkness; for the next 30 seconds the fly could explore the arena with the green LED lights on. The LEDs were programmed to display two diametrically opposed black stripes of the same area, one vertical and the other horizontal, on an evenly lit background. With respect to the centre of the arena (55.5mm away from the LED cylinder), the vertical stripe azimuth (width) covered 7,83° on either side (left/right); the vertical stripe height covered from 0° to 47°. The horizontal stripe azimuth covered 27,2° on either side (due to the led cylinder's curvature); the horizontal stripe height covered from 22,4° to 34,5°. During the last 2 minutes of a trial, the fly could be subjected to optogenetic stimulation according to its position in the arena, with the green LED visual patterns still present. Whenever the fly’s coordinates were inside a virtual safe area, no optogenetic stimulation took place; as soon as the fly left the safe area, the optogenetic stimulus (red-LEDs) was automatically switched on, and bitter-sensing neurons stimulated. The safe zone was defined to be in close proximity to the vertical stripe, and it covered an area of 6 cm^2^ (the total surface of the arena is 89,87 cm^2^). In each trial, the safe zone-vertical stripe match was rotated by 180°: during odd-numbered trials (1^st^, 3^rd^,…,15^th^), the safe zone was adjacent to the ‘southern end’ stripe; during even-numbered trials, the safe zone was adjacent to the ‘northern end’ stripe.

The probe session is the 17^th^ trial, which is identical to any other of the sixteen trials of the training session, except for the fact that the negative stimulation does not cease when the animal enters the safe zone (for details on the control experiments and further sets of experiments, please refer to the **flow-chart reported on the next page**).

Once the probe session ended, the fly was aspirated from the arena and discarded; the arena was then cleaned with distilled water and dried with blotting paper.


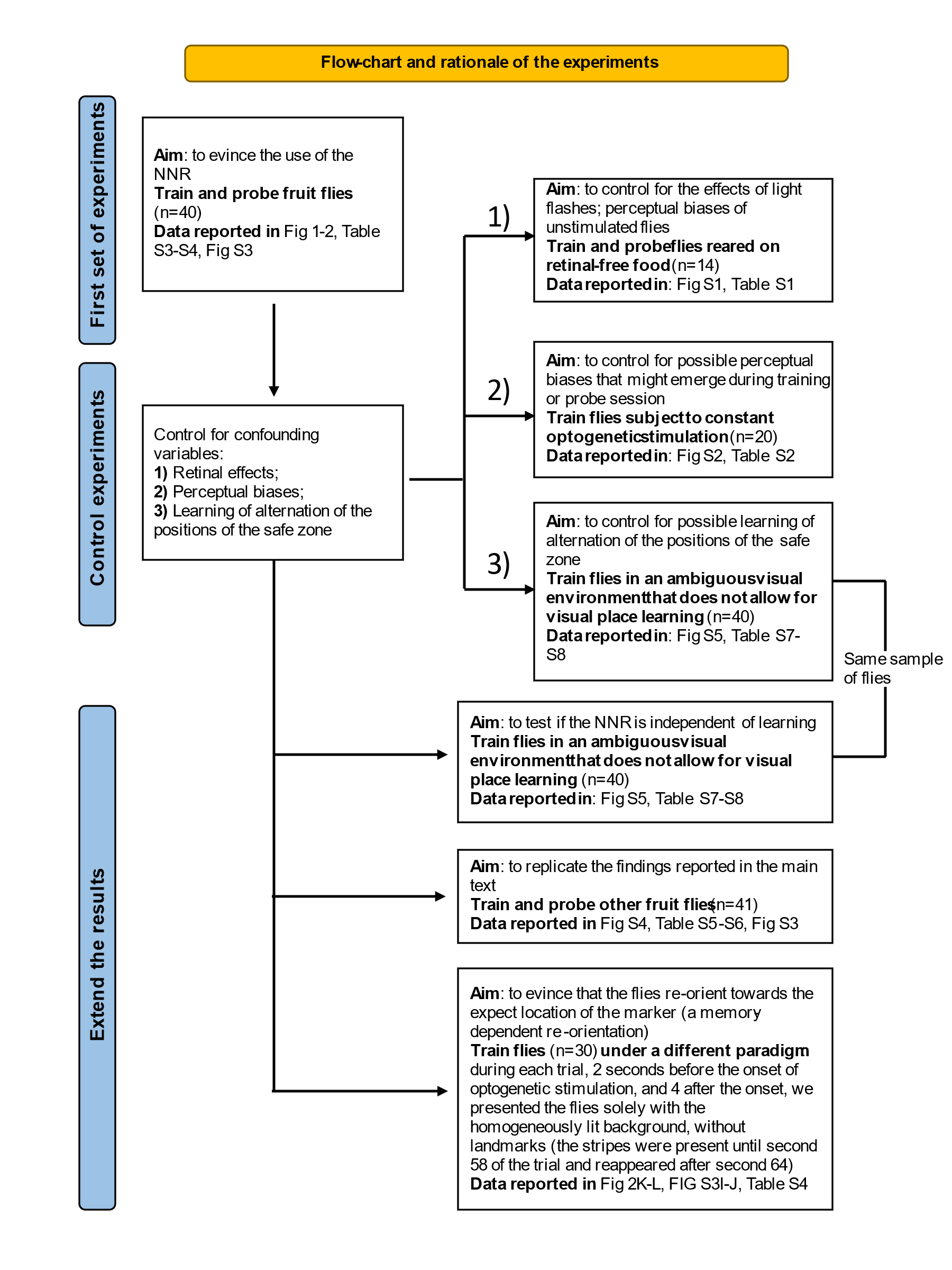


**Regarding the flow-chart above:** in the first set of experiments (see also the main text), we trained the fruit flies (n=40) to differentiate between a black vertical stripe, which marked the presence of a “safe zone” linked to relief from optogenetically-induced bitter taste, from a diametrically-opposed horizontal stripe which was not associated to relief. The training session was composed of 16 trials (i.e., repetitions), each three minutes long. During the first 30 seconds of each trial, the fly was free to explore the arena in complete darkness; for the next 30 seconds, the fly could explore the arena in the presence of the diametrically opposed visual patterns (black horizontal/vertical bar on a homogenously-lit background). In the last 2 minutes, the fly could experience bitter taste according to its position in the arena, while still in the presence of the visual patterns. At the beginning of each new trial, the positions of the matched vertical stripe – safe zone and of the horizontal stripe were switched. The probe session is identical to any other of the sixteen trials of the training session, but in this case, the negative stimulation does not cease when the animal enters the safe zone.

We then controlled for possible confounding variables by designing three different behavioural conditions: (i) flies reared on retinal-free food (n=14). The training and probe session were identical to the ones explained above. However, in this condition, the channel rhodopsins expressed in the bitter-sensing neurons cannot be operated by red-shifted light flashes. In this case, it was possible to assess the direct effects of the red-shifted light flashes on *Drosophila* behaviour. After ascertaining that light flashes were not distressful to the animals, the same paradigm could serve as a control experiment for assessing if there were any baseline preferences (perceptual biases) for one of the two visual markers; (ii) flies subject to constant optogenetic stimulation (n=20): in this experiment the fruit flies could not find relief in anywhere in the arena throught the whole training session (and also during the probe session). This condition was designed to evaluate if any perceptual biases were present during the negative stimulation (i.e., if the animals, when distressed, “naturally” prefer one of the two markers) during the probe session, or if any preference could be elicited through the progression of the training; (iii) flies trained in an ambiguous visual environment (n=40): under this paradigm, the animals were presented two, diametrically-opposed vertical bars, and the safe zone was moved alternately between the two. This condition was designed to evince if the fruit flies, when incapable of visual discrimation, might learn the alternation of the positions of the safe zone, as well as to evaluate if the Nearest Neighbour Rule was applied also in a context were visual learning is not feasible.

Then, we extended the results presented in the main text by (i) replicating the findings of the first set of experiments when these were run by a different experimenter (number of flies = 40); (ii) testing other flies (n = 30) under a different paradigm: during each trial, 2 seconds before the onset of optogenetic stimulation, and 4 seconds after the onset, we presented the flies solely with the homogeneously lit background, without landmarks. This set of experiments was put in place to evaluate if the flies re-oriented themselves towards the supposed location of the landmark and travelled towards the expected position, although no stripe was present (i.e., to evaluate if the re-orientation was memory-dependent).

**Statistical Analysis**

Videos acquired with the infrared camera were first uncompressed with VirtualDub 1.10.4 software, then tracked with Ctrax 0.5.18 ^46^. Tracking errors were fixed using the MATLAB FixErrorsGUI Ctrax package, and per-frame statistics were computed. Computed videos were transformed to “.txt” format with MATLAB 2018b. We then generated a dataframe for each experimental condition using RStudio 3.5.3 ^47^ and defined the localization of the safe zones in the arena. The training session was split into two sub-sets: one composed of odd-numbered trials (1^st^,3^rd^,..15^th^ trial) and the other of even-numbered trials. This division made the analysis of place preference easier since the position of the active safe zone depended on trial number (which was placed “north” during even-numbered trials, and “south” during odd-numbered trials). We defined “safe zone” (i.e. in Figure 1) as the zone where no optogenetic stimulation was active, independently from the spatial position of the zone itself. In this way, for example, the time spent in the so defined “safe zone” considered both the time spent in the southern safe zone (during odd numbered trials) and northern safe zone together. Obviously, this procedure was applied to all the variables analysed.

To test for differences in the mean number of flies in different zones (as in figure 2A) or the number of visits to different zones (as in figure 2B), we fitted the data with five different Generalised Linear Mixed Models (GLMMs) by using the R package *lme4* ^47^. Since "number of flies”/“number of visits” are counting variables, we used the Poisson family of distributions.

(GLMM1) ${\log(\mu}_{i})=\beta_{0}+u_{i}$

(GLMM2) ${\log(\mu}_{i})=\beta_{0}+\beta_{1}D_{1i}+u_{i}$

(GLMM3) ${\log(\mu}_{i})=\beta_{0}+\beta_{1}D_{2i}+u_{i}$

(GLMM4) ${\log(\mu}_{i})=\beta_{0}+\beta_{1}D_{1i}+\beta_{2}D_{2i}+u_{i}$

(GLMM5) ${\log(\mu}_{i})=\beta_{0}+\beta_{1}D_{1i}+\beta_{2}D_{2i}+\beta_{3}{D_{1i}D}_{2i}+u_{i}$

Where:

$\mu_{i}$ = incidence rate (number of flies)

$D_{1i}$ = 1^st^ categorical predictor refers to zone

$D_{2i}$ = 2^nd^ categorical predictor refers to the 10s time period during which the number of flies/number of visits was evaluated

${D_{1i}D}_{2i}$ = the interaction between the two categorical predictors

$u_{i}$ = random effects refer to the variation of the intercepts among training trials (when evaluating the number of flies) or among every single fly (for number of visits)

We compared the models using the anova function to select the best model and chose the model with the lowest BIC ^48^.

To describe the differences in performance index throughout training (as in figure 1B), we fitted data to Linear Mixed-Effects Models (LMEs).

(LME1) $Y_{i}= \beta_{0}+ \beta_{1}D_{1i} +\lambda_{i}+ \varepsilon_{i}$

Where:

$Y_{i}$ = the $i$-_th_ linear model output (performance index)

$D_{1i}$ = 1^st^ categorical predictor refers to training trials (as factor)

$\lambda_{i}$ = random effects refer to the variation of the intercepts among flies

$\varepsilon_{i}$ = error;

An analogous approach was used to assess differences in the time spent in specific areas of the arena during the probe session (as in Figure 1E). In this case, the 1^st^ categorical predictor refers to the zones (i.e., differentiating between the time spent where the safe zone was expected to be located – near the vertical bar – or where the previous safe zone was located).

When testing whether velocity changes within a zone were dependent on relief from bitter-taste stimulation (as in Figure 1C), we modelled velocity with two Linear Mixed-Effects Models: the first one was a “null” model, in which velocity changes were modelled solely based on the progression of frames since the entrance into the zone. The second model considered velocity changes to vary as a function of the interaction between relief being present (or not) and the number of frames for which the fly was inside the zone.

(LME1) $Y_{ij}= \beta_{0}+ \beta_{1}D_{1ij}+\lambda_{ij}+ \varepsilon_{ij}$

(LME2) $Y_{ij}= \beta_{0}+ \beta_{1}D_{1ij}+\beta_{2}D_{2ij}+\lambda_{ij}+ \varepsilon_{ij}$

(LME3) $Y_{ij}= \beta_{0}+ \beta_{1}D_{1ij}+\beta_{2}D_{2ij}+\beta_{3}D_{1ij}D_{2ij} +\lambda_{ij}+ \varepsilon_{ij}$

Where:

$Y_{ij}$ = the $i$-_th_ linear model output for the $j$_-th_ condition (velocity)

$D_{1ij}$ = 1^st^ continuous predictor refers to the progressive number of frames inside the zone

$D_{2ij}$ = 2^nd^ categorical predictor refers to relief

${D_{1i}D}_{2i}$ = the interaction between the two predictors

$\lambda_{ij}$ = random effects refer to the variation of the intercept among flies

$\varepsilon_{ij}$ = error;

Angle difference to a target (as in Figure 2E, 2H) is the result of the difference between the orientation of the fly and the orientation of the shortest segment joining the fly's position to that target. The orientation of the fly, and the segment orientation, were calculated in degrees, with 0 deg conventionally defined as the angle with 0 rad value. We computed two separate angle differences: one for the zone at the northern end of the arena (collapsed to a single point with coordinates 275;500 for ease of computation), and one for the zone at the southern end (collapsed to coordinates 275;50). Angle difference was thus modelled with four Linear Mixed-Effects Models (LMEs).

(LME1) $Y_{ij}= \beta_{0}+\lambda_{ij}+ \varepsilon_{ij}$

(LME2) $Y_{ij}= \beta_{0}+ \beta_{1}D_{1ij} +\lambda_{ij}+ \varepsilon_{ij}$

(LME3) $Y_{ij}= \beta_{0}+ \beta_{1}D_{1ij}+{\beta_{2}D}_{2ij}+\lambda_{ij}+ \varepsilon_{ij}$

(LME4) $Y_{ij}= \beta_{0}+ \beta_{1}D_{1ij}+{\beta_{2}D}_{2ij}+ \beta_{3}D_{1ij}D_{2ij} +\lambda_{ij}+ \varepsilon_{ij}$

$Y_{ij}$ = the $i$-_th_ linear model output for the $j$_-th_ condition (angle difference)

$D_{1ij}$ = categorical predictor refers to the period of time (before or after the onset of the bitter stimulation)

$D_{2ij}$ = categorical predictor refers to the visual marker approached

${D_{1i}D}_{2i}$ = the interaction between the two categorical predictors

$\lambda_{ij}$ = random effects refer to the variation of the intercept among trials for each fly

$\varepsilon_{ij}$ = error;

To test if the distance to the zone determines which zone the fly will enter first, we defined three binomial models (and a null model), and each triplet is tested for each zone approached (horizontal/vertical bar) and each position of the zone (at the northern or southern end of the arena).

(GLMM1) ${\log(Y}_{i})=\beta_{0}+u_{i}$

(GLMM2) ${\log(Y}_{i})=\beta_{0}+\beta_{1}D_{1i}+u_{i}$

(GLMM3) ${\log(Y}_{i})=\beta_{0}+\beta_{1}D_{1i}+\beta_{2}D_{2i}+u_{i}$

(GLMM3) ${\log(Y}_{i})=\beta_{0}+\beta_{1}D_{1i}+\beta_{2}D_{2i}+ \beta_{3}D_{1i}D_{2i}+u_{i}$

$Y_{i}$ = Zone that flies will enter (safe zone or previous safe zone)

$D_{1i}$ = 1^st^ continuous predictor refers to the distance of the animal to the zone

$D_{2i}$ = 2^nd^ continuous predictor refers to the angle difference between the fly’s orientation and the zone

${D_{1i}D}_{2i}$ = the interaction between the two predictors

$u_{i}$ = random effects refer to the variation of the intercepts among training trials for each fly

For a summary of the models’ estimates, see supplementary table S1.

For the spatial analysis of the data in panel 2D, 2G, and supplementary figure 2, we used Marcon and Puech’s M function^32-34^. This function allows testing for significant spatial aggregation of a group of points (flies) with respect to simulated random location models. The M function also allows testing whether two groups of points are spatially aggregated or segregated. To test for the latter condition, the spatial relationship between the two groups of flies is compared to simulations of random labelling models (i.e., the points – flies positions – are randomly assigned to one of the two groups). To test for the significance of spatial aggregation, we executed 10.000 Monte Carlo simulations of the null models (random location/random labelling) and compared them to the observed data. In both cases, a goodness-of-fit test reported the significance of the findings.

All graphs were produced using the ggplot2 ^49^ and ggpubr ^50^ packages.

The MATLAB^®^ customised script is available at <http://dx.doi.org/10.17632/rbbtnwgr28.2>

Datasets are available at <https://data.mendeley.com/datasets/9frwpy5vz9/draft?a=5e9ec4cc-7e52-44d7-af0d-38718078de0f> (definitive DOI will be provided after review).


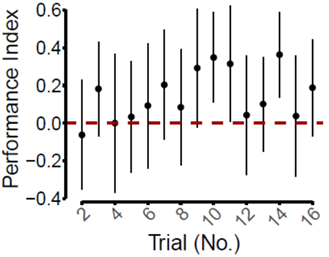

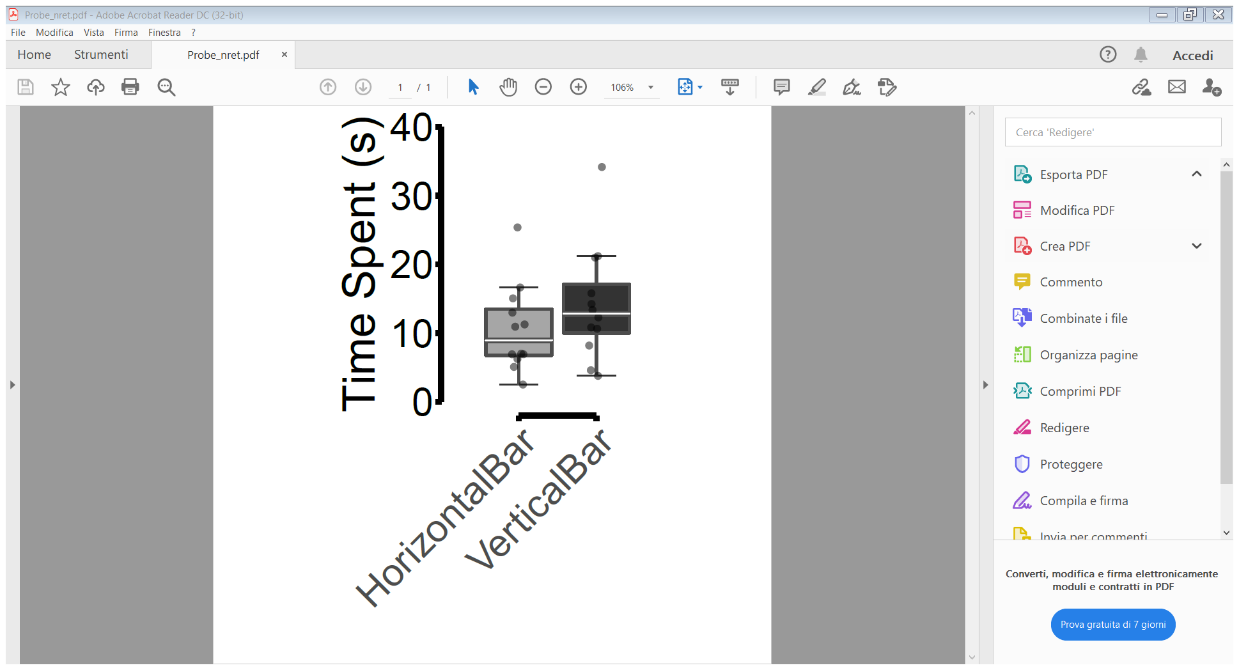

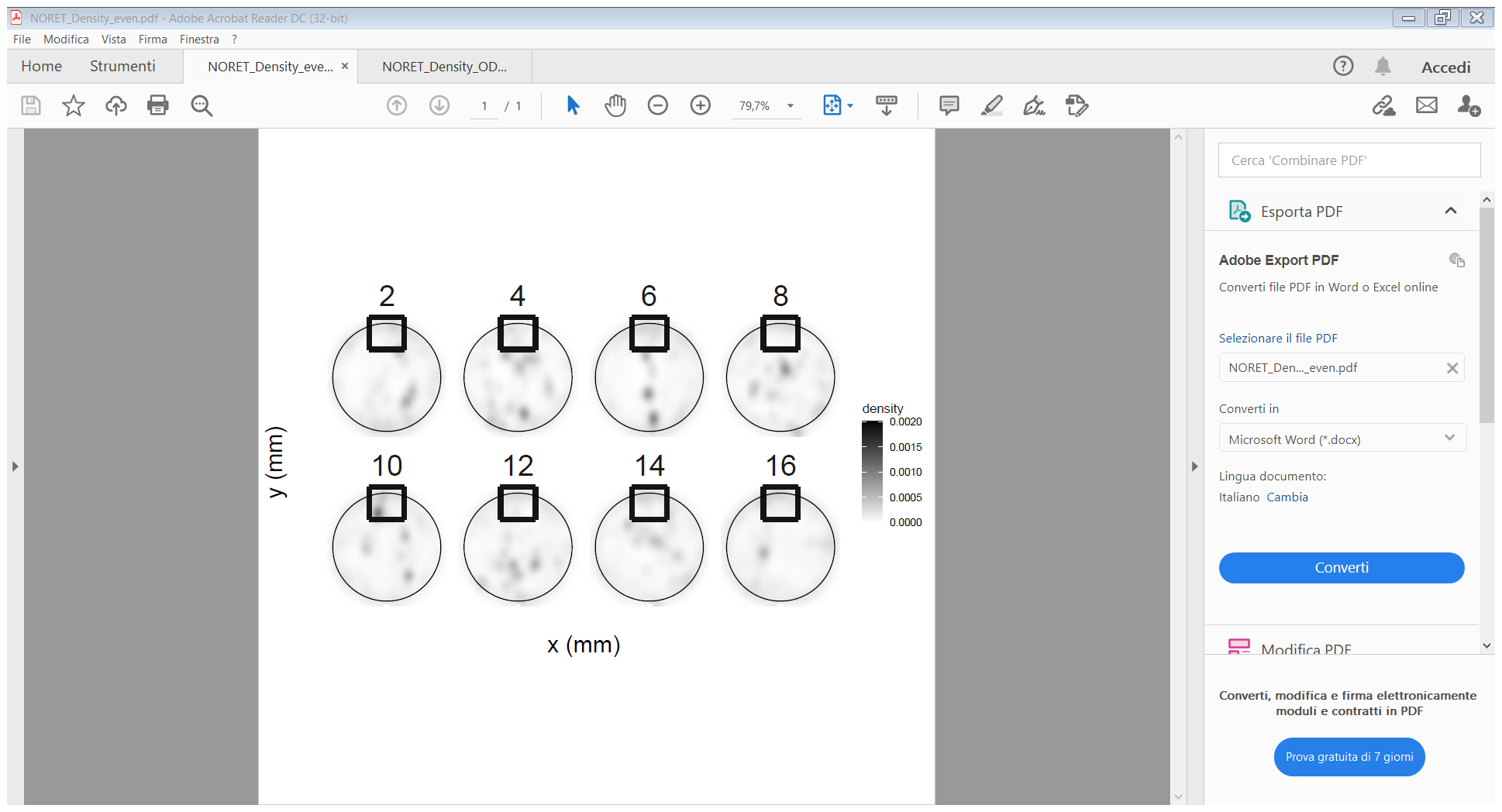

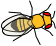

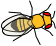

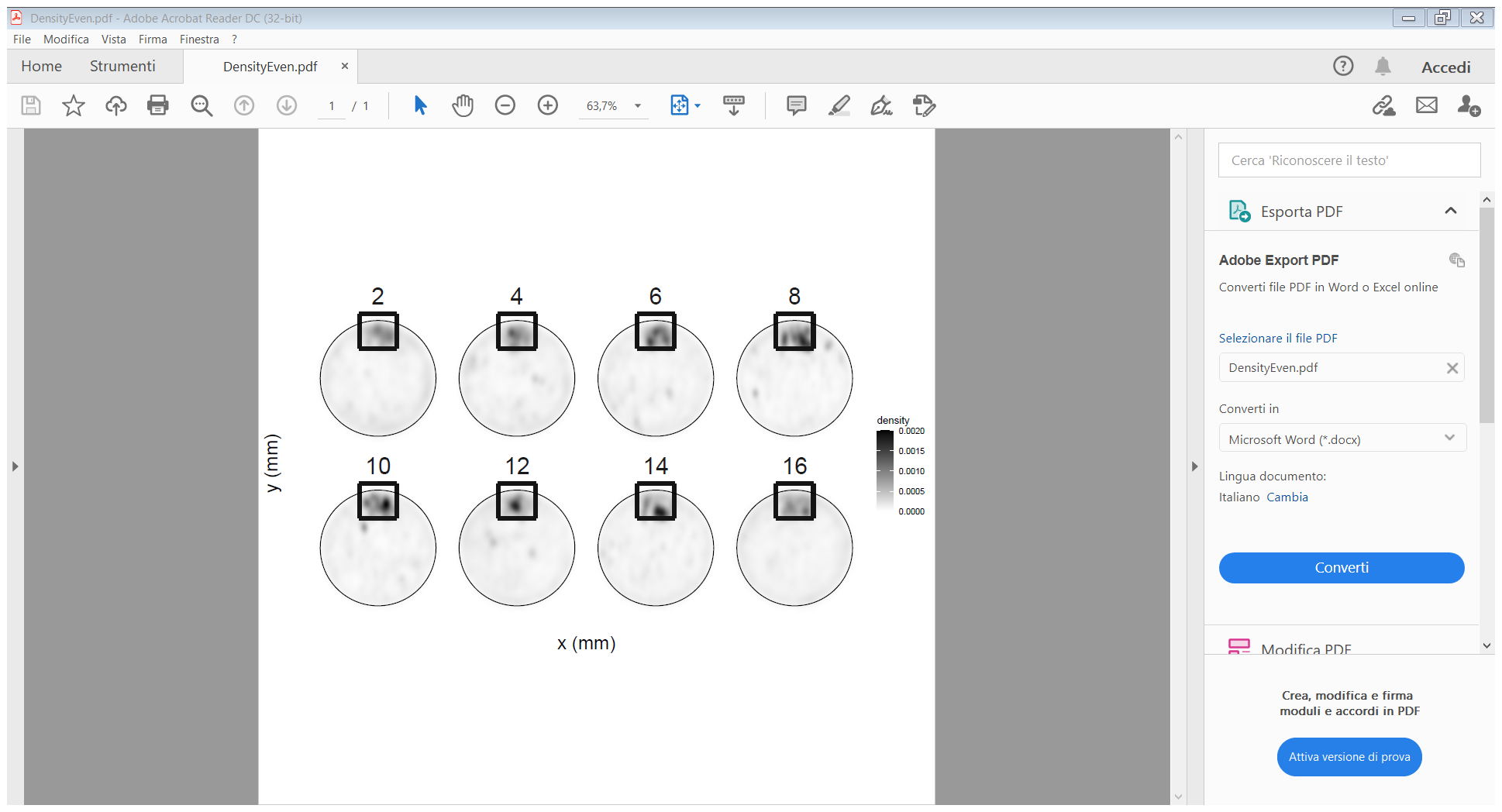

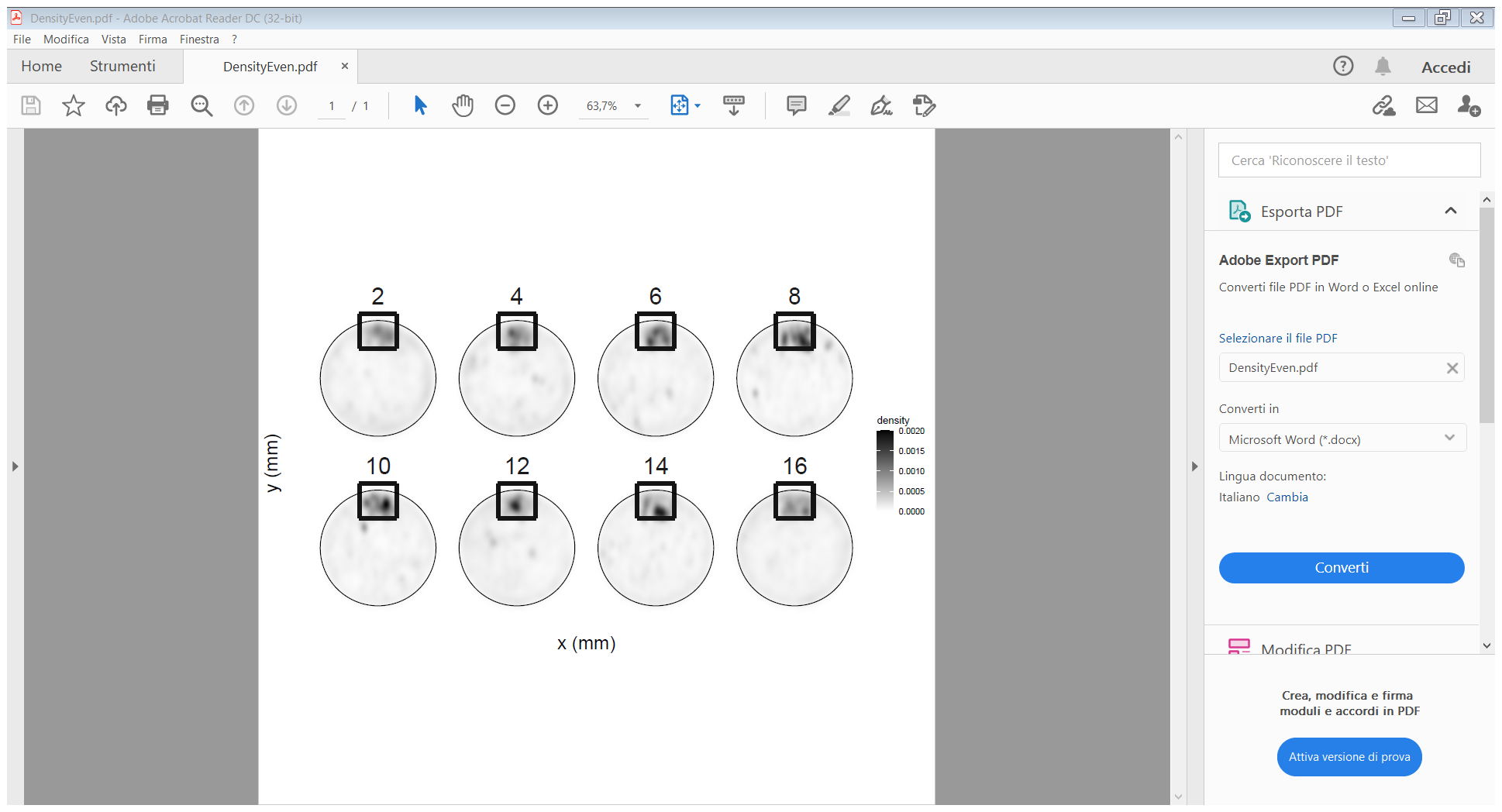

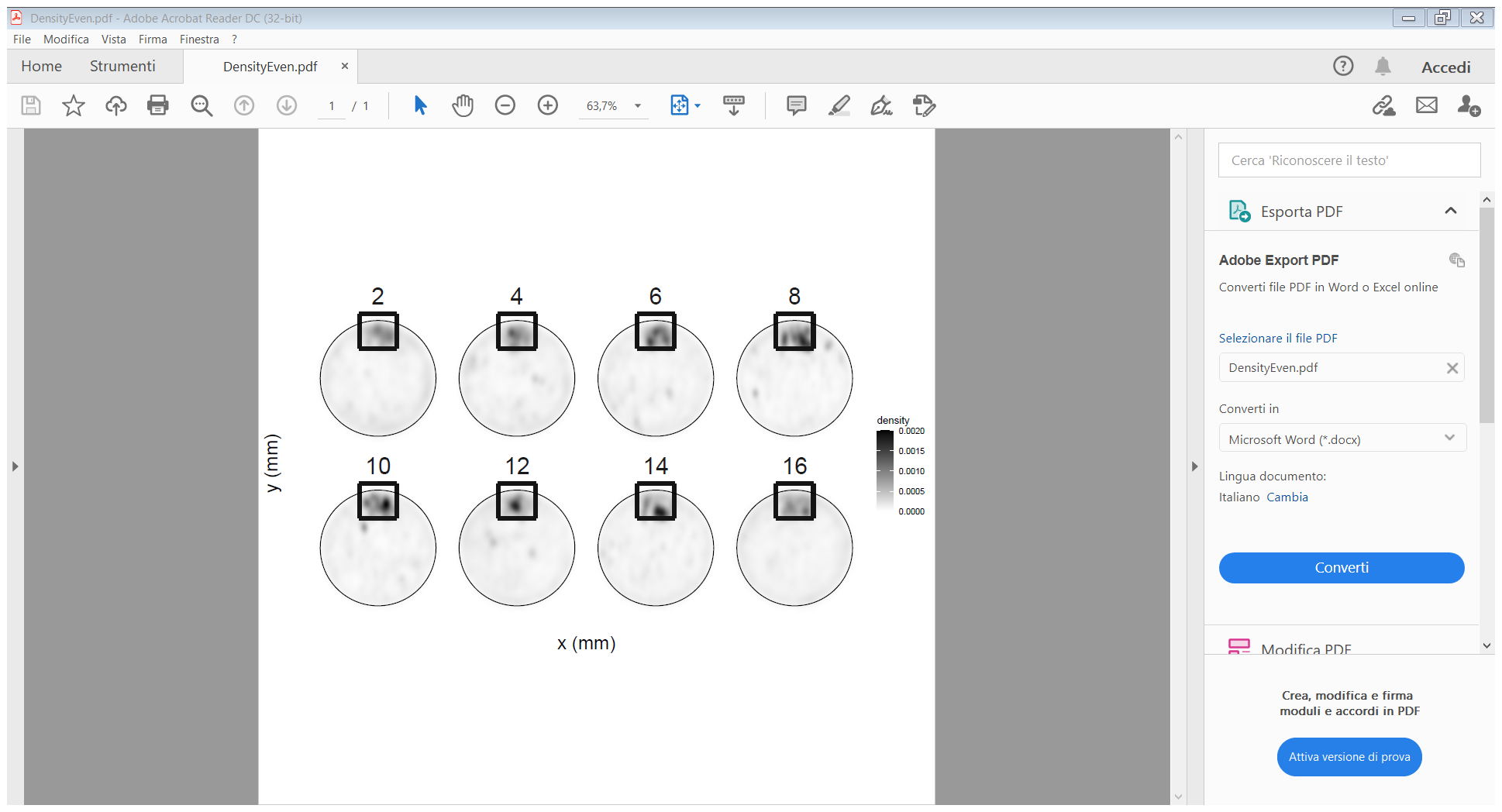

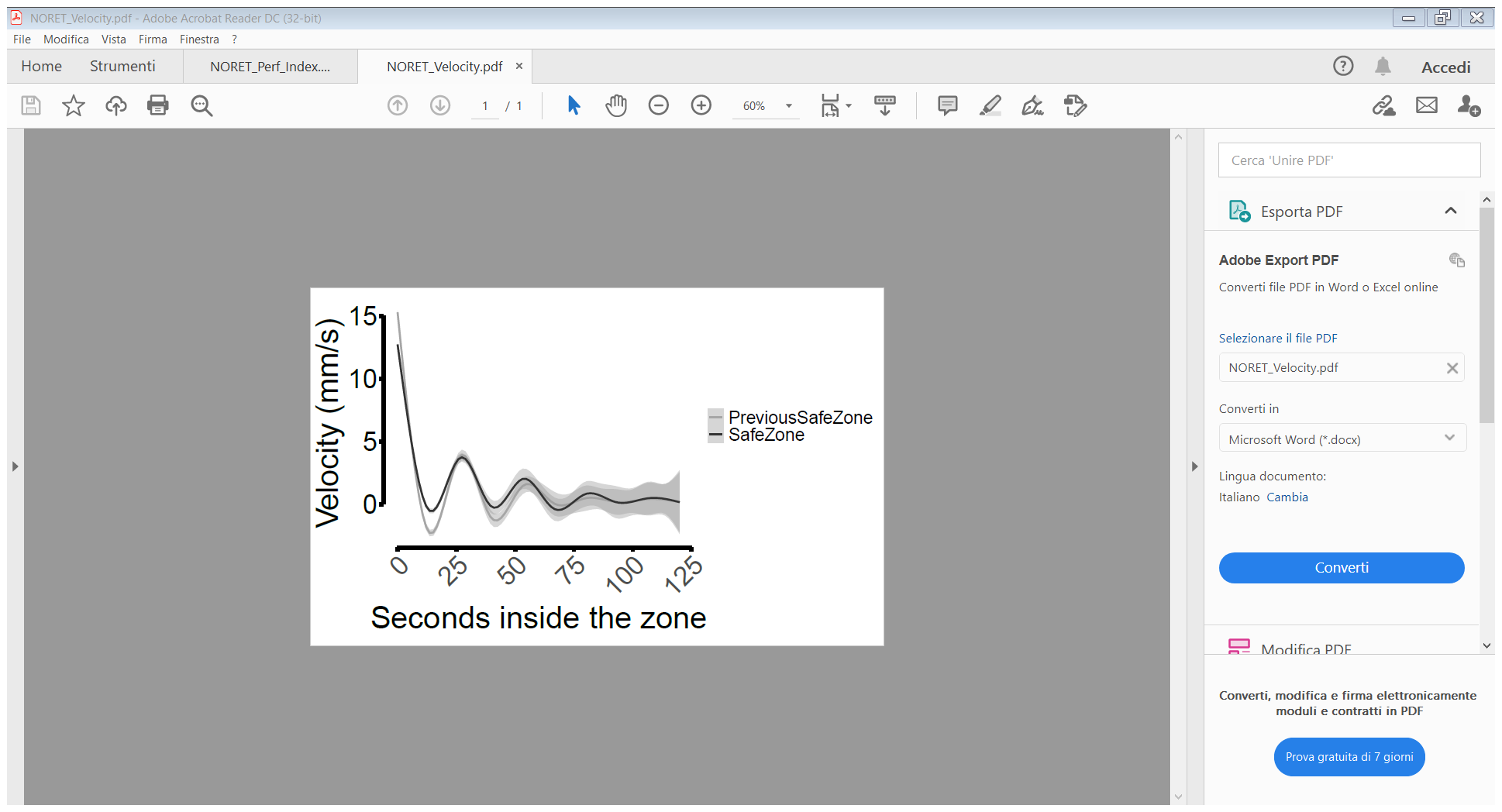

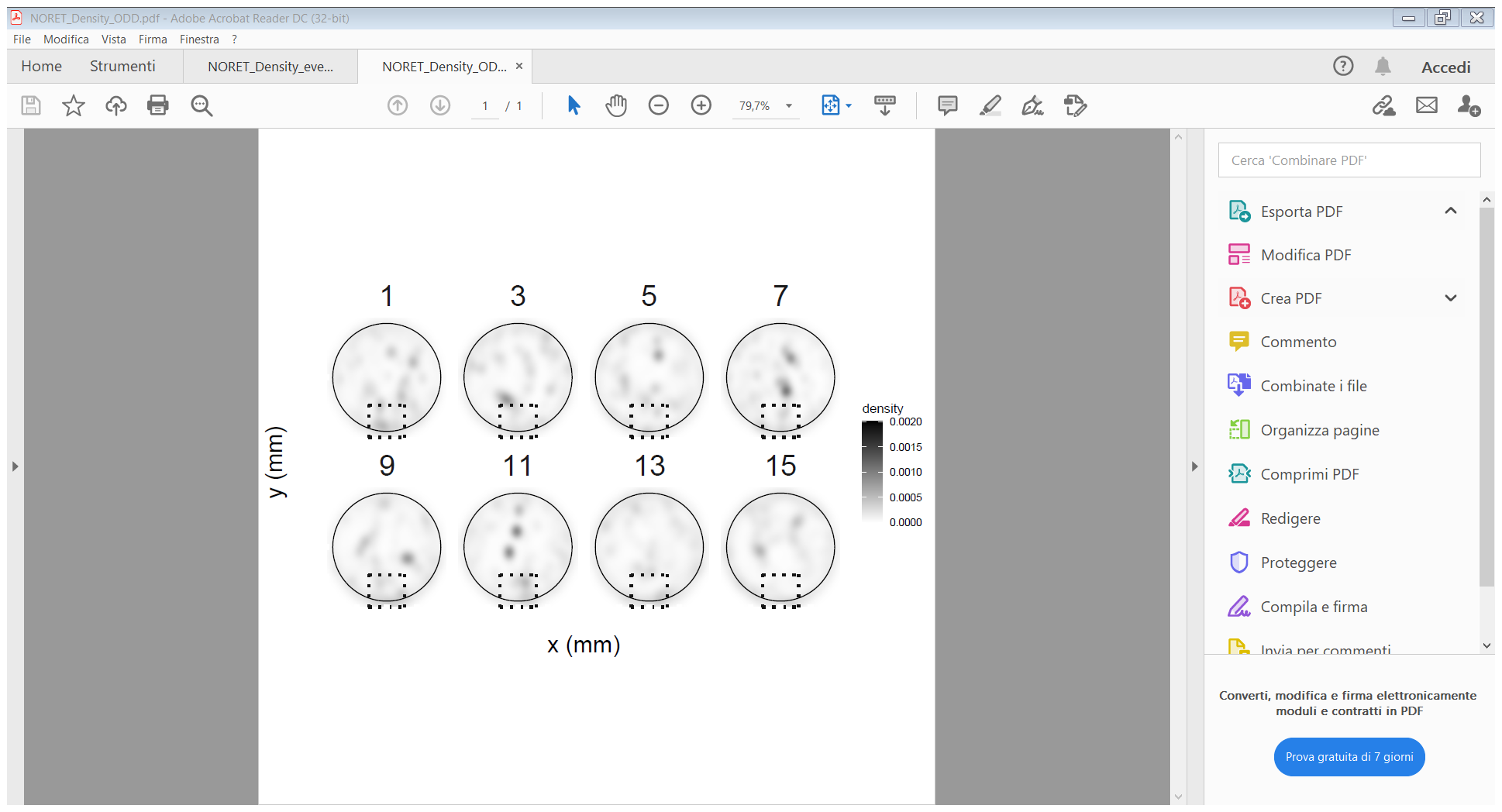

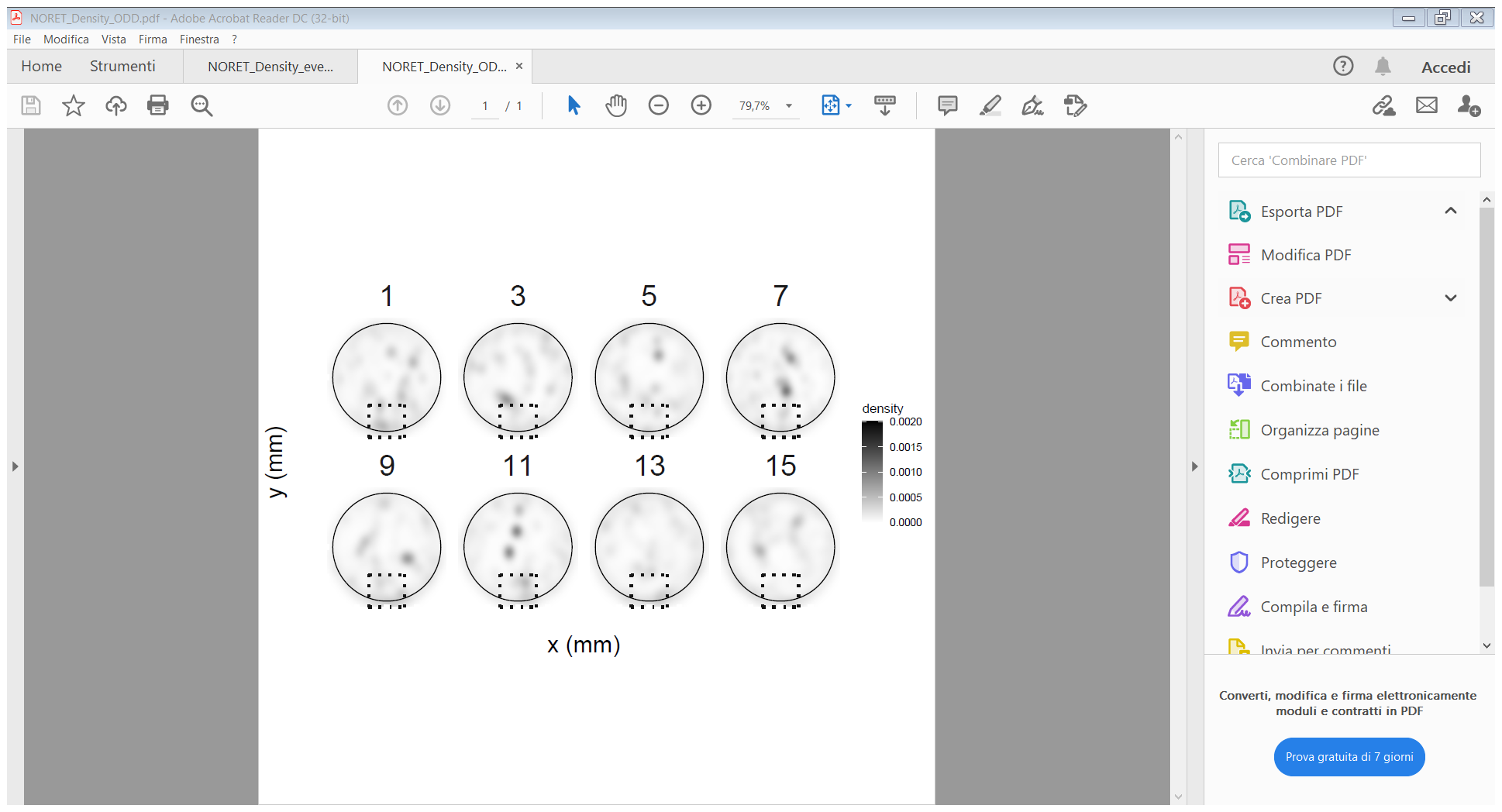

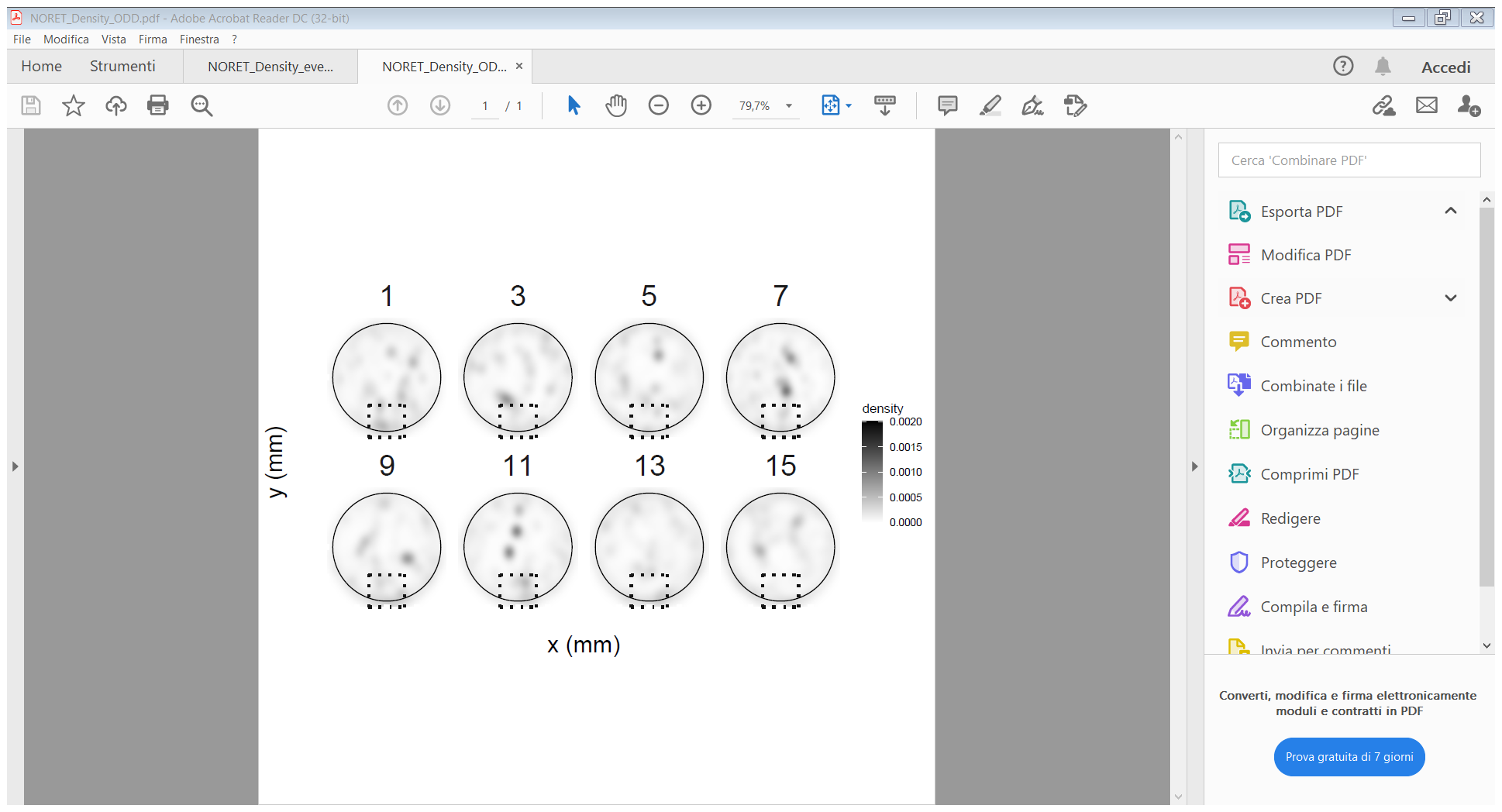

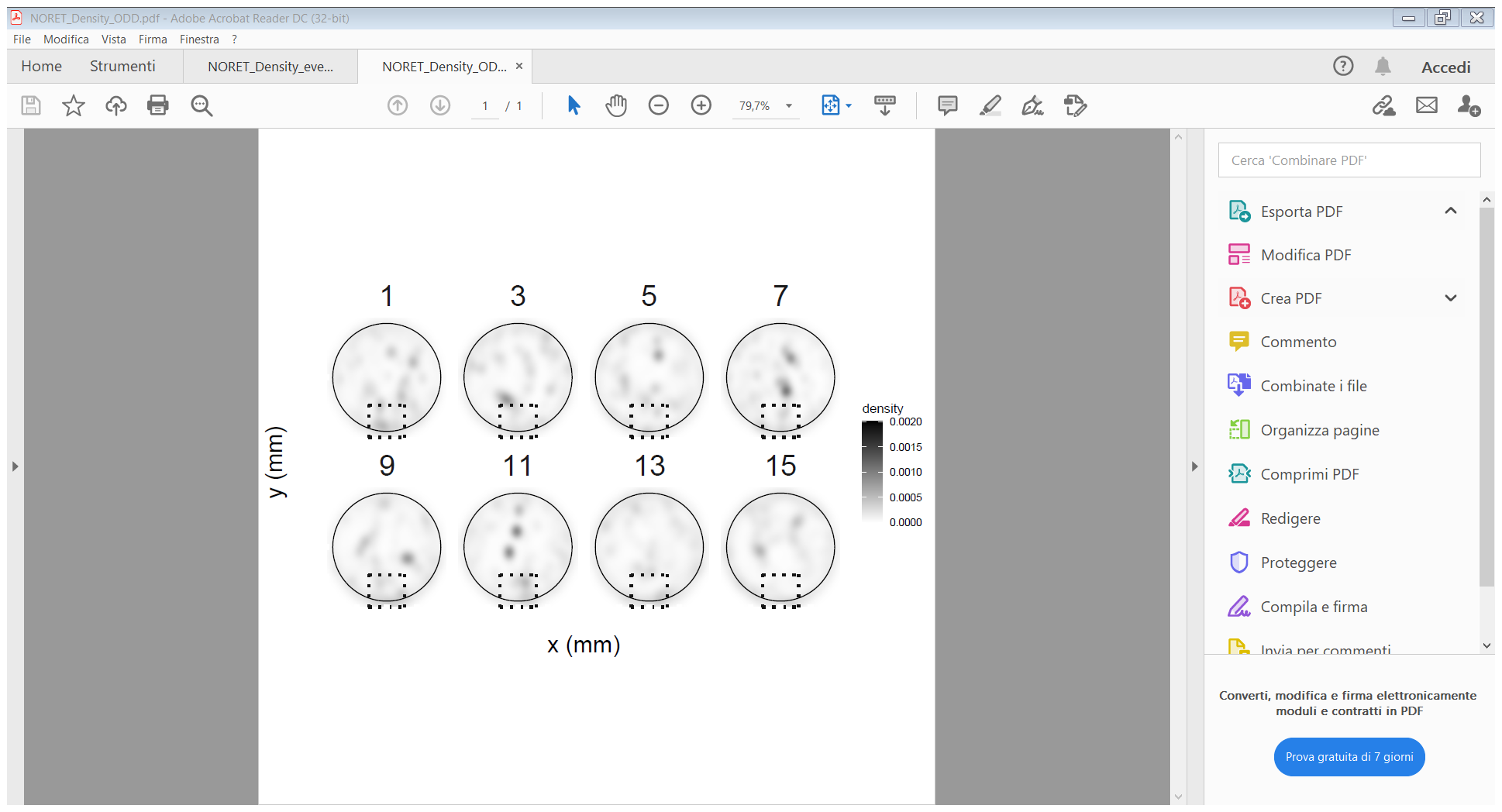

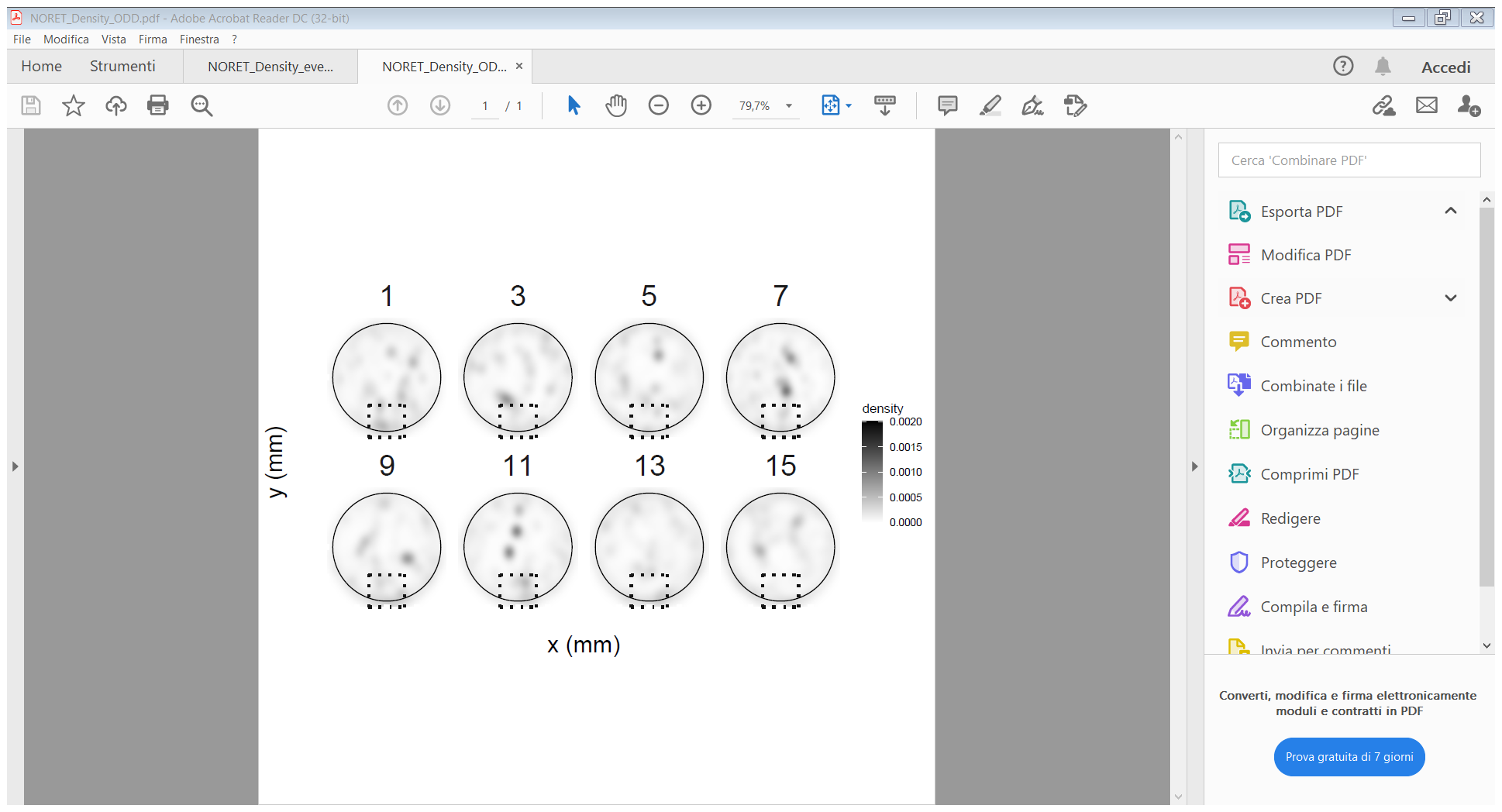

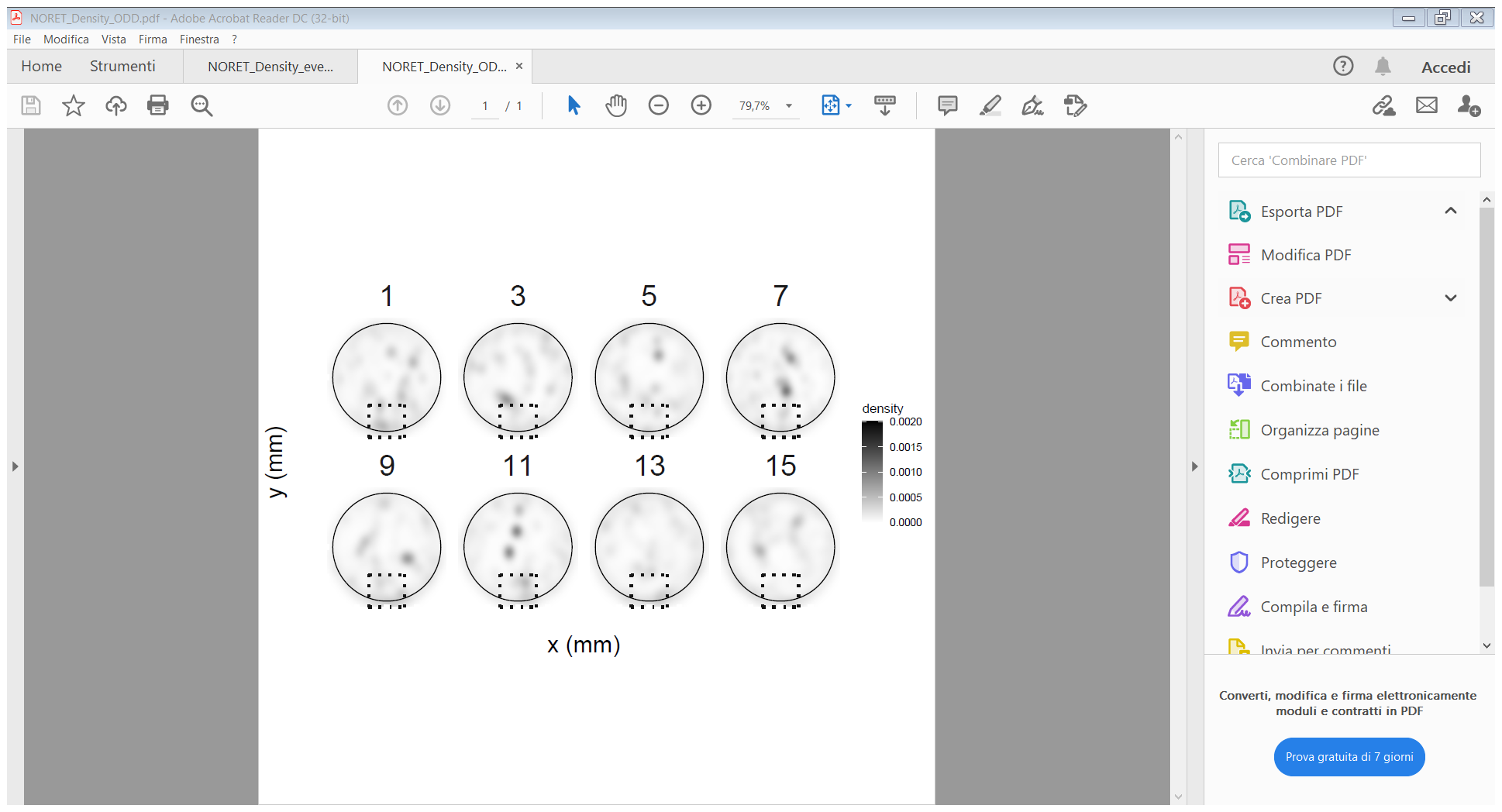

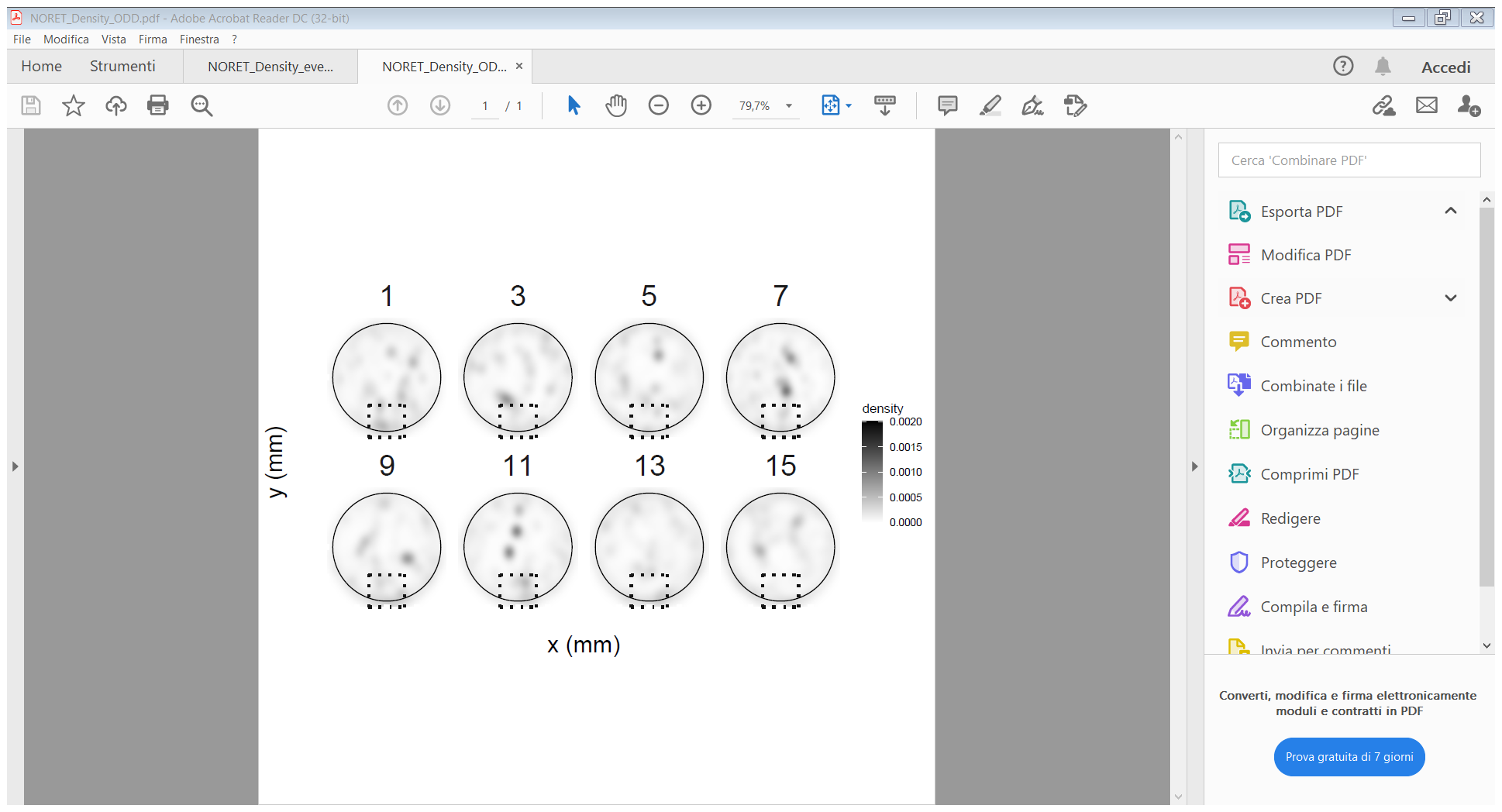

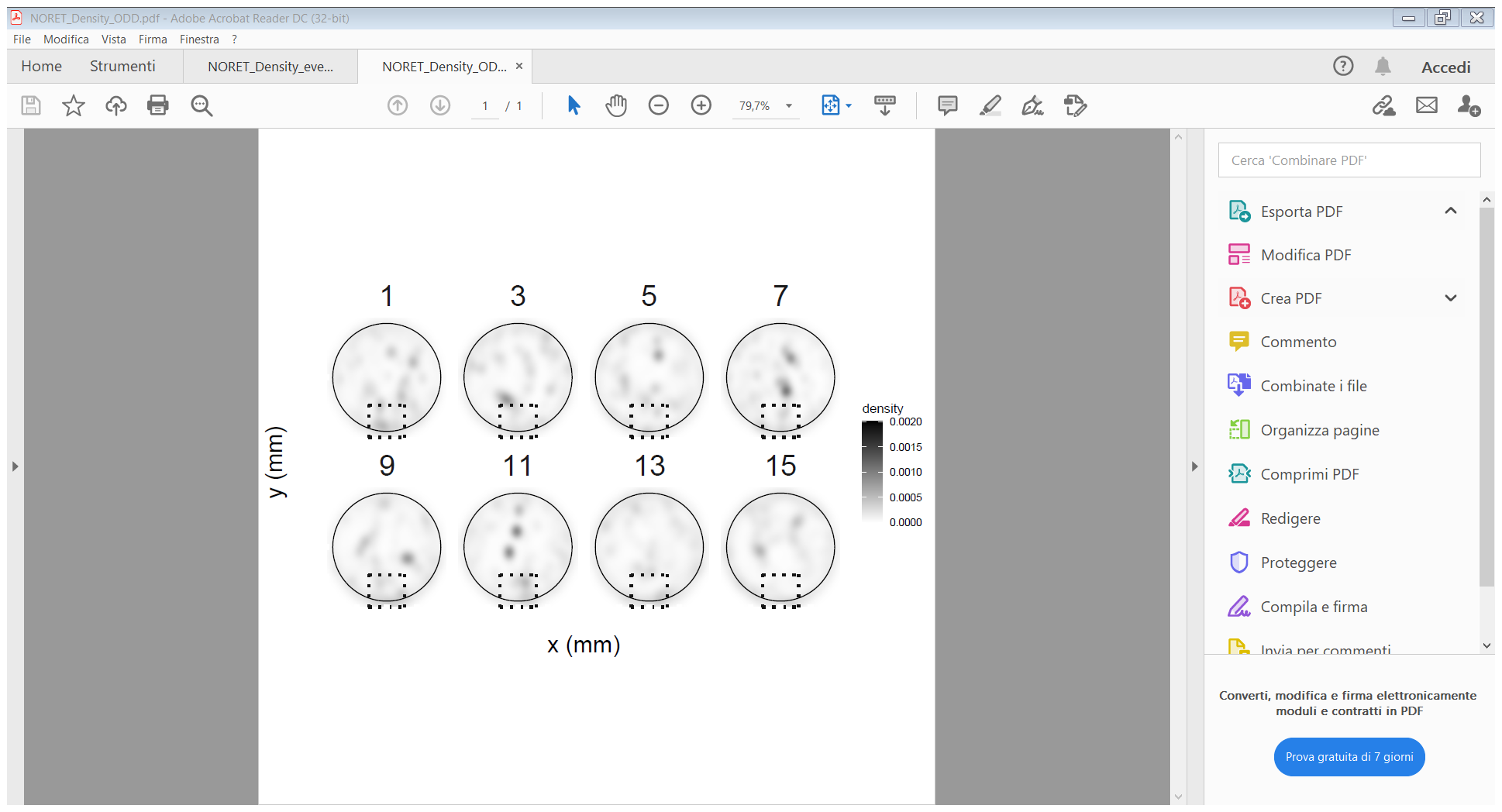

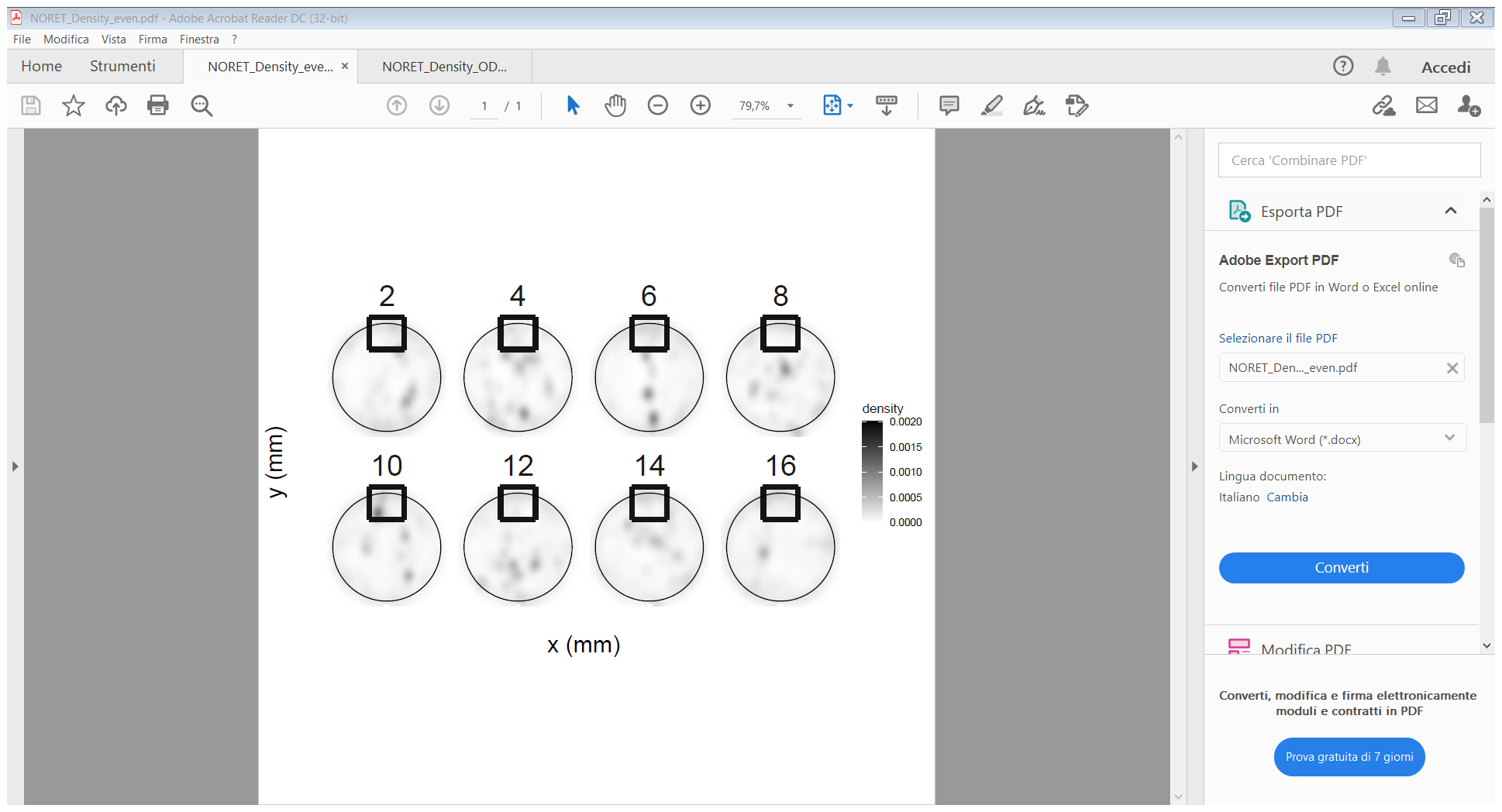

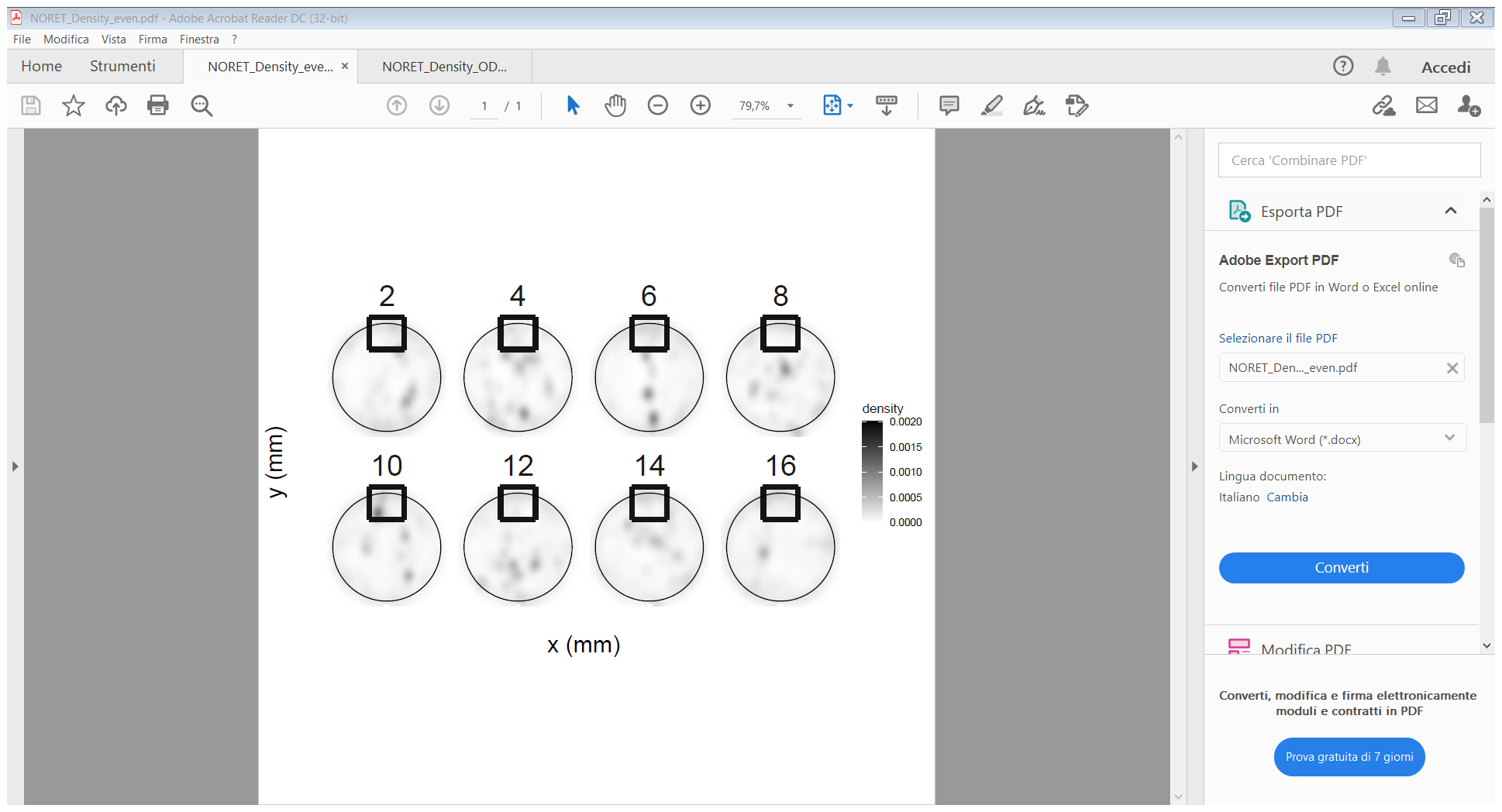

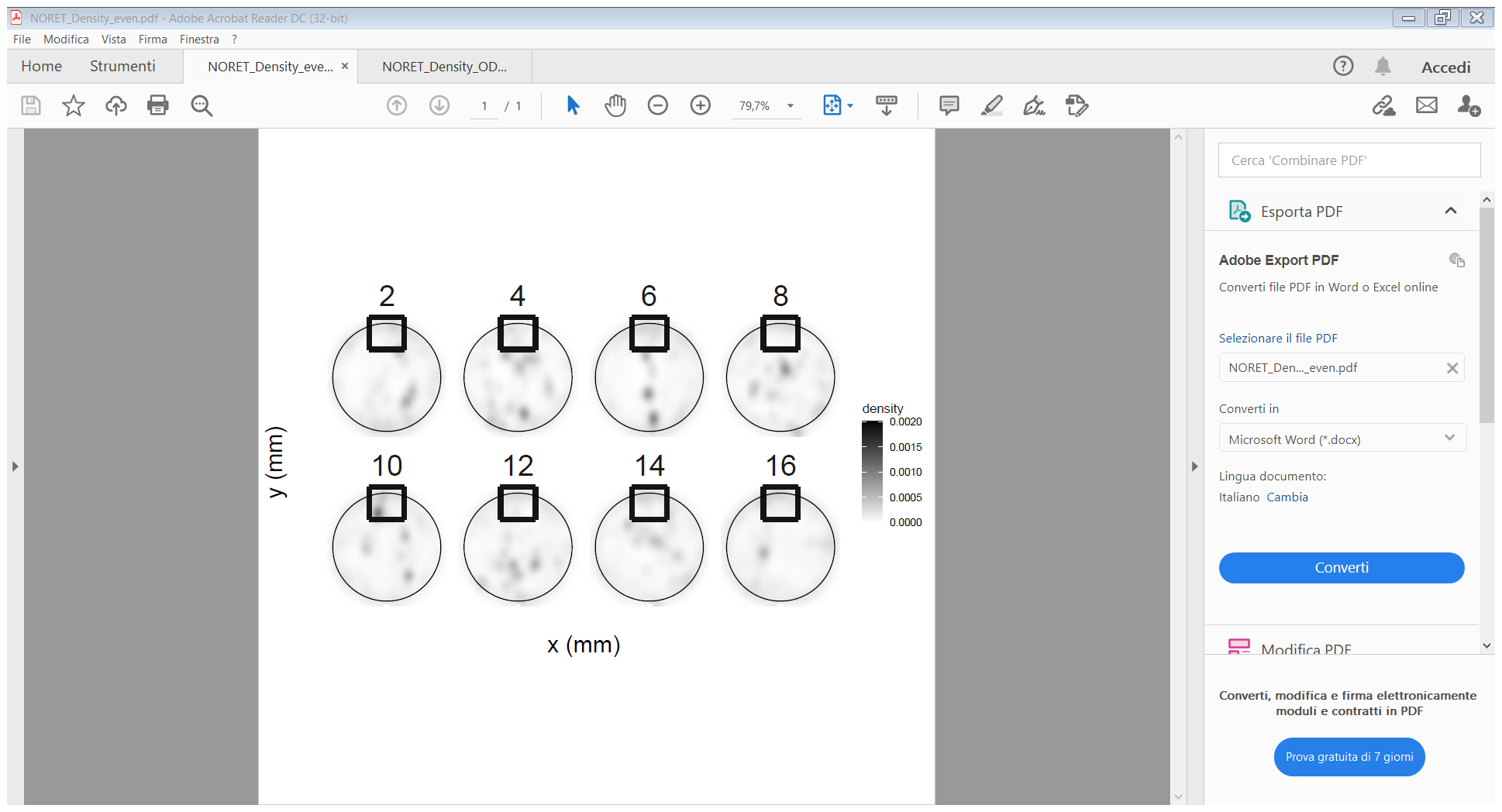

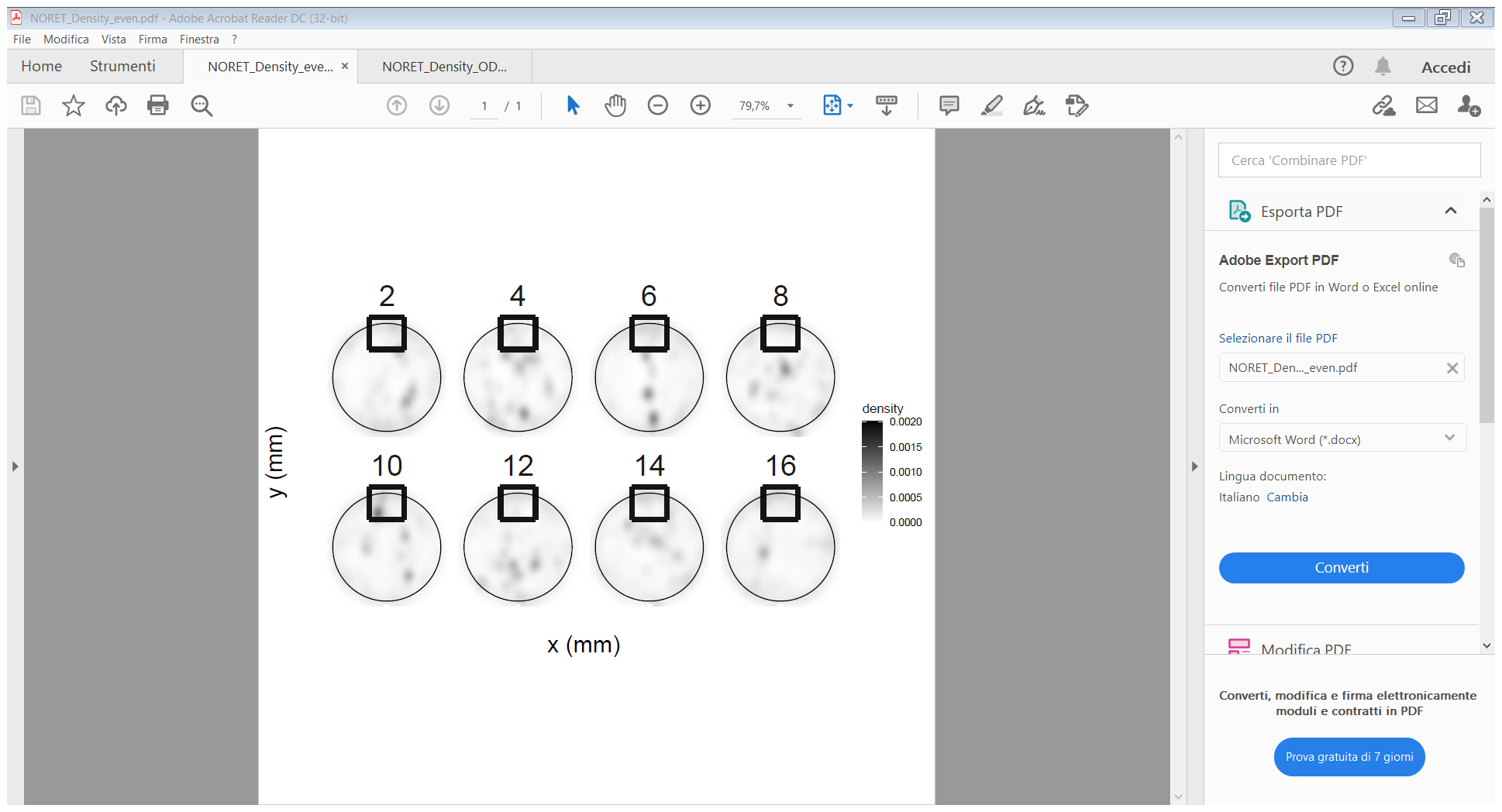

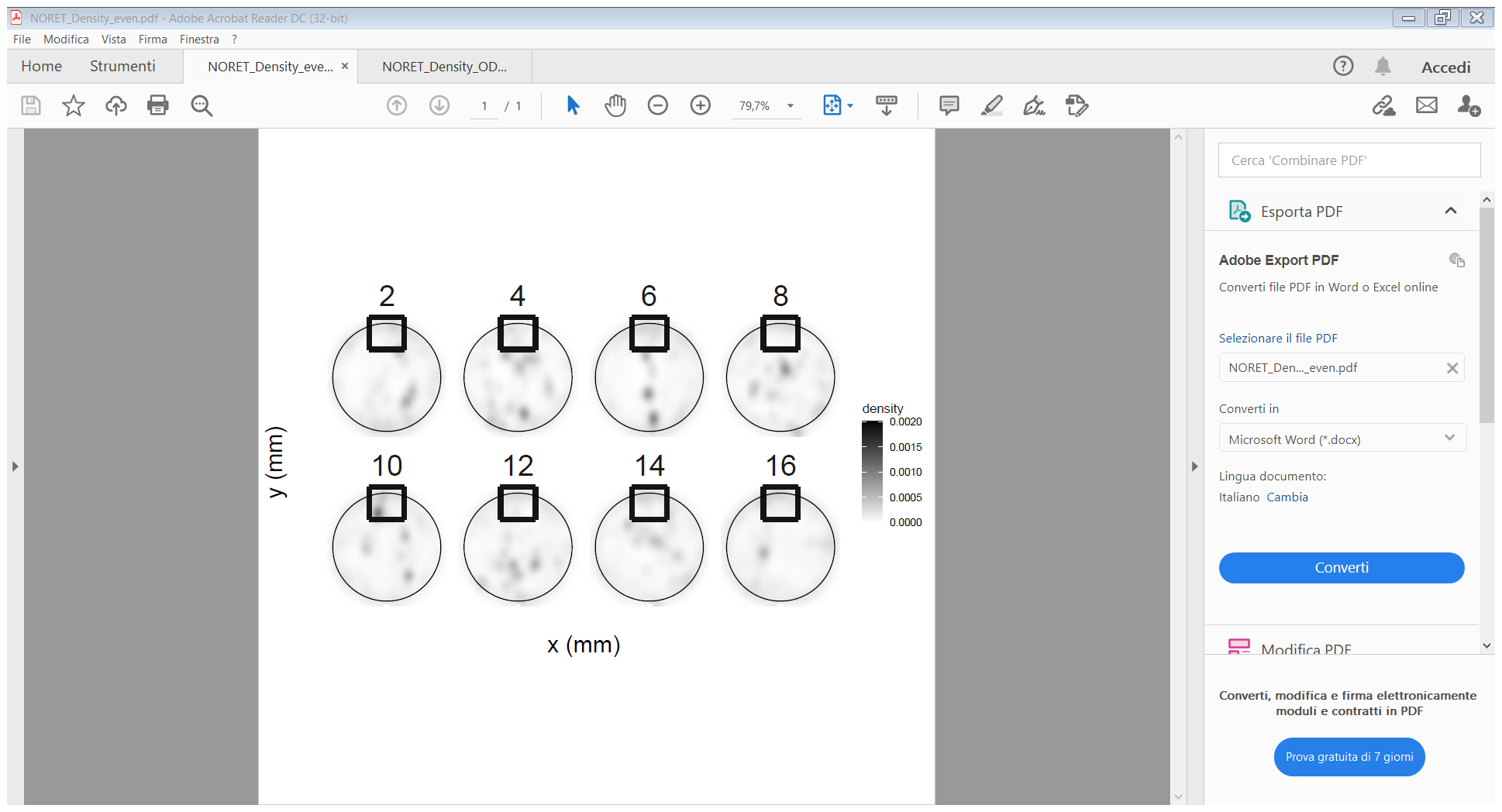

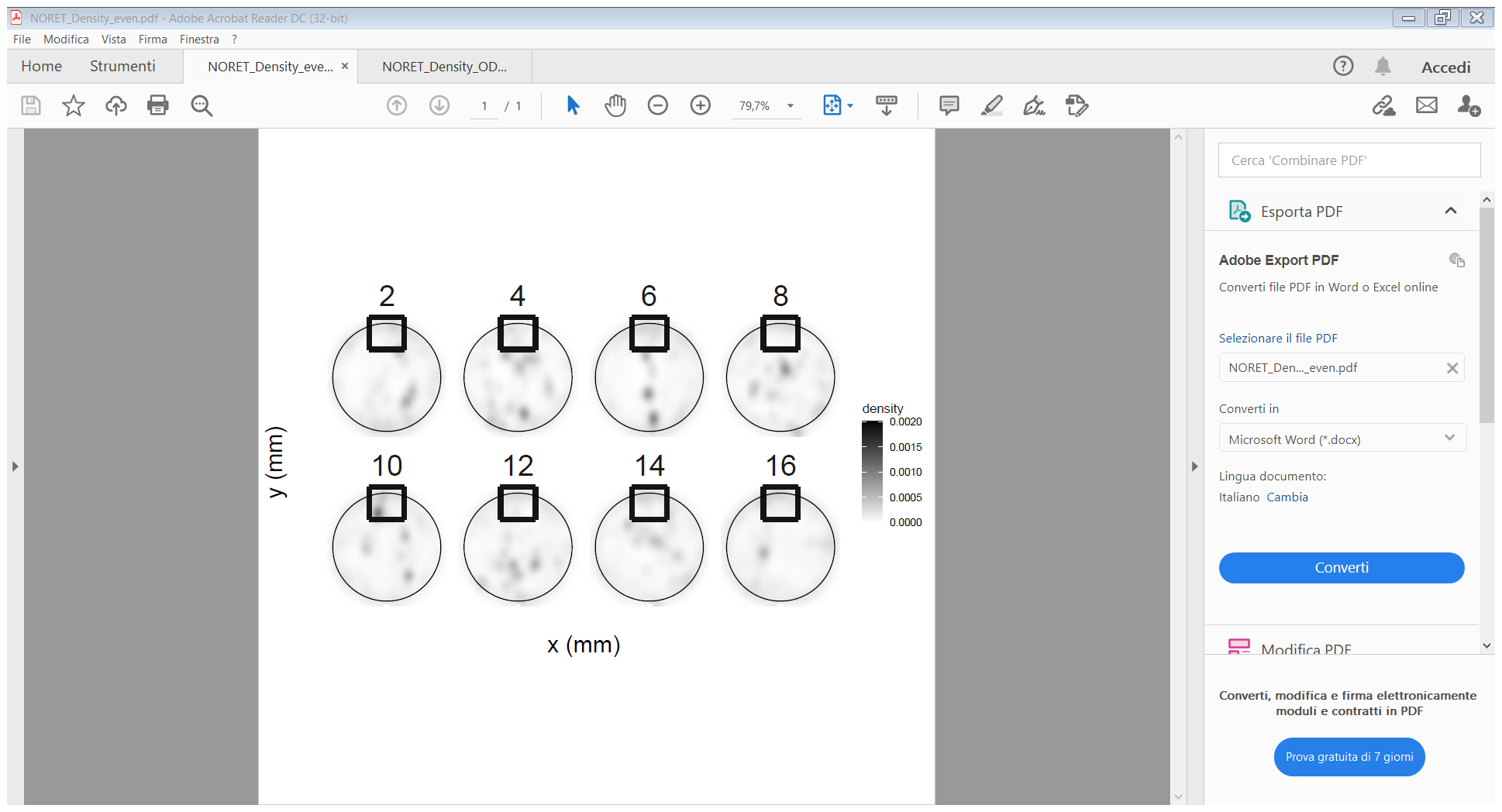

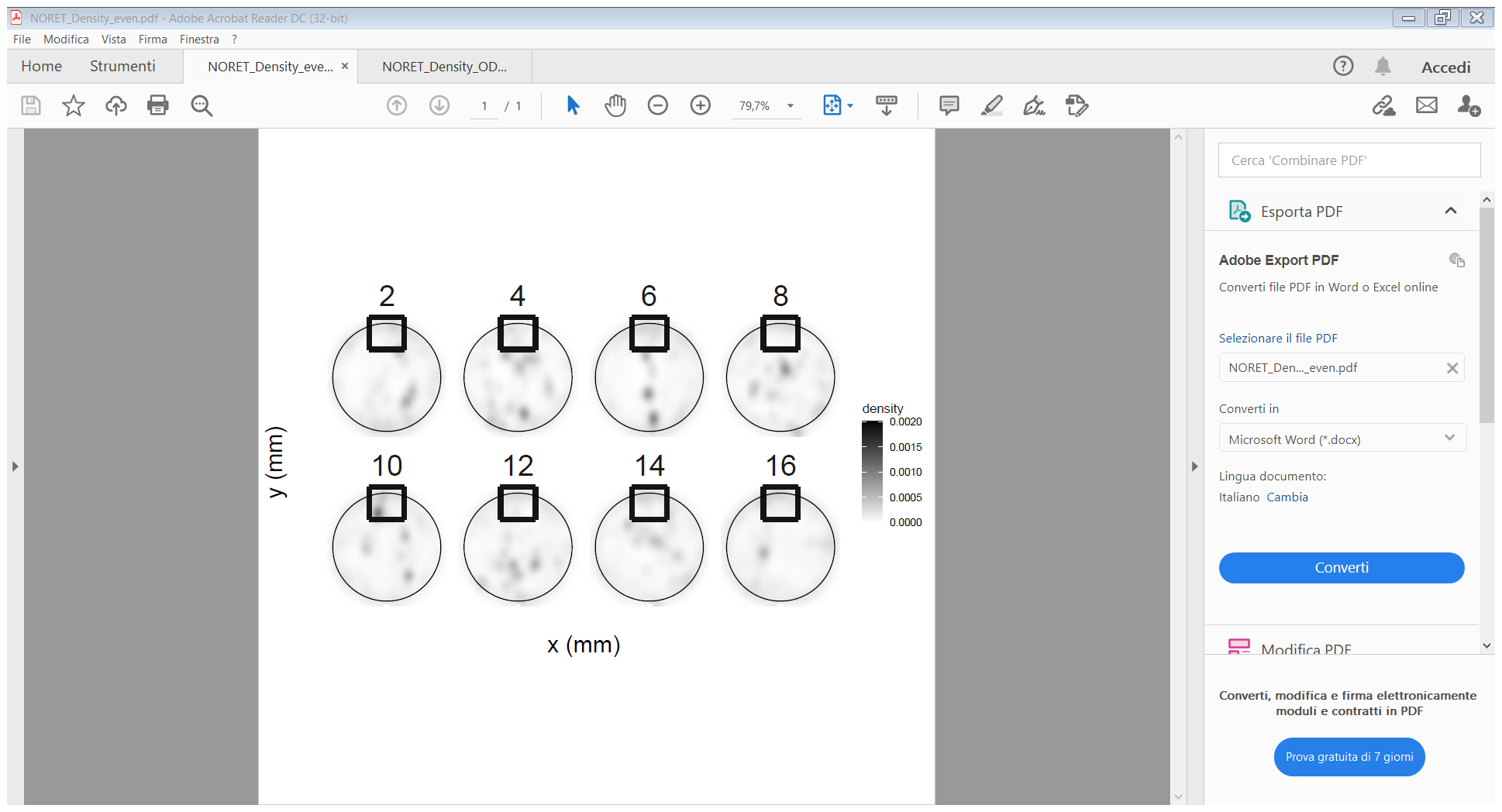

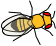

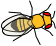

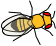

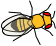


**D**

residency

Trial 1-4

Trial 5-8

Trial 9-12

Trial 13-16

**C**

**Food w/out retinal**

**A**

**B**

**E**

**Supplementary Figure S1. Flies reared on food without retinal are not affected by red light stimulation.**

Black bars = represent the vertical or horizontal stripe;

**A)** Data presented in this figure are related to a subgroup of flies expressing red-shifted rhodopsins in bitter-sensing neurons (n = 14), but this group of flies was not reared on retinal-enriched food (thus making the rhodopsin unresponsive to photons) **B)** Performance Index (PI) as a function of training trials. Pointrange = mean ± confidence interval around the mean. The 0 value marks indifference in the time spent close to the vertical or horizontal bar. PI was not computed for trial 1 given the absence of a Previous Safe Zone during that trial. Flies did not grow a significant preference for the zone where no light stimulation is available (vertical bar) throughout training (see also Table S1); **C)** Velocity profile of fruit flies after entering a zone where light stimulation was shut off (black line) compared to the profile (grey line) after entering a zone where light stimulation was continued (previous safe zone). Gray shadow represents 95% CI. Velocity inside the previous safe zone (i.e., with red-light flashing) were lower than inside the safe zone (no. observed velocities = 11202, mean difference = 0.177, std error = 0.045, z.ratio = 3.865, p < 0.0001) **D)** density plots describing the residency of flies during training session, when the light stimulation was switched on if flies left the squared zones. This group of flies did not display a higher residency in the safe zone, on the contrary of animals reared on retinal-enriched food (see also main text Figure 1); **E)** During the probe session, fruit flies spent an equal amount of time close to the vertical and horizontal bar (no. of comparisons = 25, mean difference in time spent (s) = 6.38, std error = 4.11, t.ratio = 1.55, p = 0.14), further showing that this group of flies did not learn the vertical bar – relief association, since these animals could not be stimulated by red light flashes.

|  | **Performance index** |  | **Performance index** |
| --- | --- | --- | --- |
| *Predictors* | *Estimates, mean (CI 95%)* | *Predictors* | *Estimates, mean (CI 95%)* |
| (Intercept) | -0.06  (-0.32 – 0.19) | ntrial [10] | 0.41 ^*^ (0.06 – 0.76) |
| ntrial [3] | 0.24  (-0.11 – 0.60) | ntrial [11] | 0.36  (-0.00 – 0.72) |
| ntrial [4] | 0.06  (-0.29 – 0.42) | ntrial [12] | 0.10  (-0.25 – 0.46) |
| ntrial [5] | 0.10  (-0.25 – 0.44) | ntrial [13] | 0.16  (-0.19 – 0.52) |
| ntrial [6] | 0.15  (-0.20 – 0.51) | ntrial [14] | 0.43 ^*^ (0.08 – 0.77) |
| ntrial [7] | 0.25  (-0.11 – 0.60) | ntrial [15] | 0.10  (-0.26 – 0.45) |
| ntrial [8] | 0.13  (-0.23 – 0.49) | ntrial [16] | 0.25  (-0.10 – 0.60) |
| ntrial [9] | 0.34  (-0.03 – 0.70) |  |  |
| Flies tested = 14 | | | |
| ** p<0.05   ** p<0.01   *** p<0.001* | | | |

**Table S1 related to supplementary Figure S1B. Performance index, for each trial, for flies reared on food without retinal.** ntrial = trial number. CI 95% = 95% confidence interval. The estimates for the intercept and ntrial[3],…, ntrial[16] are the output of a linear mixed-effects model that describes the performance index (PI) as a function of trials, for each animal. PI was not computed for trial 1 given the absence of a Previous Safe Zone during that trial. The performance index during ntrial [2] is used as the reference value to compute the performance index estimates during the remaining ntrials.


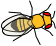

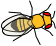

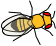

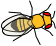

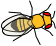


**A**

**B**

**C**

**D**

new probe-like trial

x 17

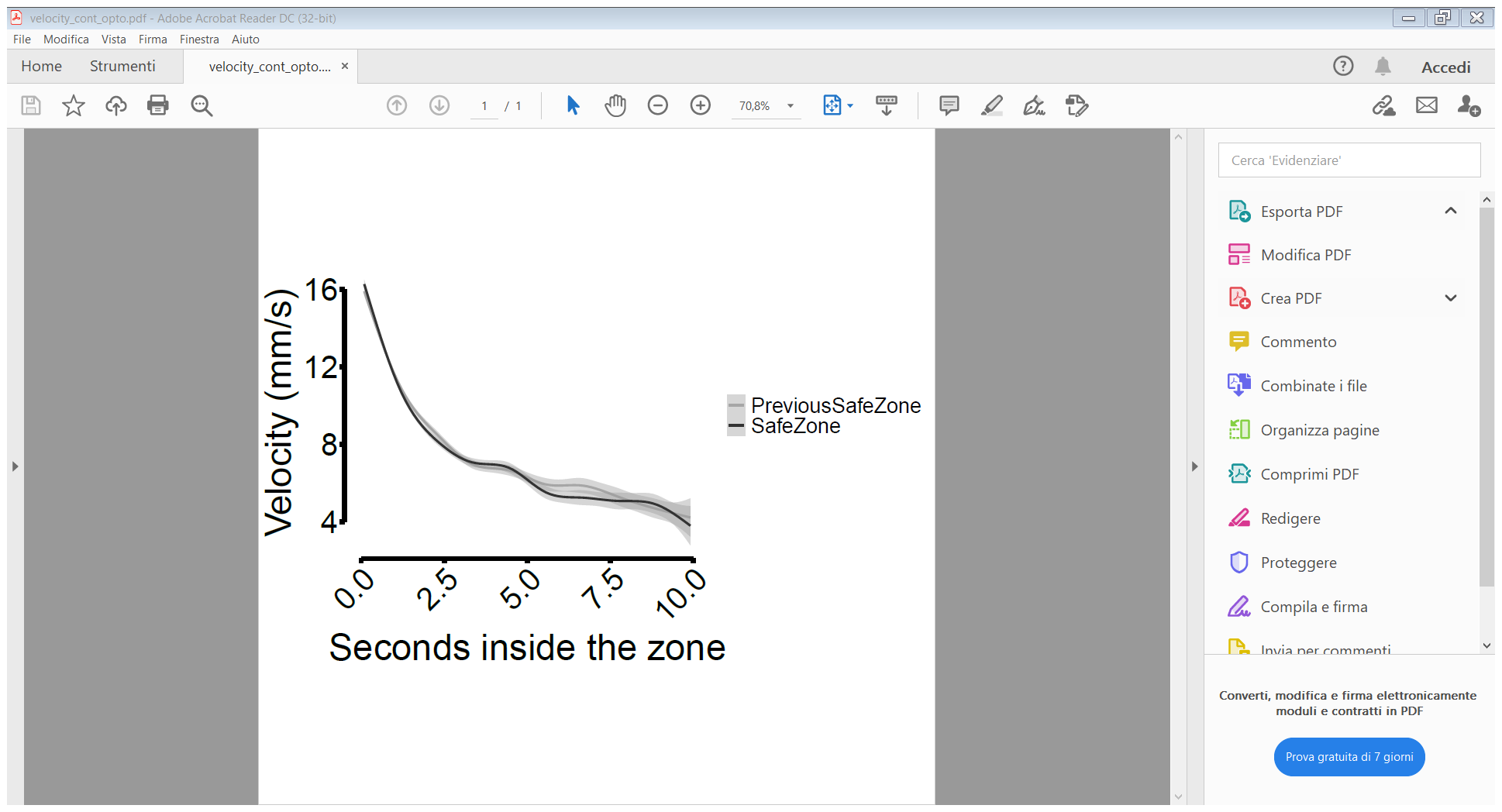


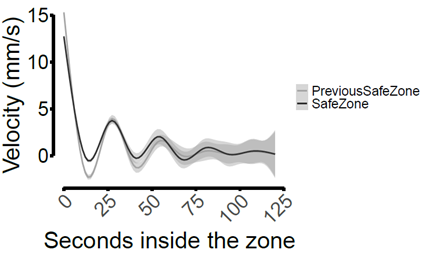


**E**


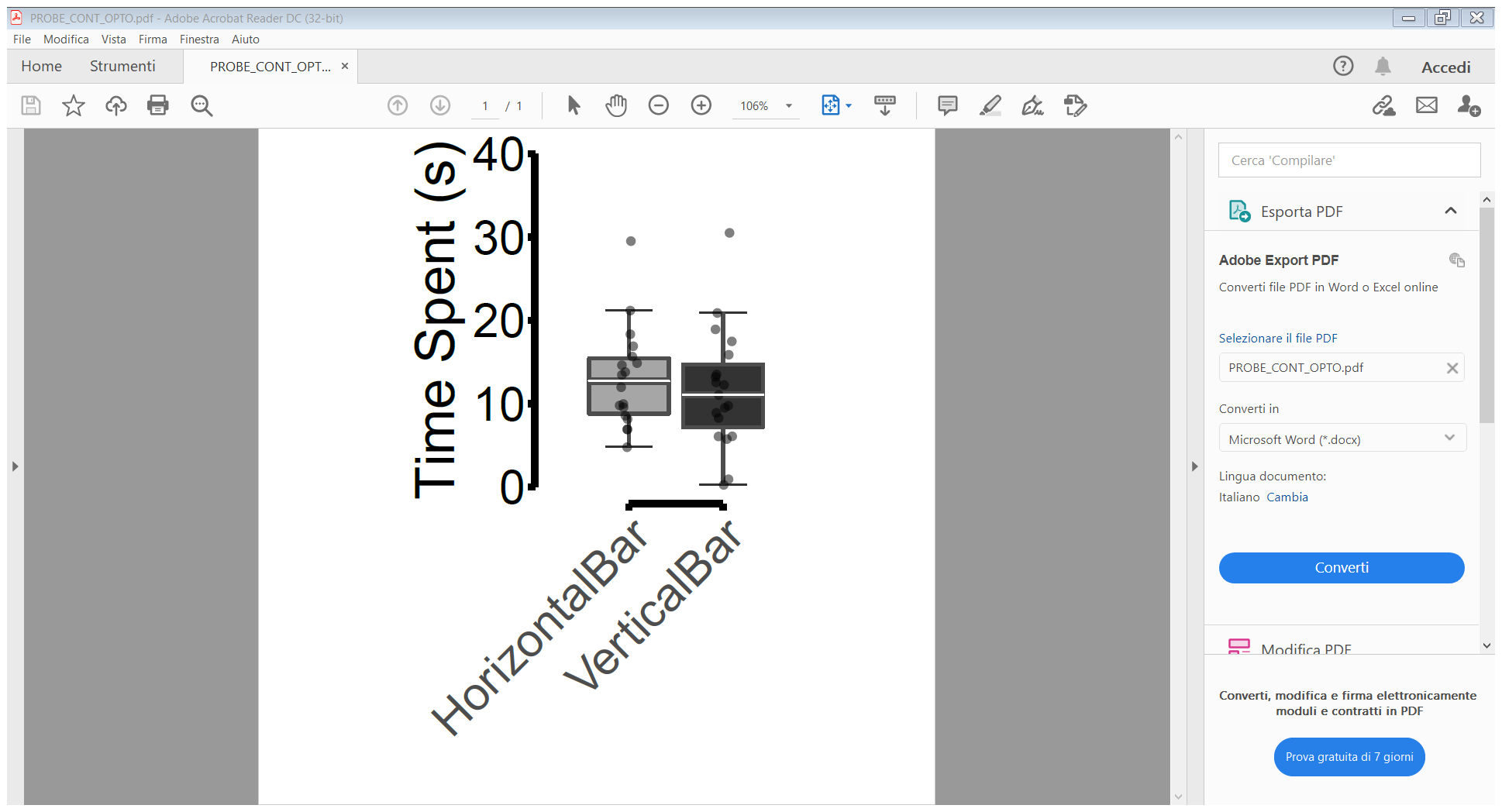

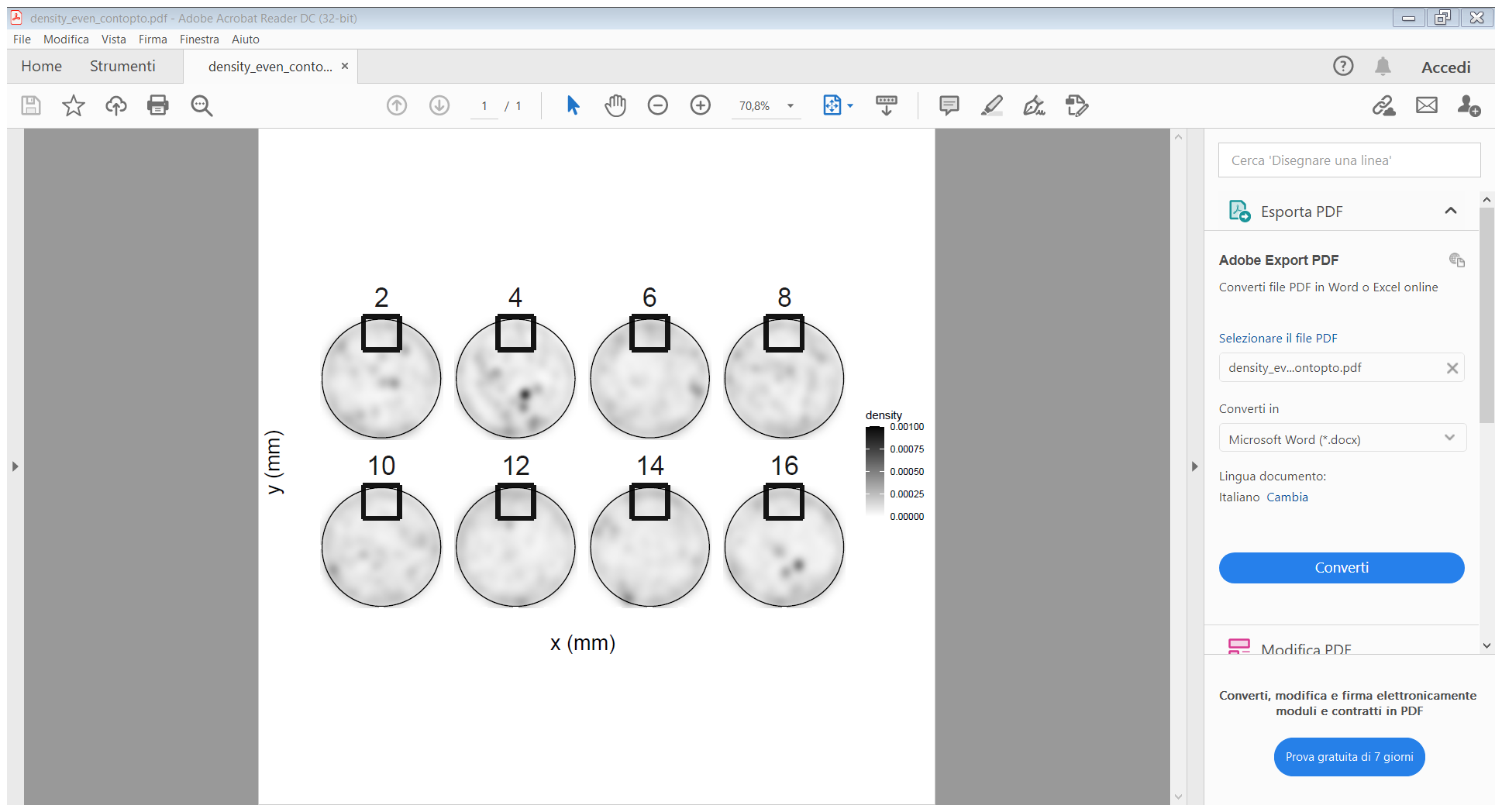

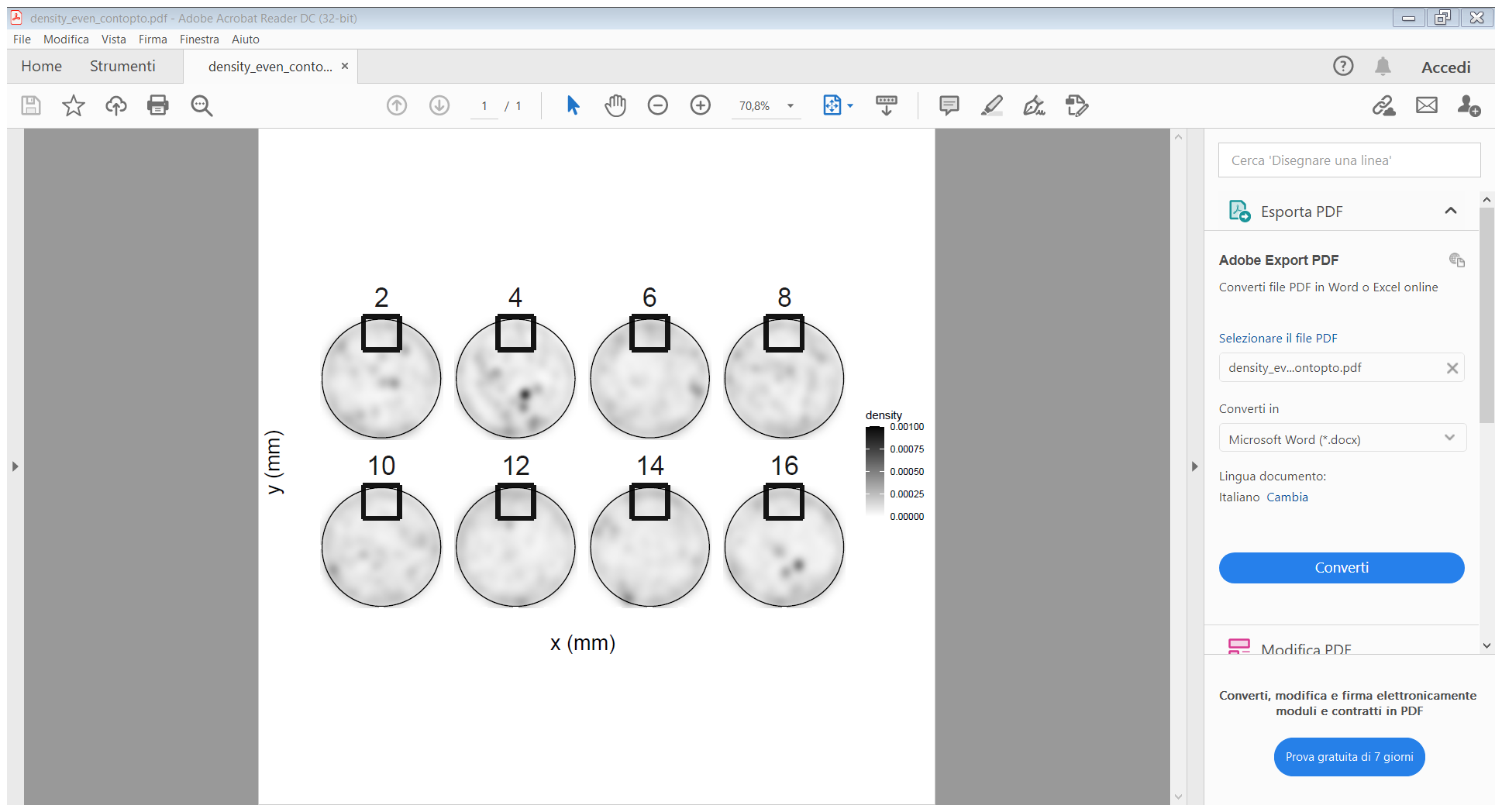

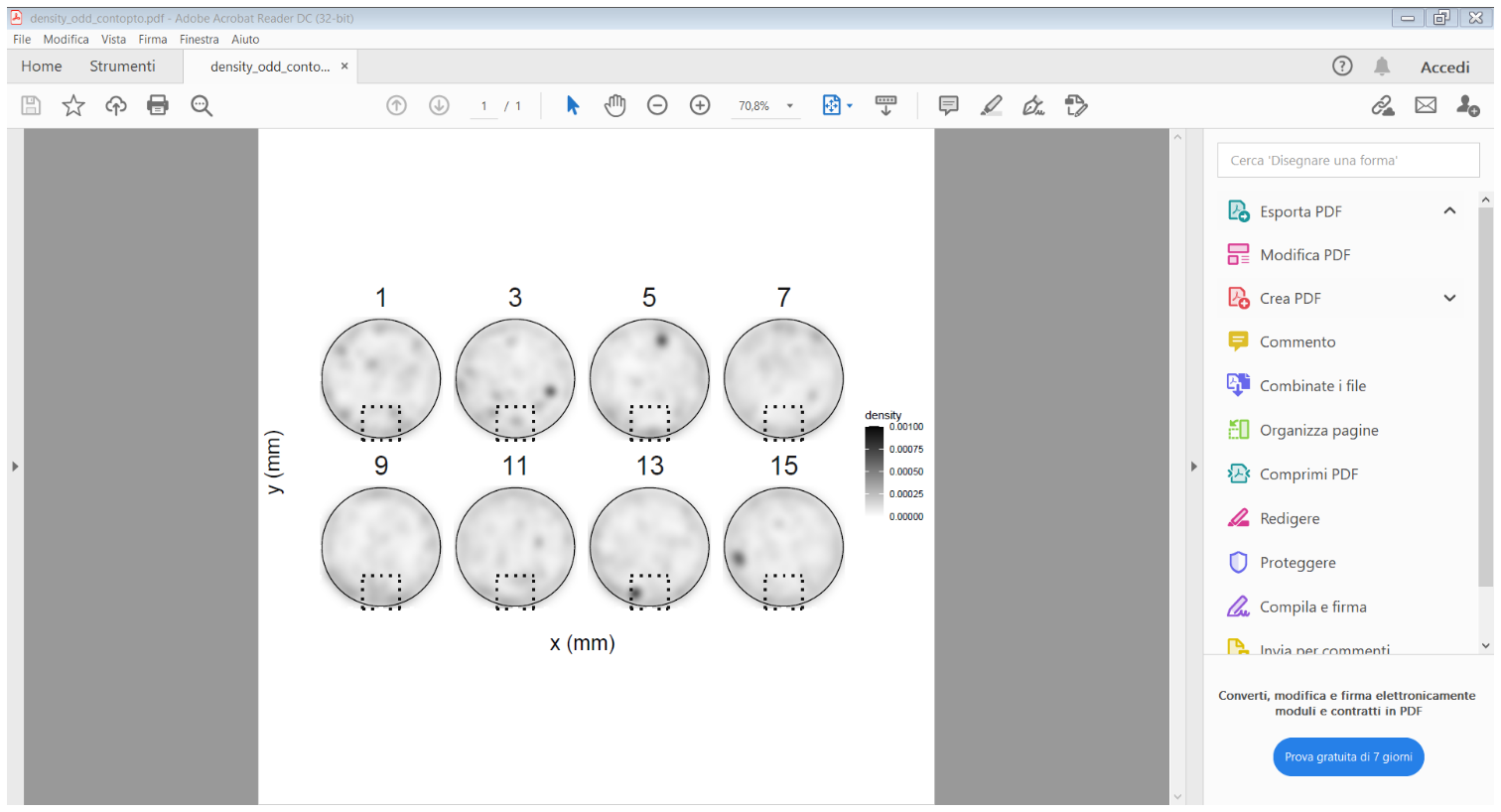

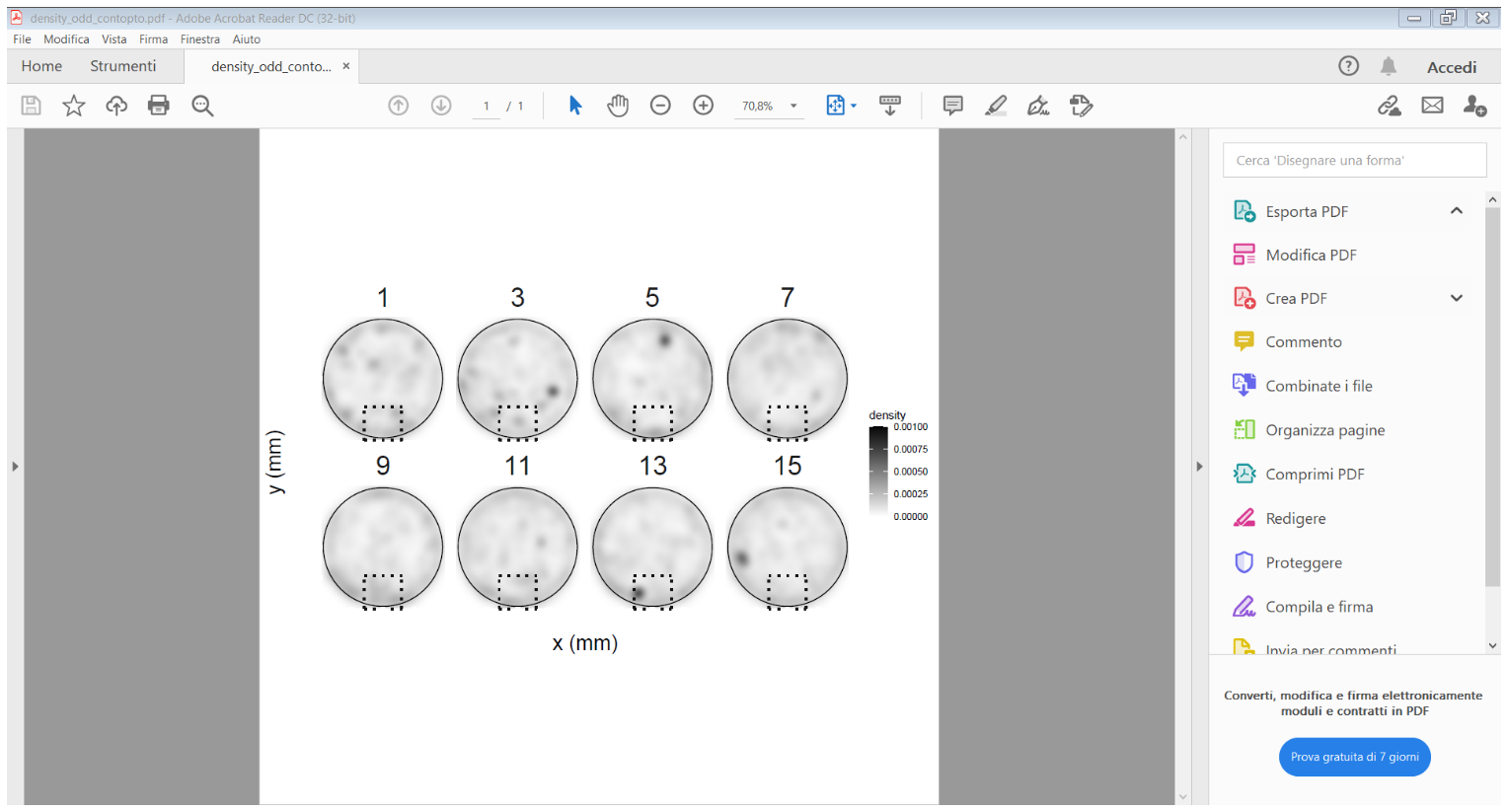


Trial 13-16

Trial 9-12

Trial 1-4

Trial 5-8


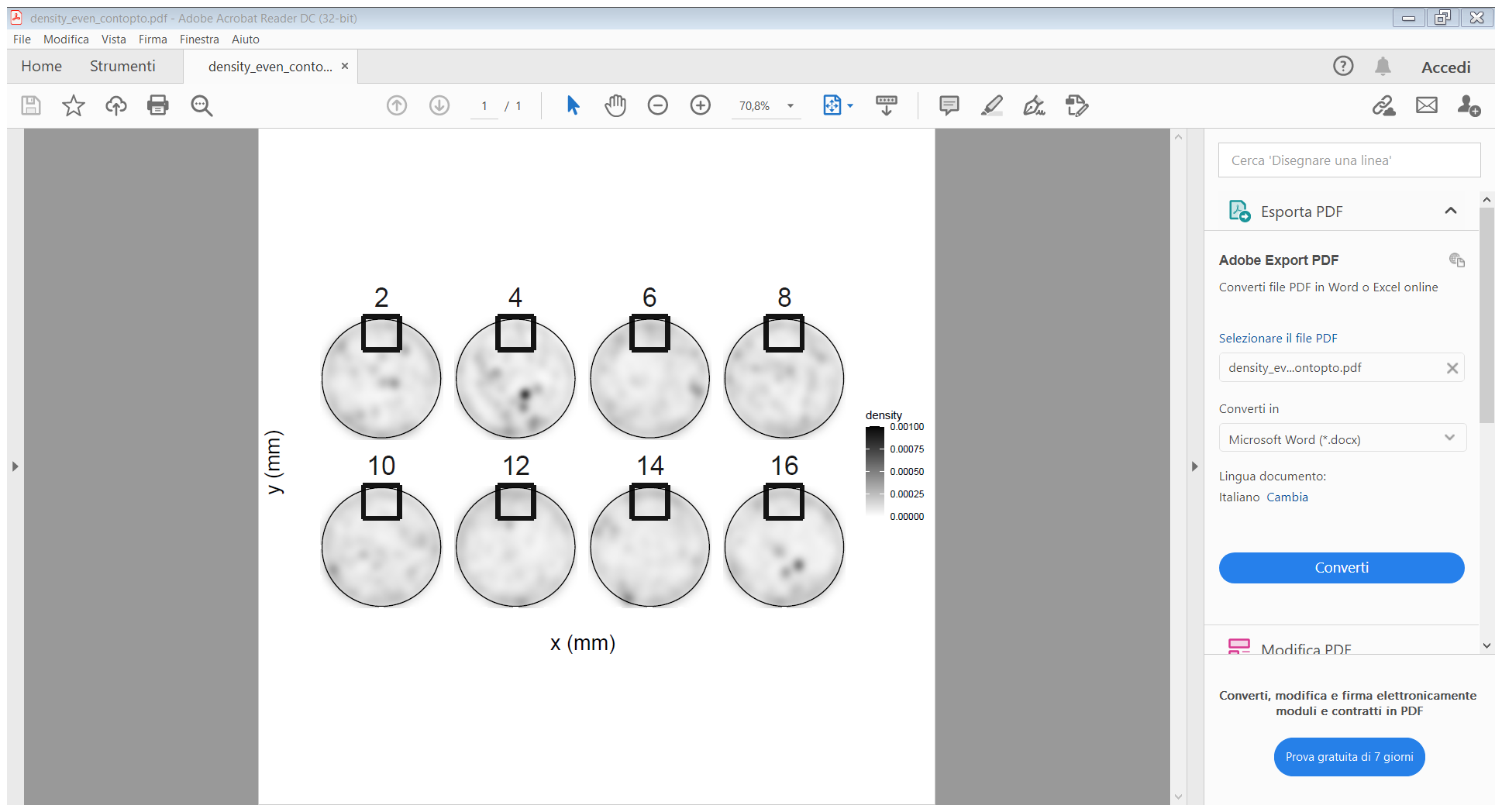

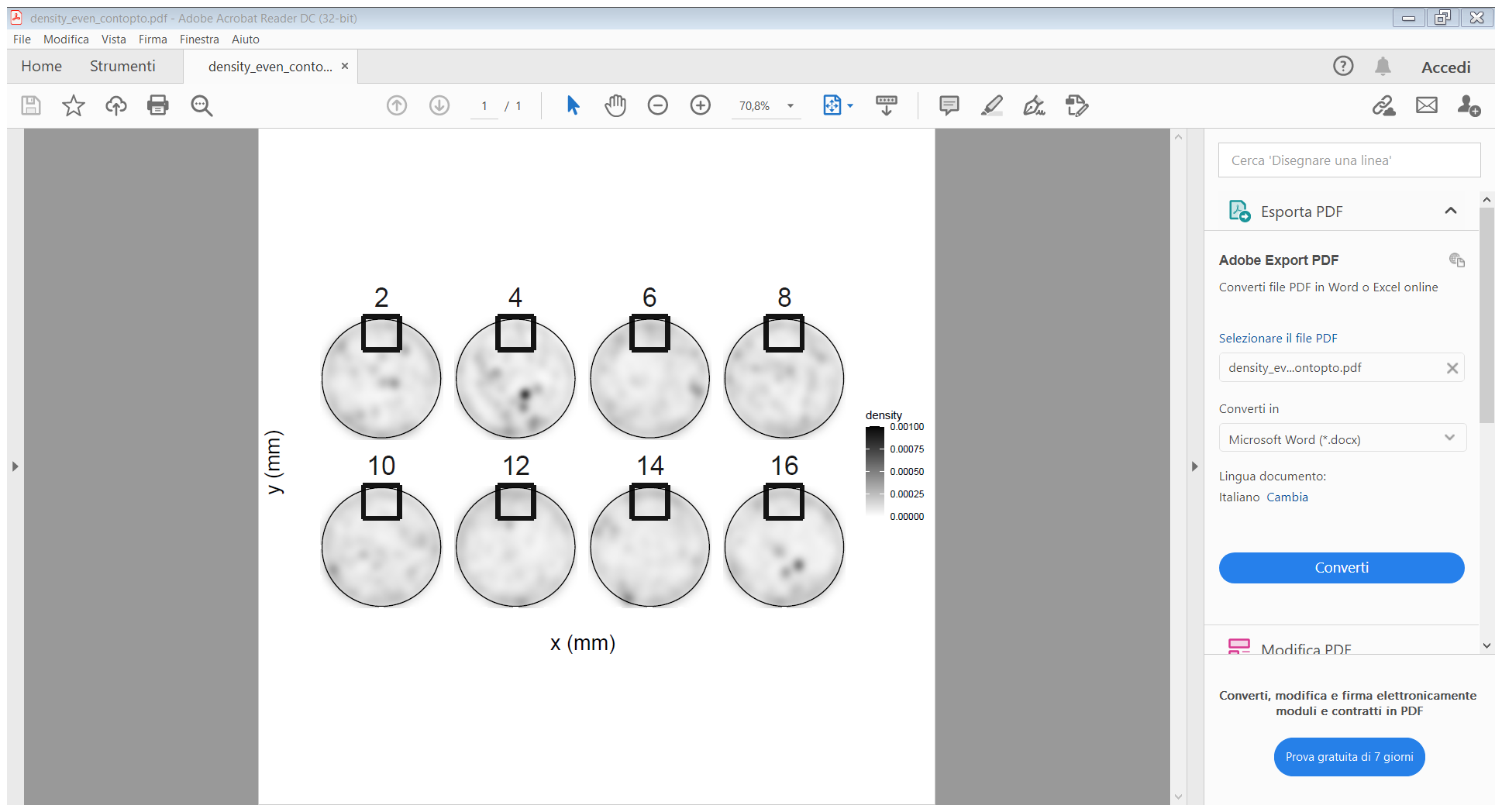

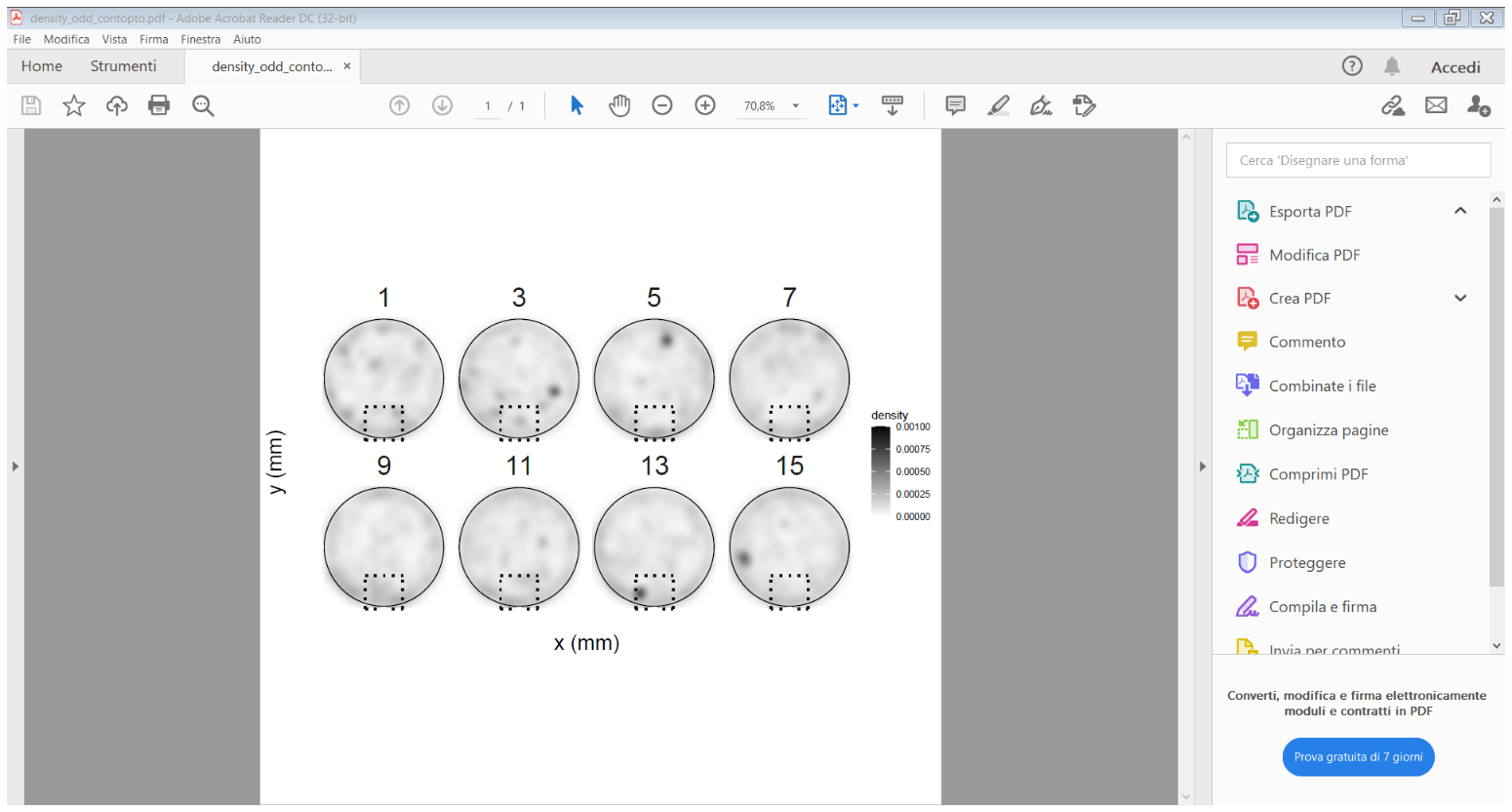

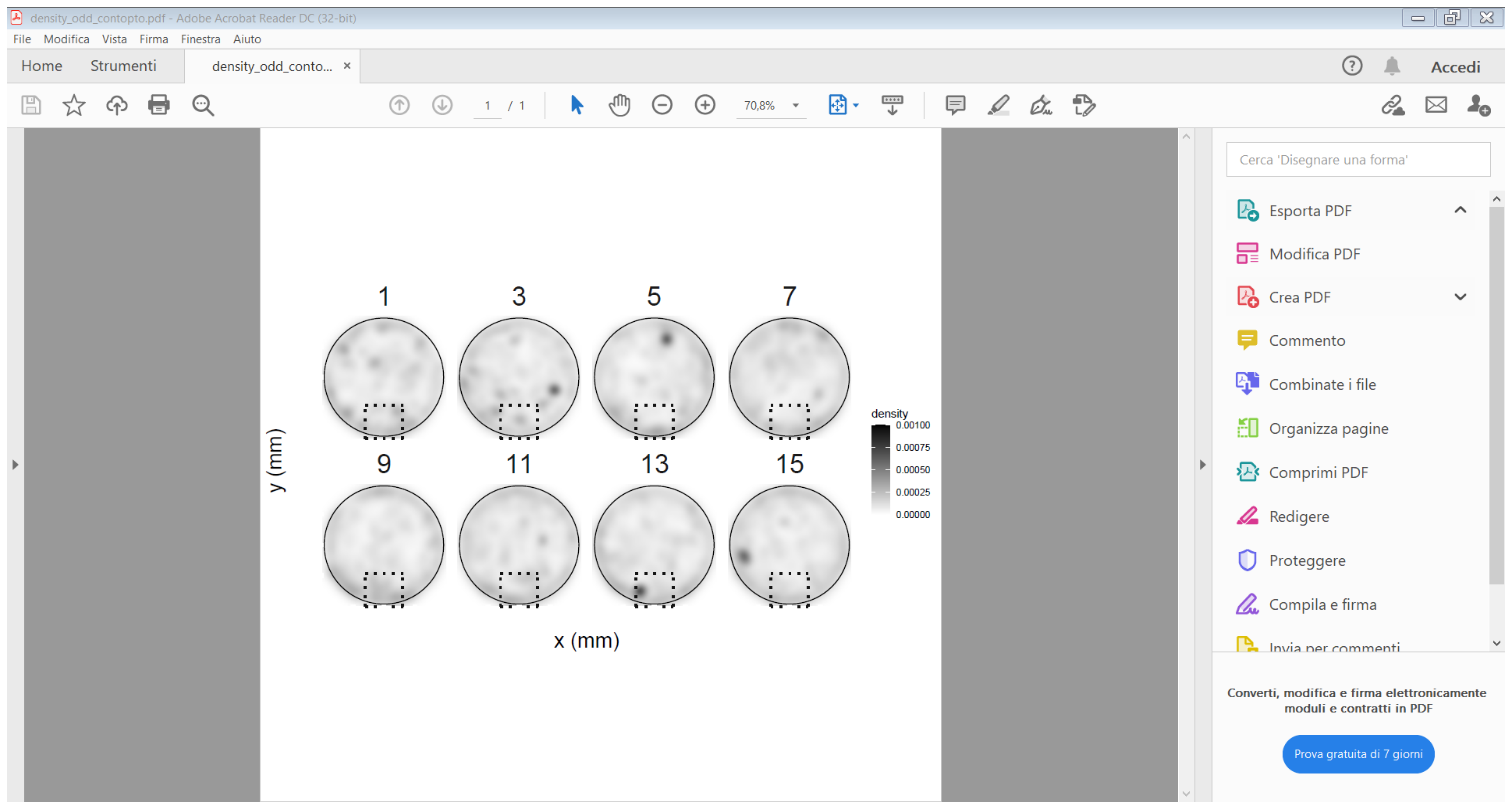


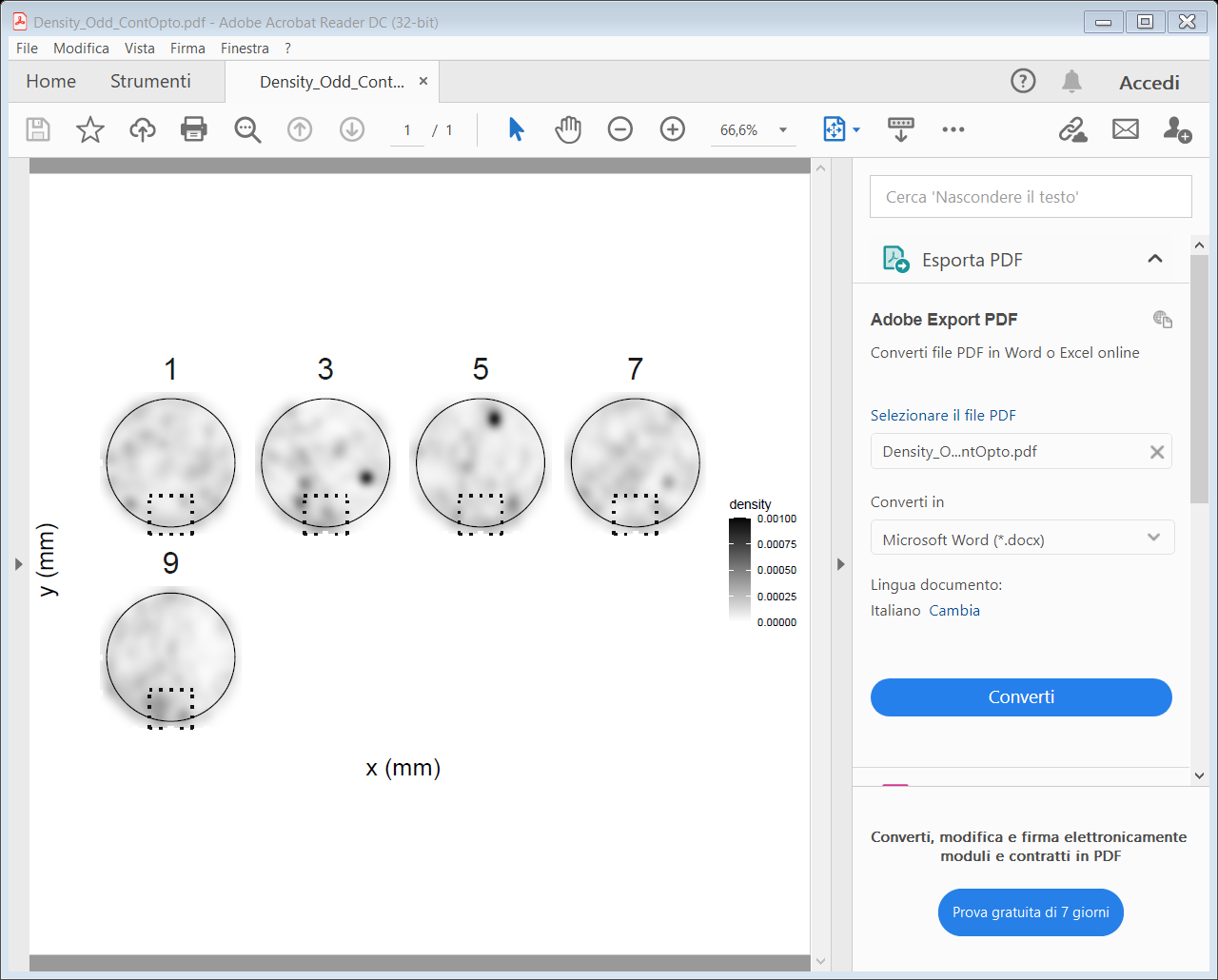

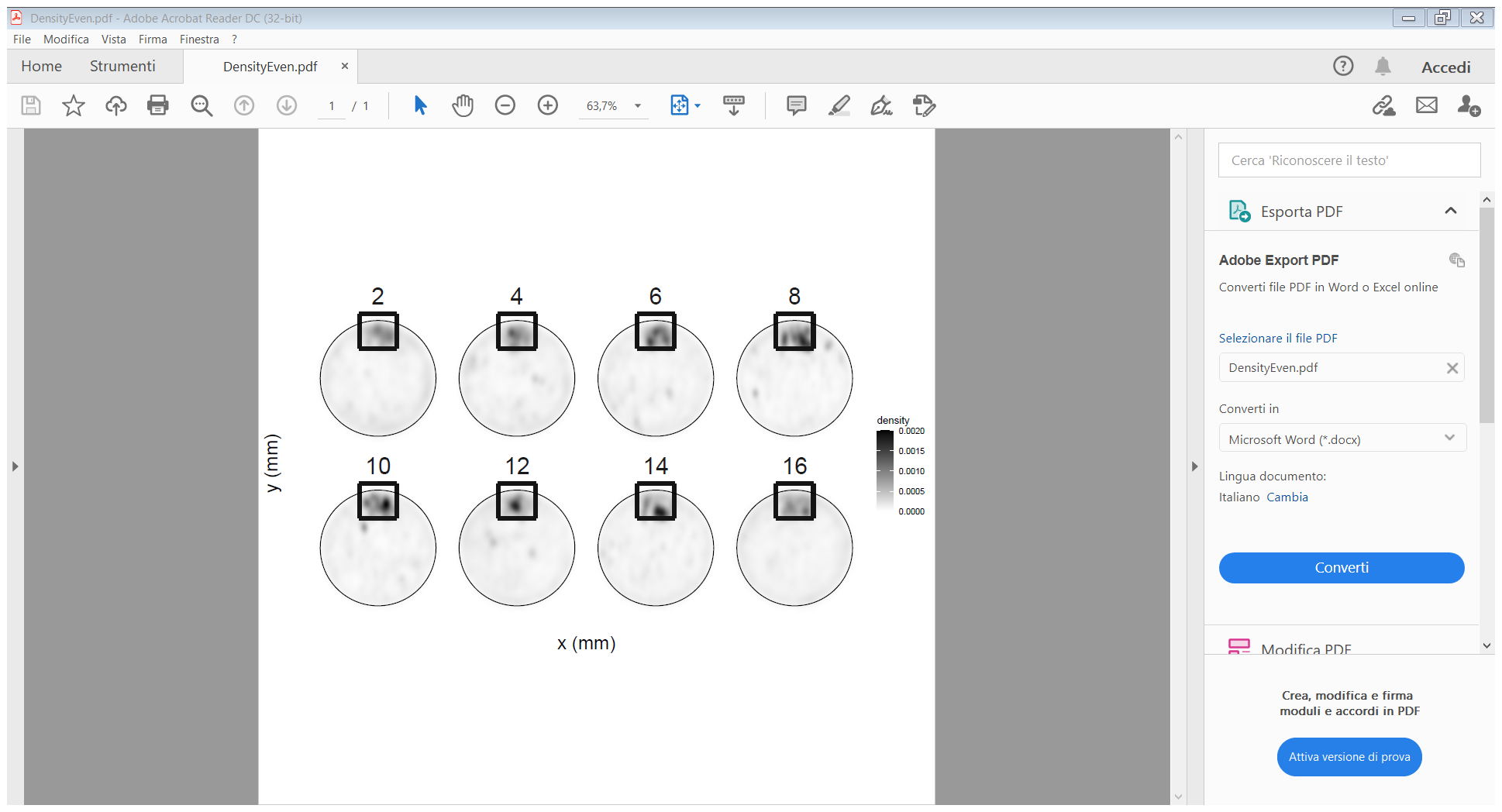


residency


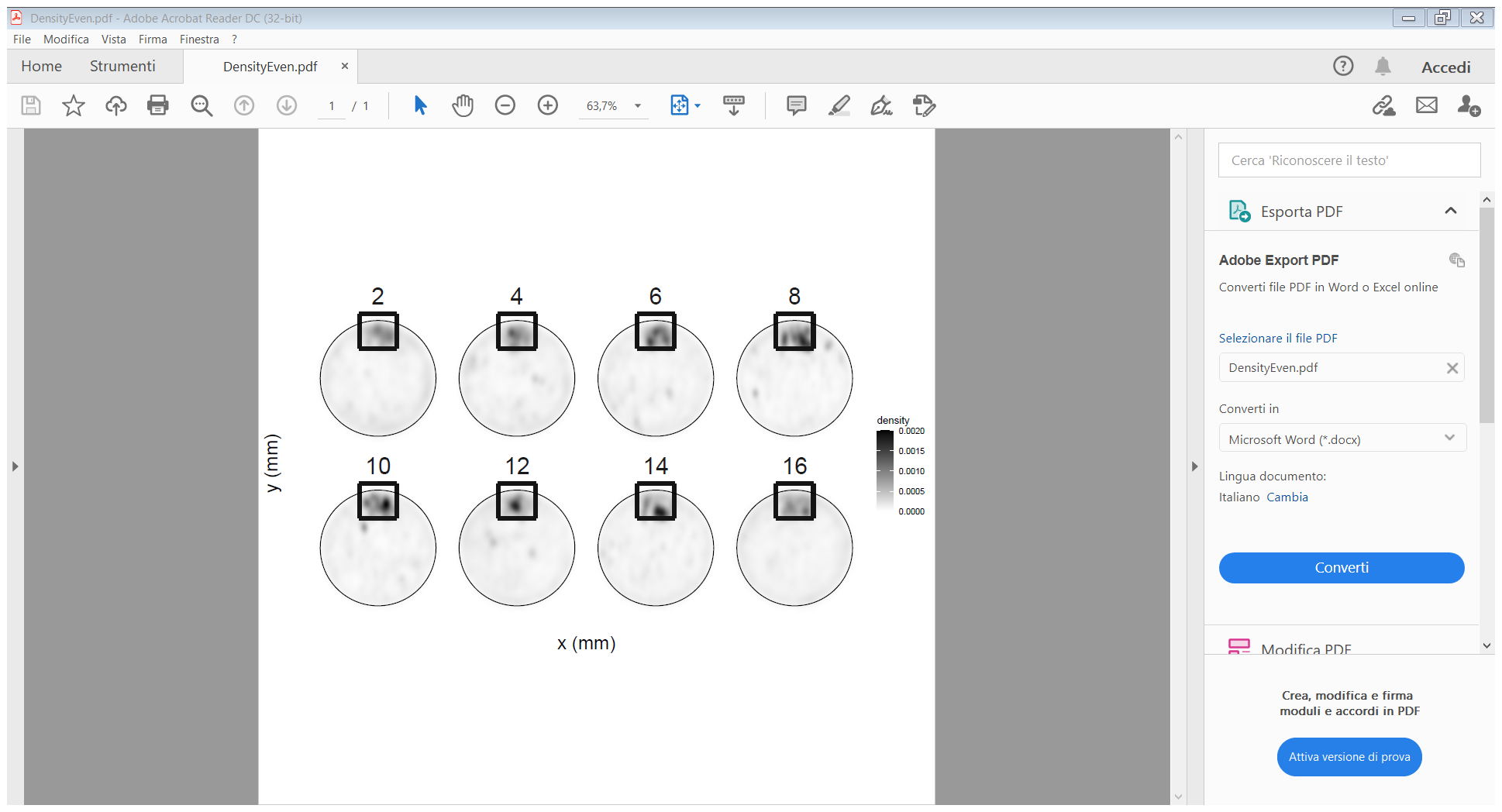

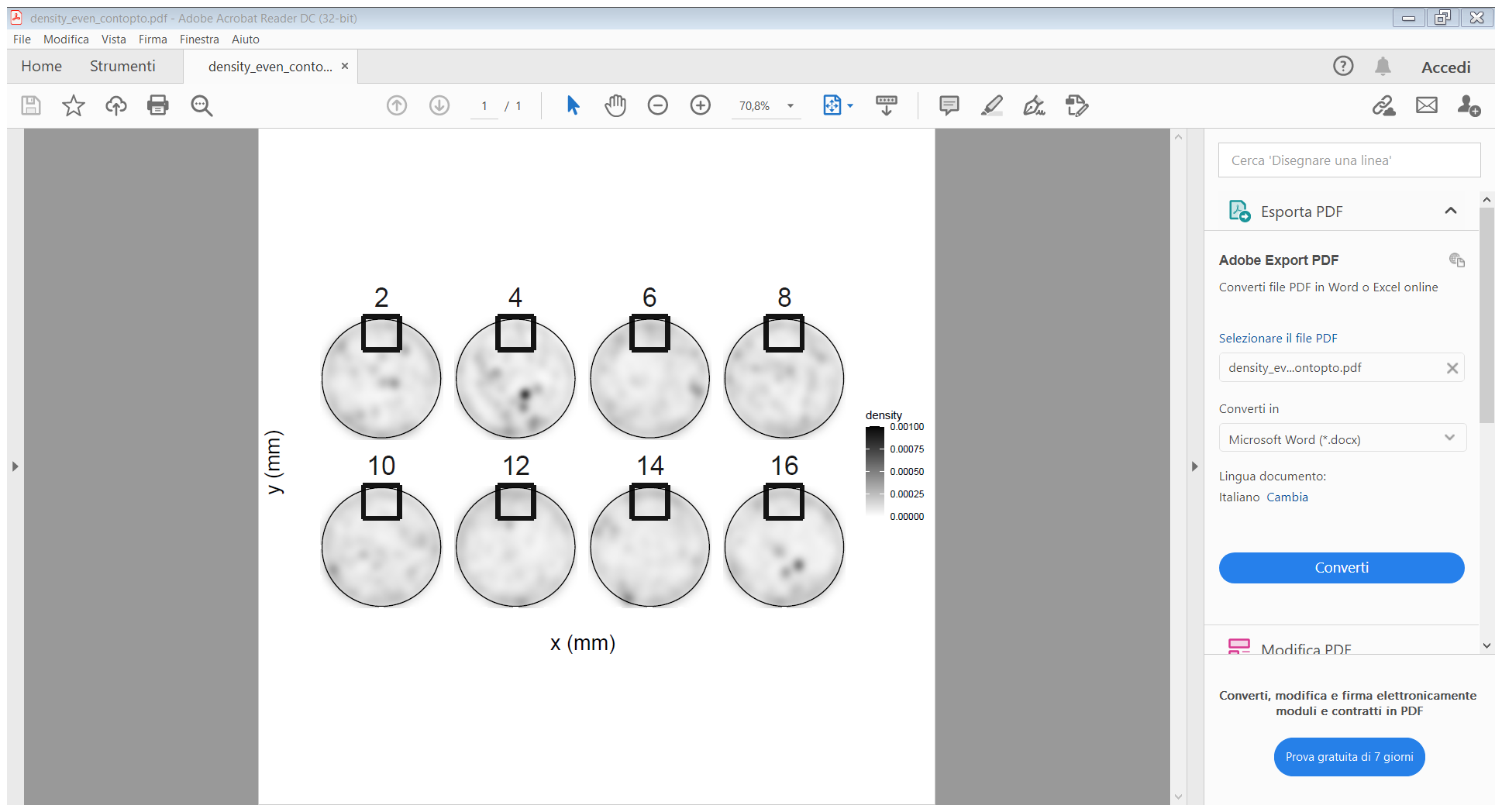

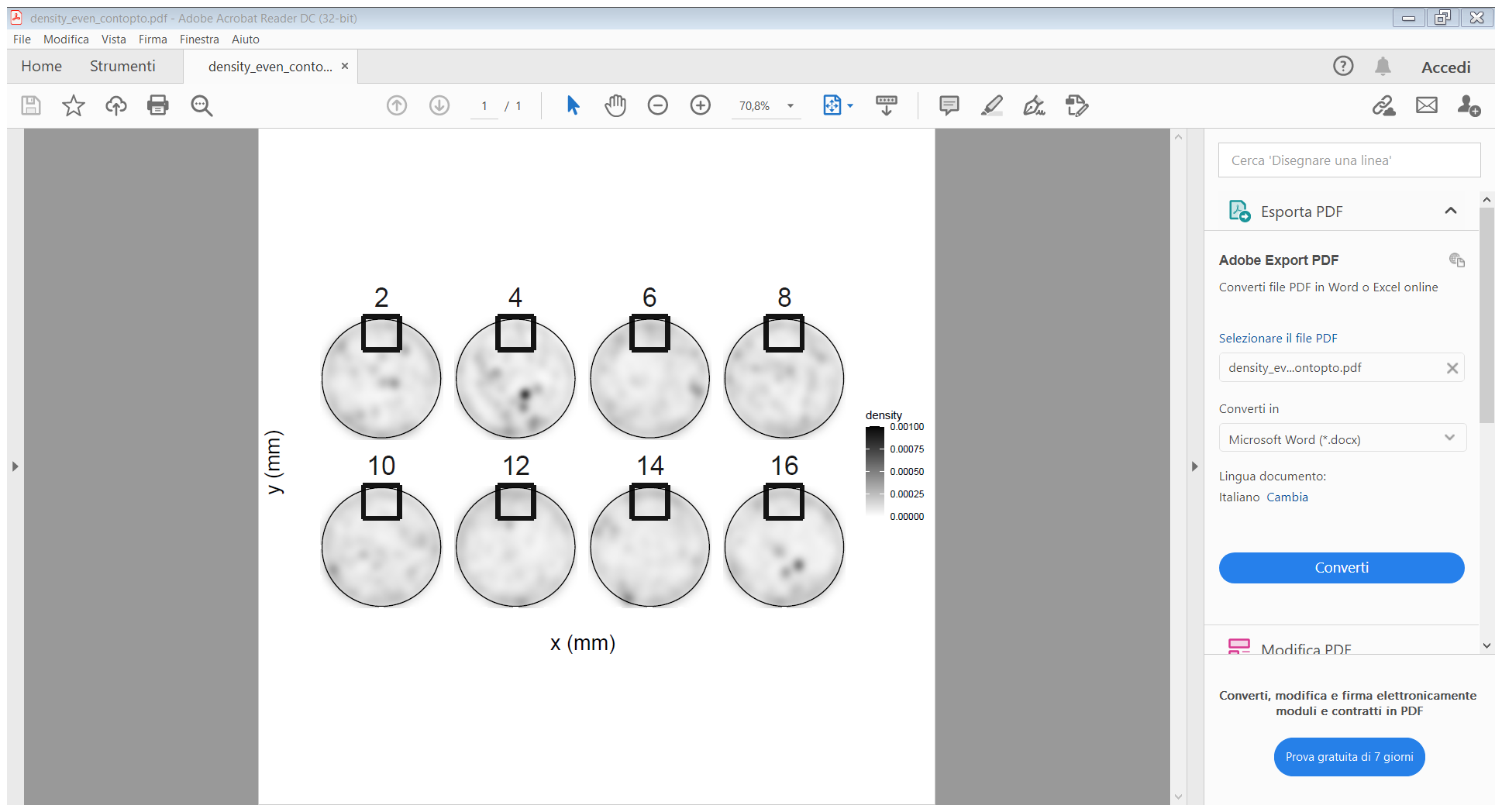

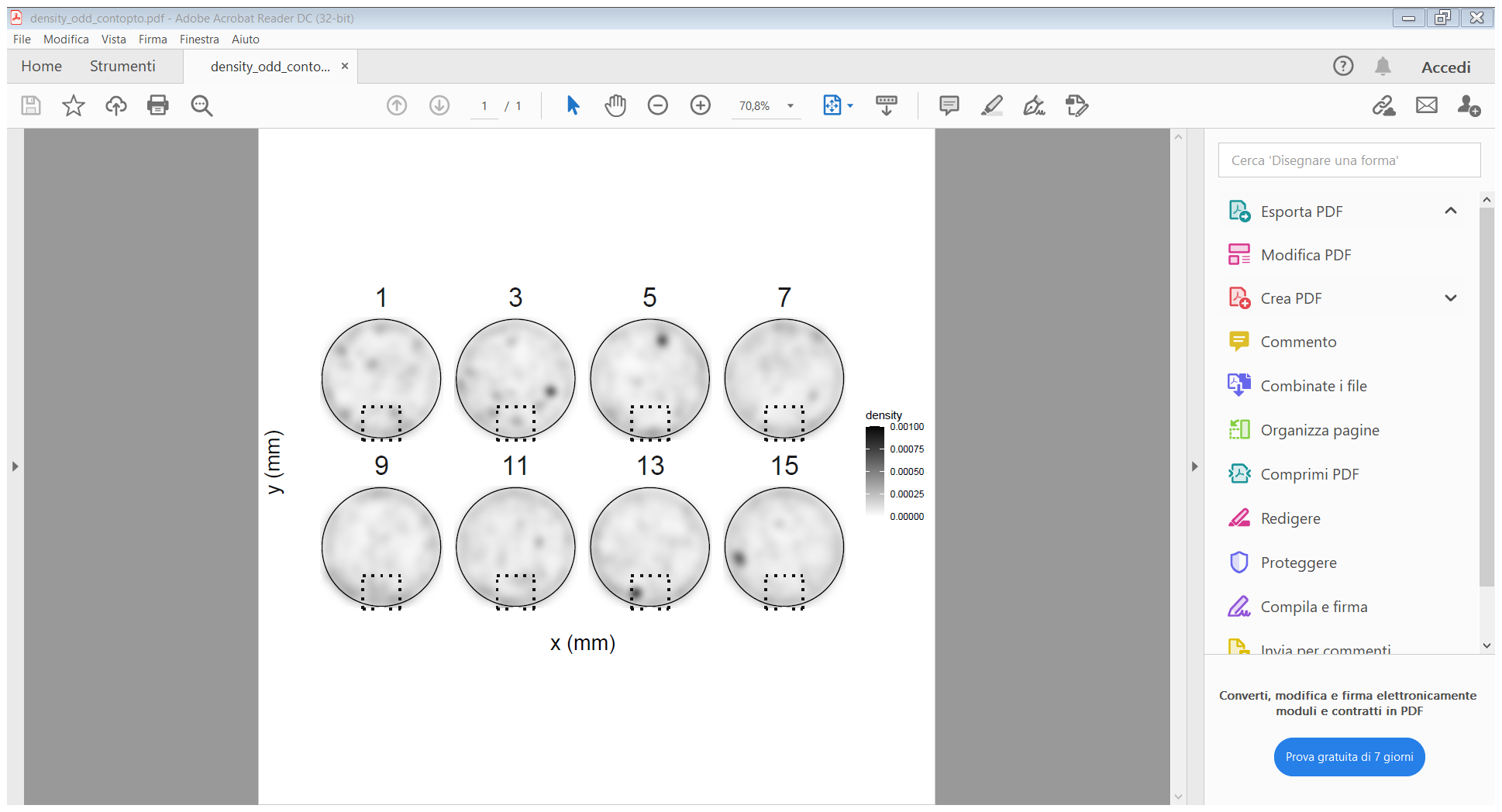

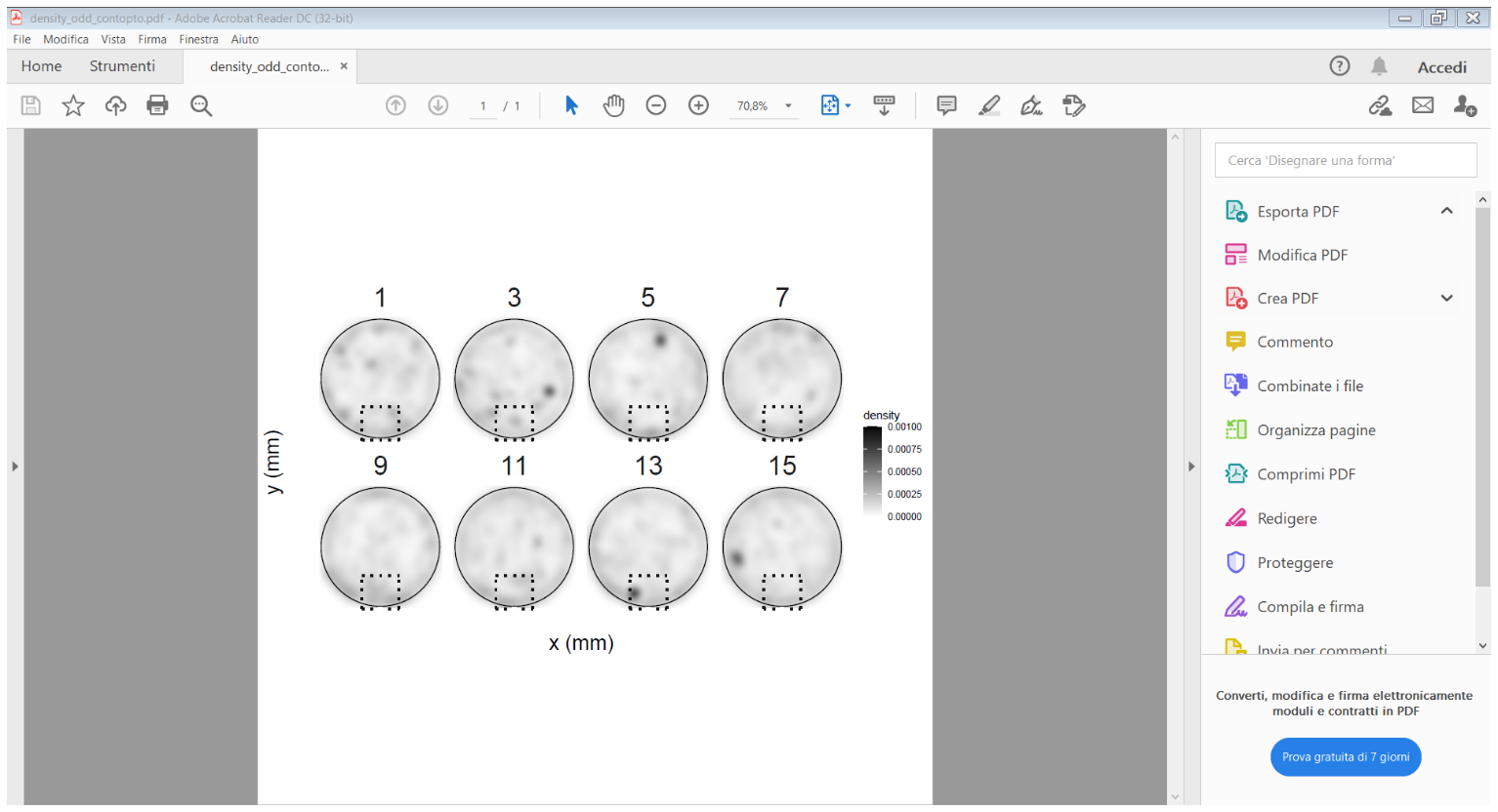


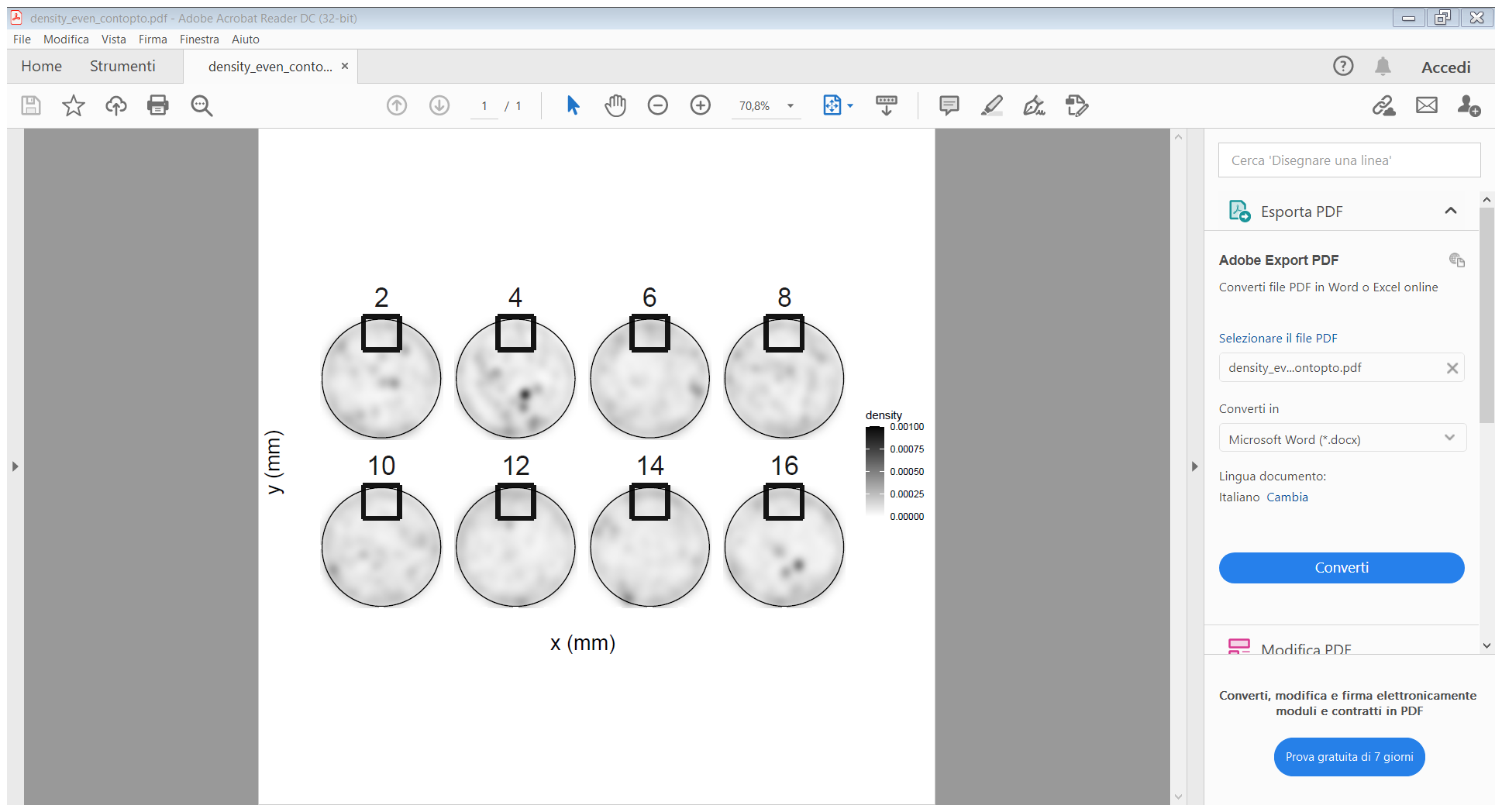

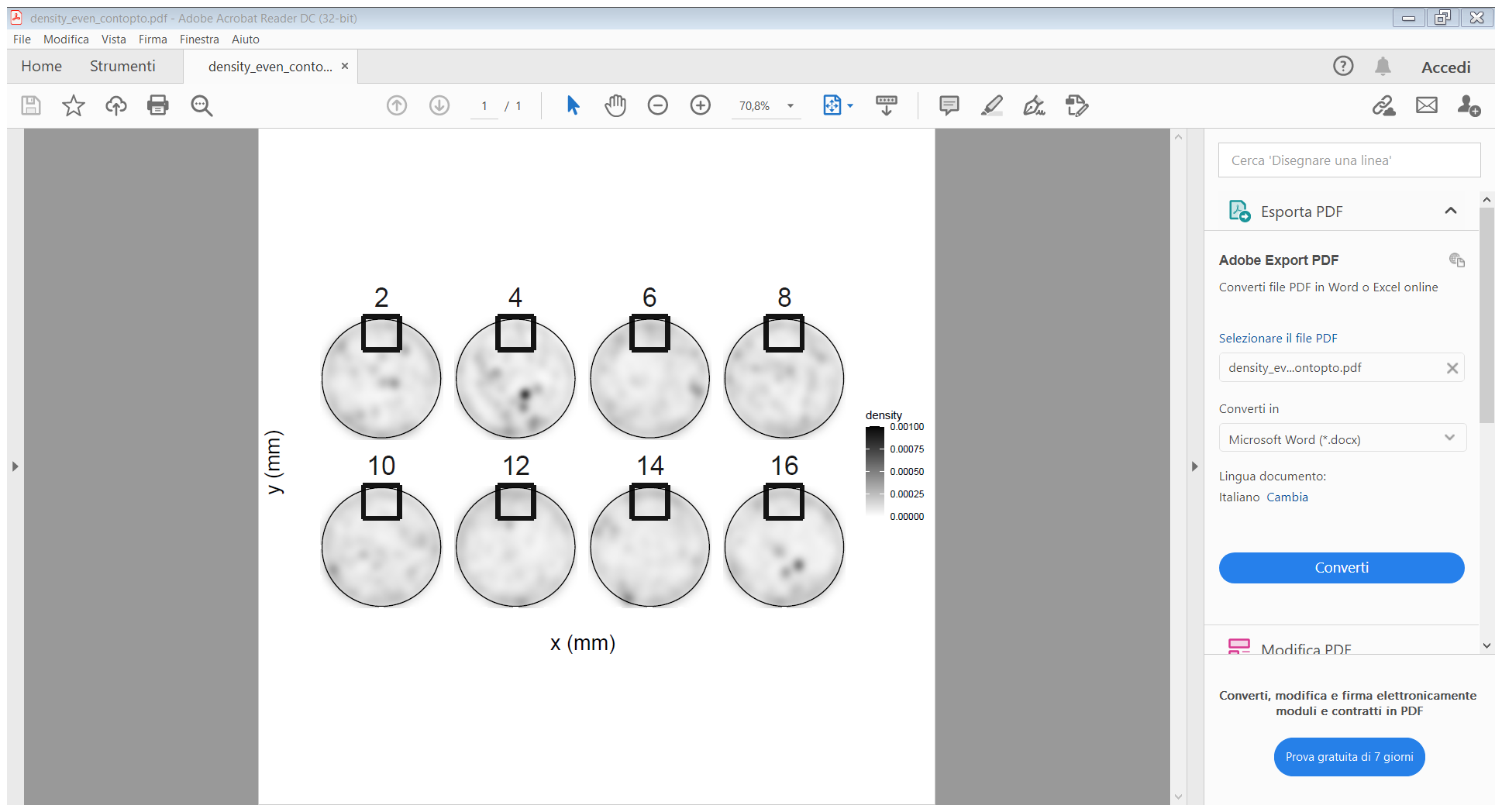

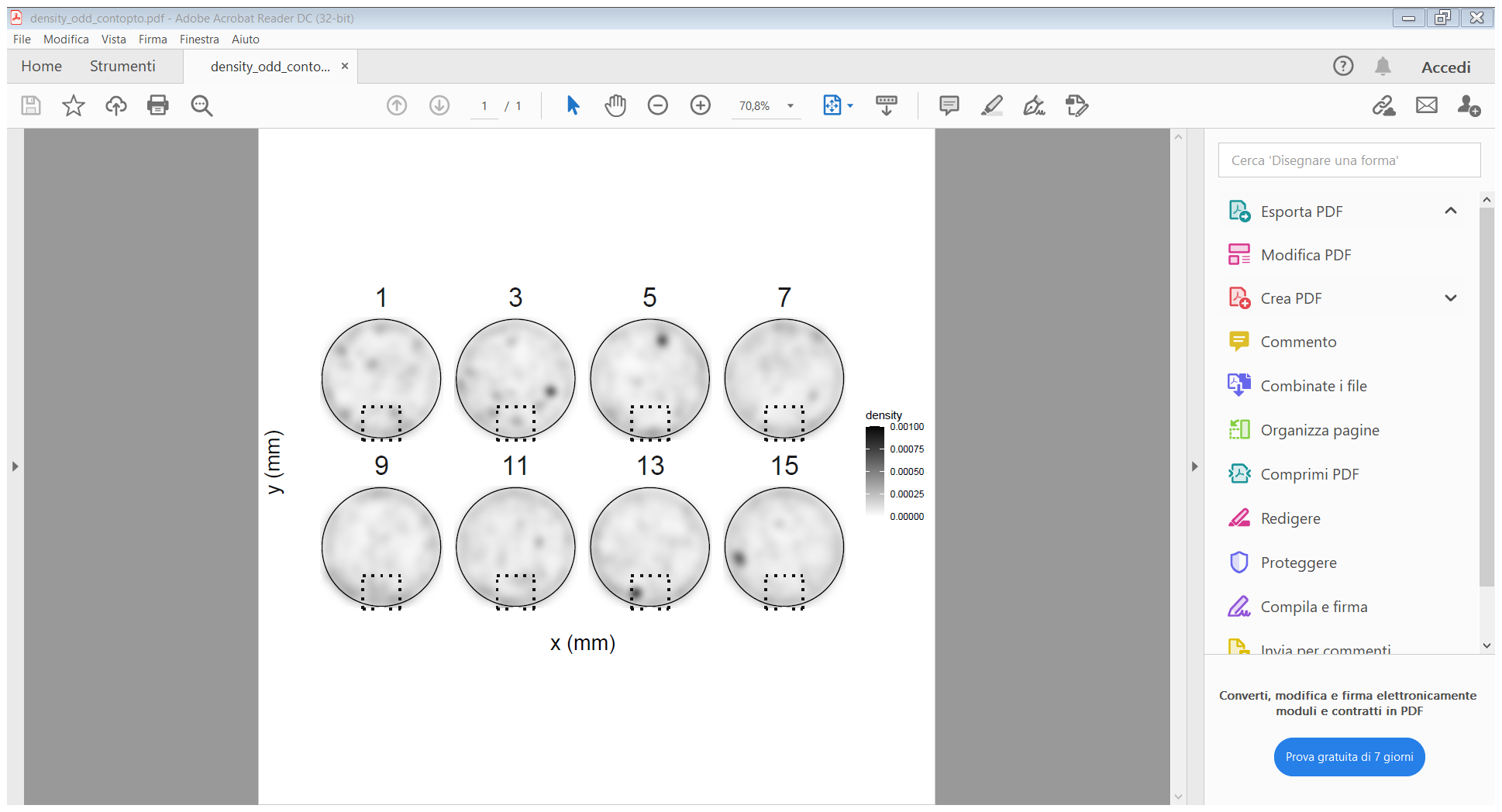

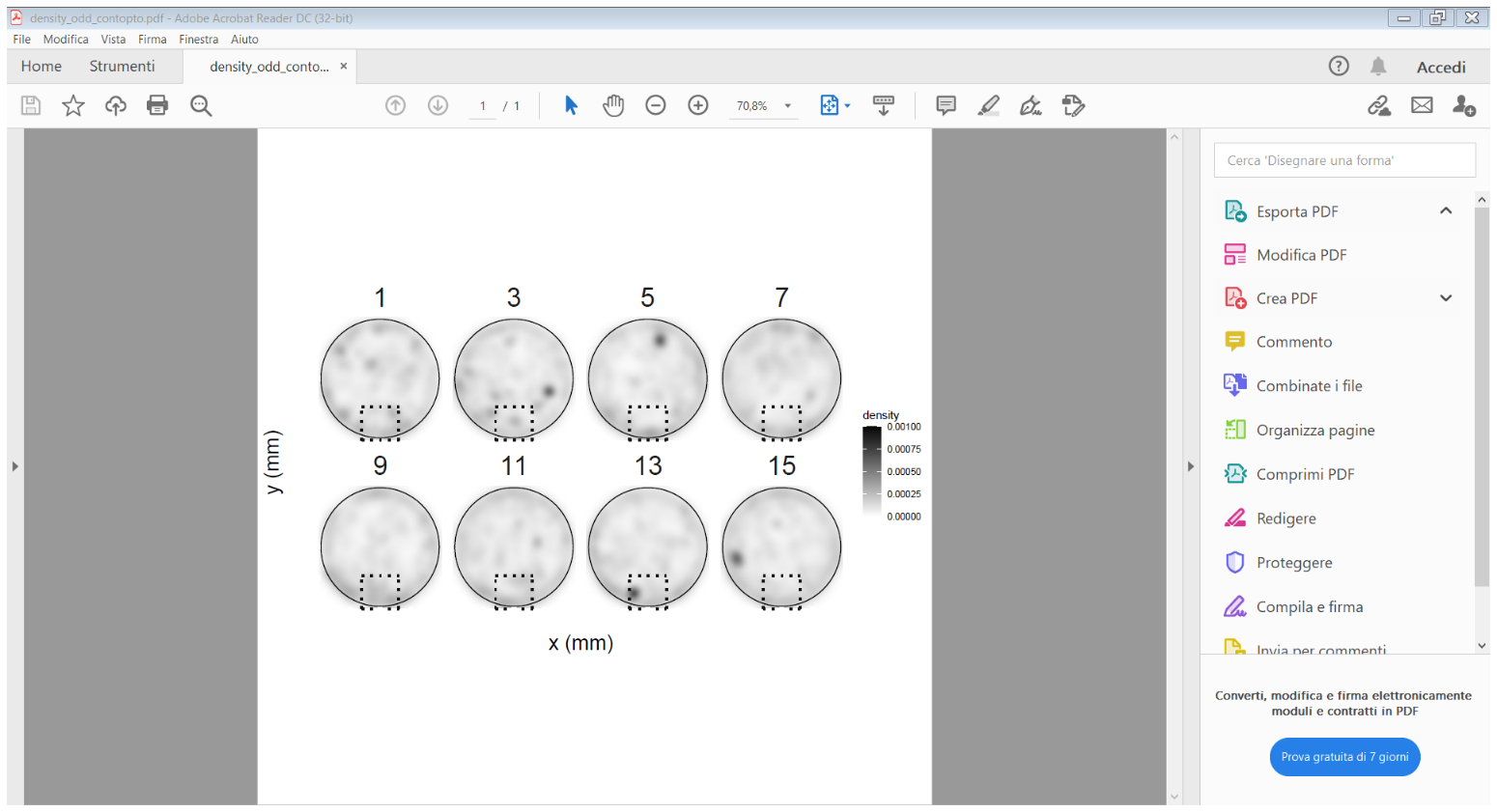


**Supplementary Figure S2. Gr66a-Gal4 > CsChrimson flies that cannot achieve relief do not «naturally» search in proximity to a specific landmark.**

Black bars = represent the vertical or horizontal stripe; red bolt = the fly is being negatively stimulated;

**A)** Data presented in this figure are related to a subgroup of flies reared on retinal-enriched food and expressing red-shifted rhodopsins in bitter-sensing neurons (n = 20). In this experiment, the flies could not achieve relief in any location of the arena for 16 trials (i.e., each trial resembled a probe session). This experiment was conducted to test if, during the negative stimulation, the fruit flies had an innate preference for one of the two stripes or they developed a preference as the number of trial progressed;

**B)** Performance Index (PI) as a function of training trials. Pointrange = mean ± confidence interval around the mean. PI was not computed for trial 1 given the absence of a Previous Safe Zone during that trial. Flies did not show a significant preference for one of the markers during the first 16 trials (see also Table S2); **C)** Velocity profile of fruit flies after entering a zone where light stimulation was shut off (black line) compared to the profile (grey line) after entering a zone where light stimulation was continued. Gray shadow represents 95% CI. No differences in the velocities could be observed (no. observed velocities = 4247, mean difference = 0.08, std error = 0.09, z.ratio = 0.89, p = 0.37) **D)** density plots describing the residency of flies during each of the 16 trials. **E)** During the 17^th^ trial, fruit flies spent an equal amount of time close to the vertical and to the horizontal bar (no. of comparisons = 37, mean difference in time spent (s) = 1.39, std error = 2.18, t.ratio = 0.63, p = 0.53), further showing that this group of flies did not have an innate attraction to the vertical bar or horizontal bar during the optogenetic stimulation.

|  | **Performance index** |  | **Performance index** |
| --- | --- | --- | --- |
| *Predictors* | *Estimates, mean (CI 95%)* | *Predictors* | *Estimates, mean (CI 95%)* |
| (Intercept) | -0.05  (-0.25 – 0.16) | ntrial [10] | -0.04  (-0.33 – 0.24) |
| ntrial [3] | 0.10  (-0.19 – 0.38) | ntrial [11] | 0.06  (-0.23 – 0.34) |
| ntrial [4] | -0.26  (-0.55 – 0.02) | ntrial [12] | 0.14  (-0.14 – 0.42) |
| ntrial [5] | 0.08  (-0.21 – 0.36) | ntrial [13] | 0.18  (-0.11 – 0.46) |
| ntrial [6] | 0.07  (-0.21 – 0.35) | ntrial [14] | 0.09  (-0.20 – 0.37) |
| ntrial [7] | 0.05  (-0.23 – 0.33) | ntrial [15] | 0.09  (-0.19 – 0.38) |
| ntrial [8] | -0.18  (-0.46 – 0.10) | ntrial [16] | -0.09  (-0.37 – 0.20) |
| ntrial [9] | 0.24  (-0.05 – 0.52) |  |  |
| Flies tested = 20 | | | |
| ** p<0.05   ** p<0.01   *** p<0.001* | | | |

**Table S2 related to Supplementary Figure S2B. Performance index for each trial.**

ntrial = trial number. CI 95% = 95% confidence interval. The estimates for the intercept and ntrial[3],…, ntrial[16] are the output of a linear mixed-effects model that describes the performance index as a function of trials, for each animal. PI was not computed for trial 1 given the absence of a Previous Safe Zone during that trial. The performance index during ntrial [2] is used as the reference value to compute the performance index estimates during the remaining ntrials.

|  | **Performance index** |  | **Performance index** |
| --- | --- | --- | --- |
| *Predictors* | *Estimates, mean (CI 95%)* | *Predictors* | *Estimates, mean (CI 95%)* |
| (Intercept) | 0.13  (-0.06 – 0.32) | ntrial [10] | 0.23  (-0.03 – 0.50) |
| ntrial [3] | 0.26  (-0.00 – 0.53) | ntrial [11] | 0.30 ^*^ (0.04 – 0.57) |
| ntrial [4] | 0.41 ^**^ (0.15 – 0.68) | ntrial [12] | 0.40 ^**^ (0.14 – 0.66) |
| ntrial [5] | 0.17  (-0.10 – 0.43) | ntrial [13] | 0.42 ^**^ (0.15 – 0.68) |
| ntrial [6] | 0.40 ^**^ (0.13 – 0.66) | ntrial [14] | 0.22  (-0.05 – 0.48) |
| ntrial [7] | 0.43 ^**^ (0.16 – 0.69) | ntrial [15] | 0.29 ^*^ (0.03 – 0.55) |
| ntrial [8] | 0.40 ^**^ (0.14 – 0.67) | ntrial [16] | 0.35 ^**^ (0.09 – 0.61) |
| ntrial [9] | 0.29 ^*^ (0.03 – 0.56) |  |  |
| Flies tested = 40 | | | |
| ** p<0.05   ** p<0.01   *** p<0.001* | | | |

**Table S3 related to (main text) Figure 1B. Performance index for each trial.**

ntrial = trial number. CI 95% = 95% confidence interval. The estimates for the intercept and ntrial[3],…, ntrial[16] are the output of a linear mixed-effects model that describes the performance index as a function of trials, for each animal. PI was not computed for trial 1 given the absence of a Previous Safe Zone during that trial. The performance index during ntrial [2] is used as the reference value to compute the performance index estimates during the remaining ntrials.


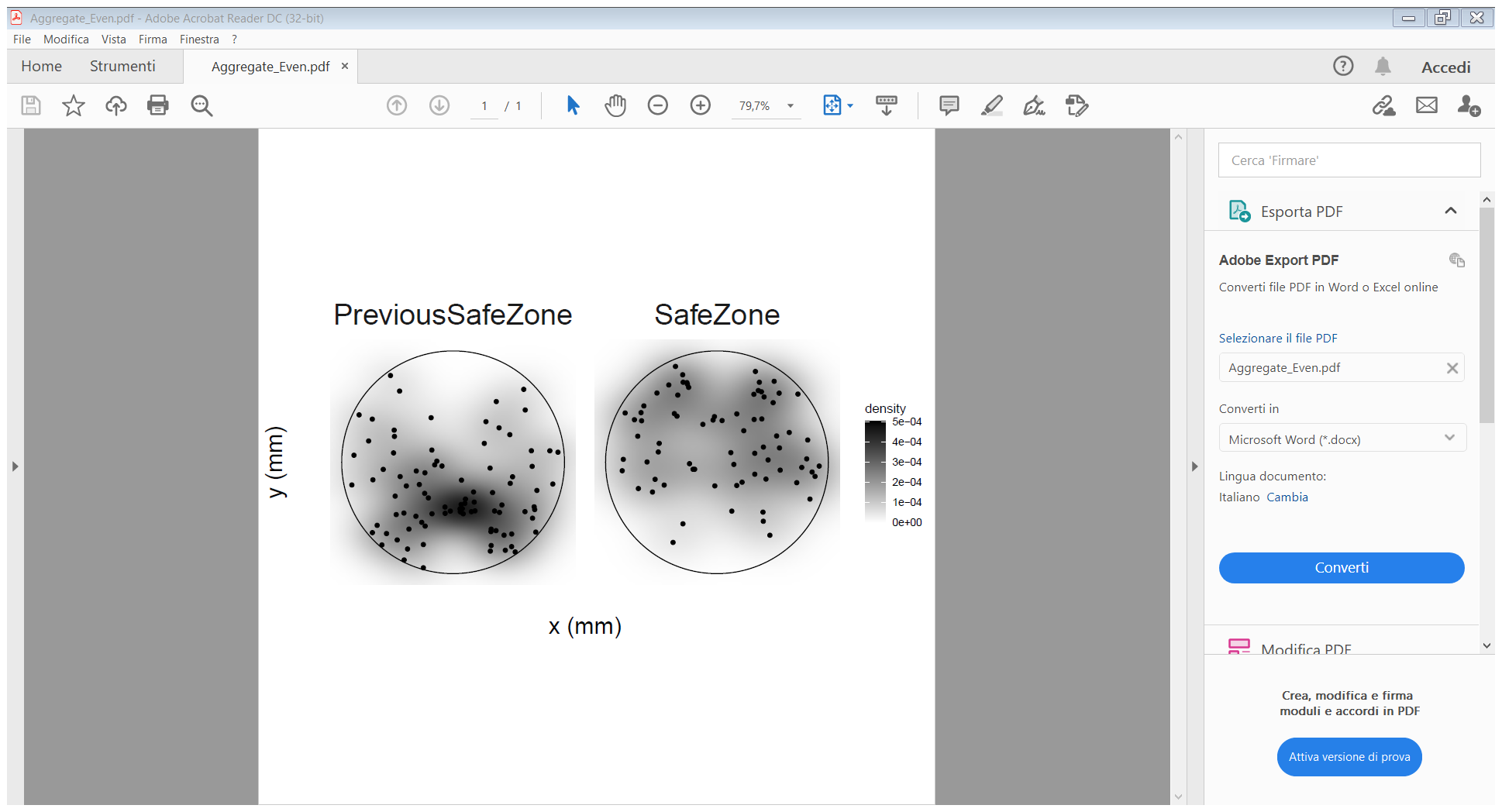

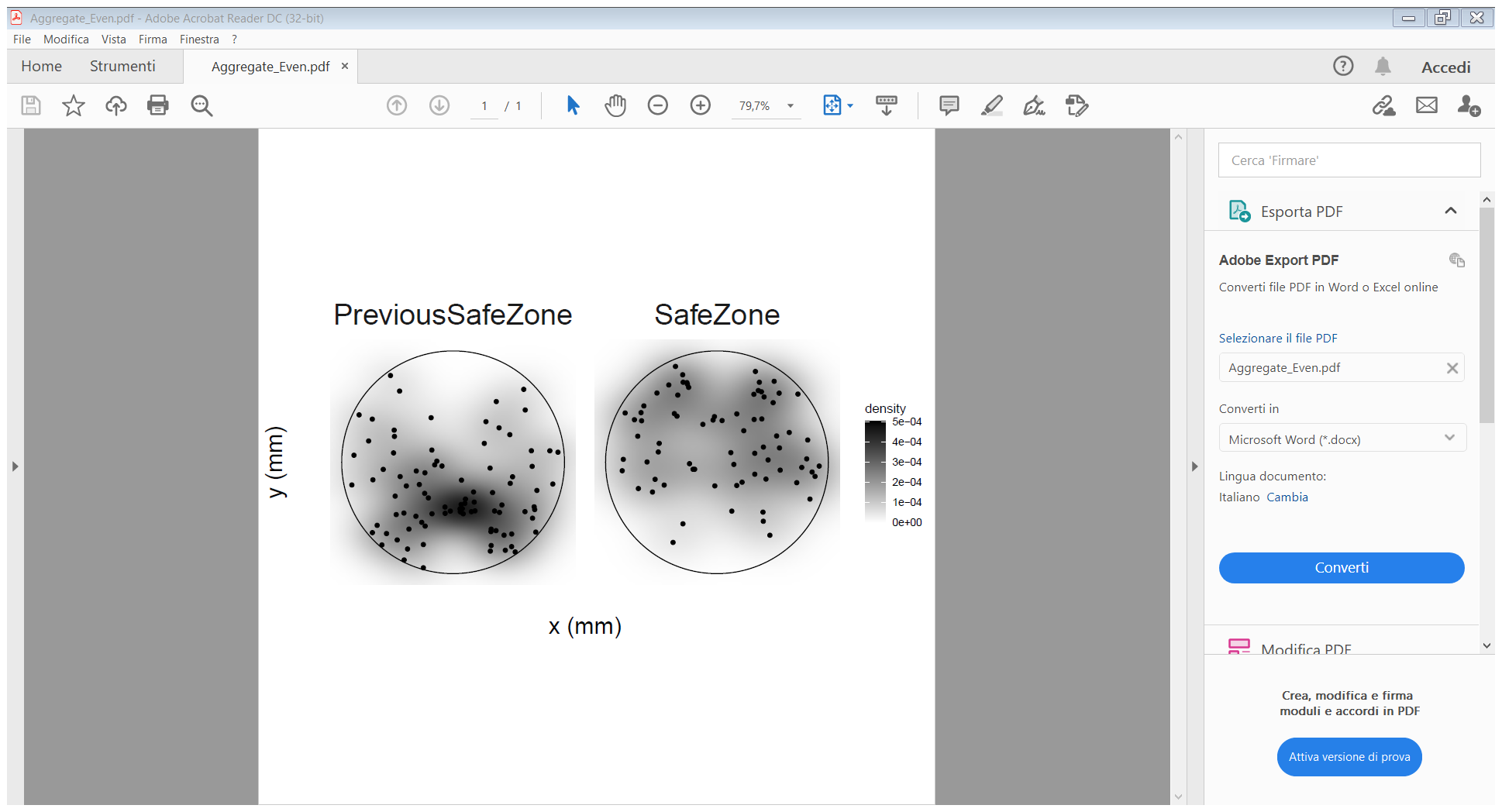

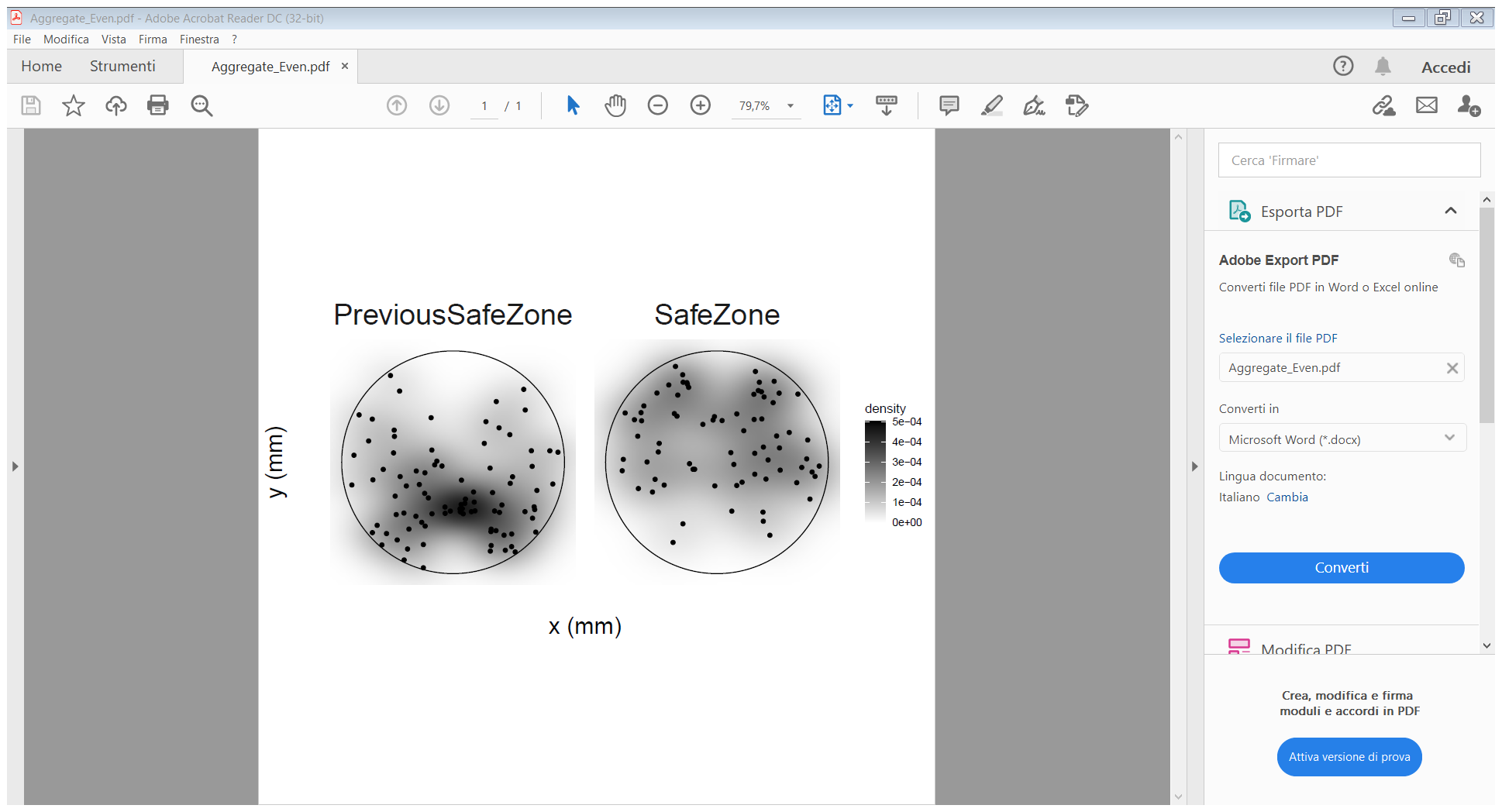

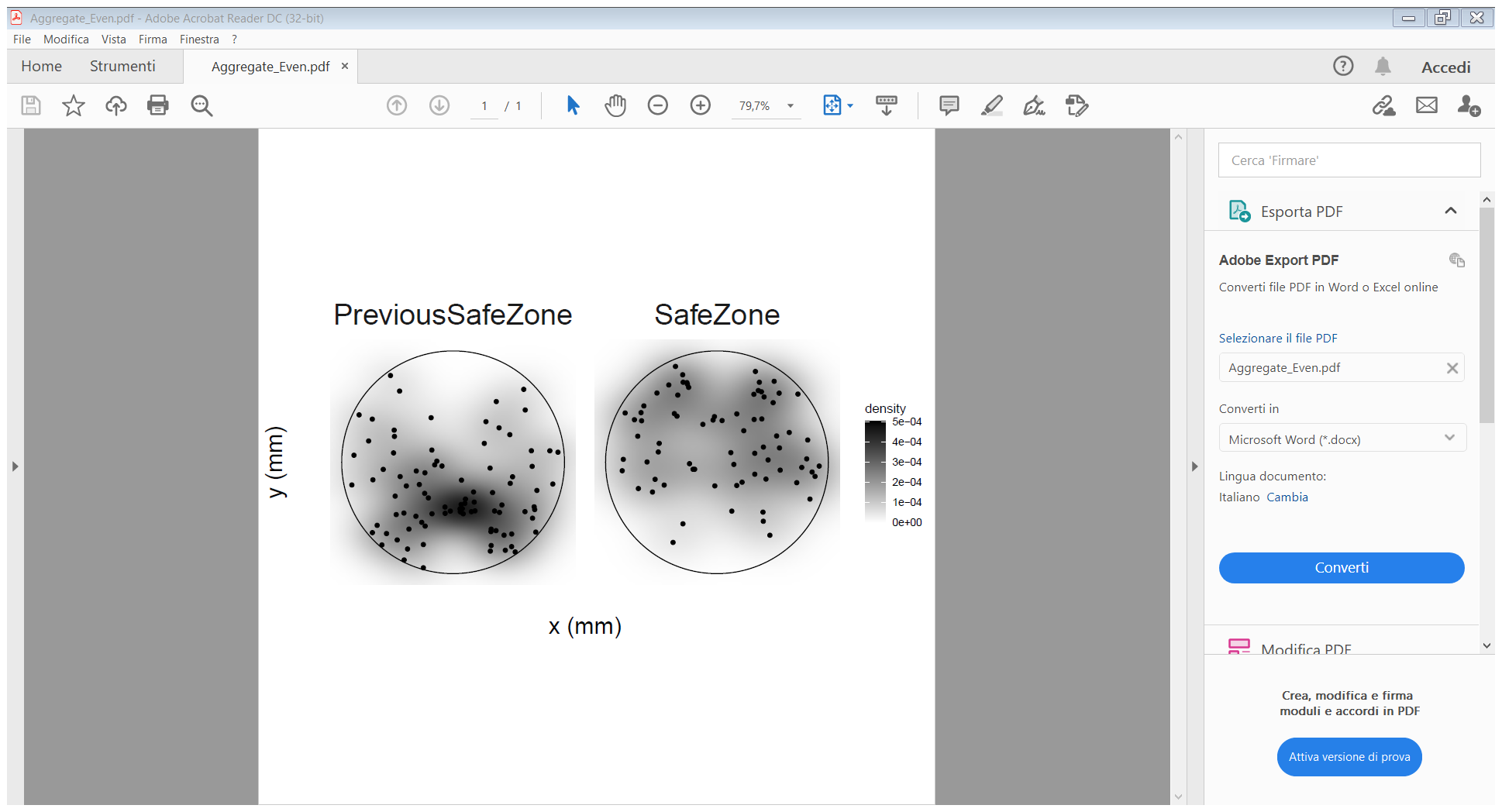

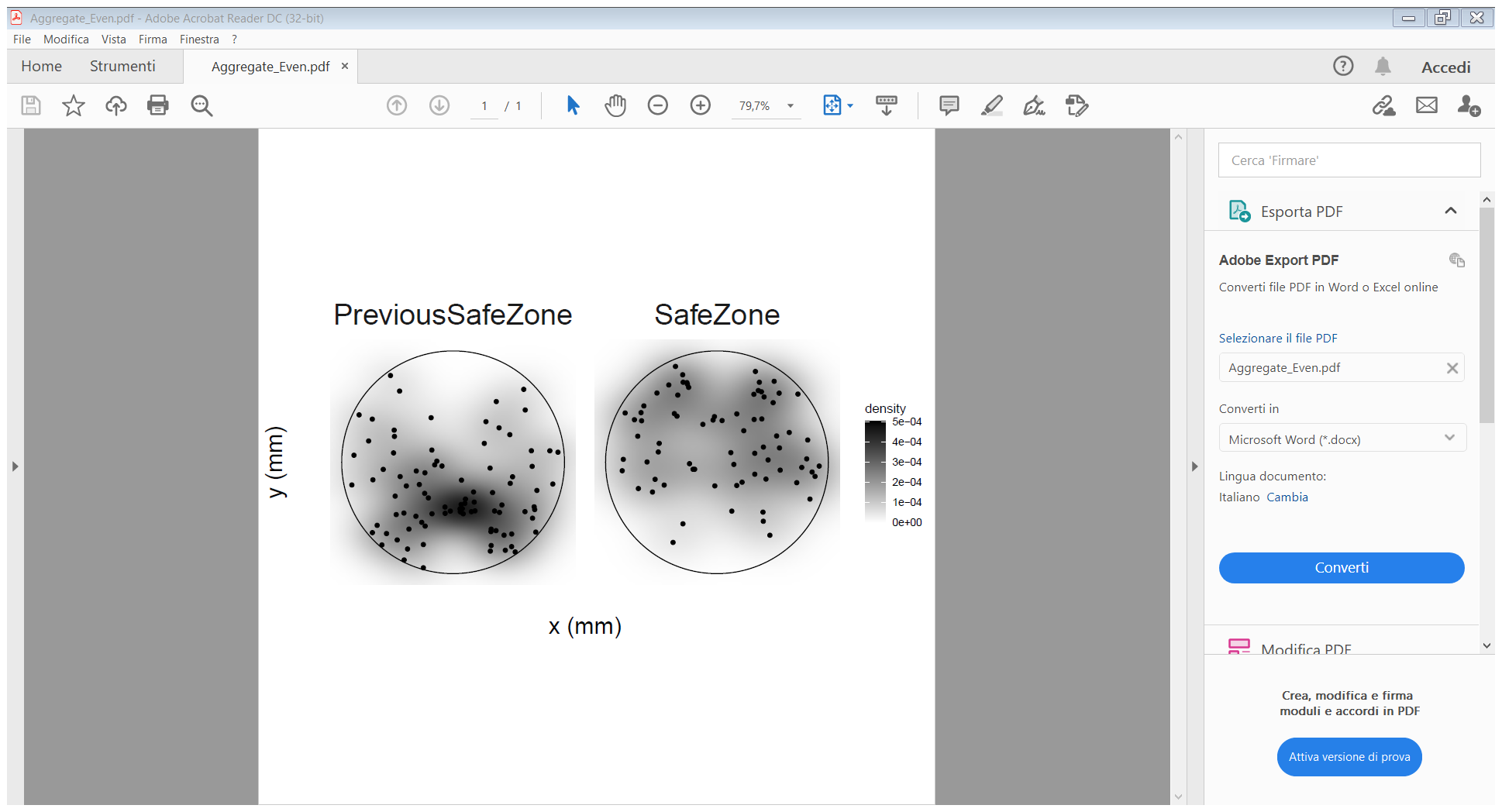

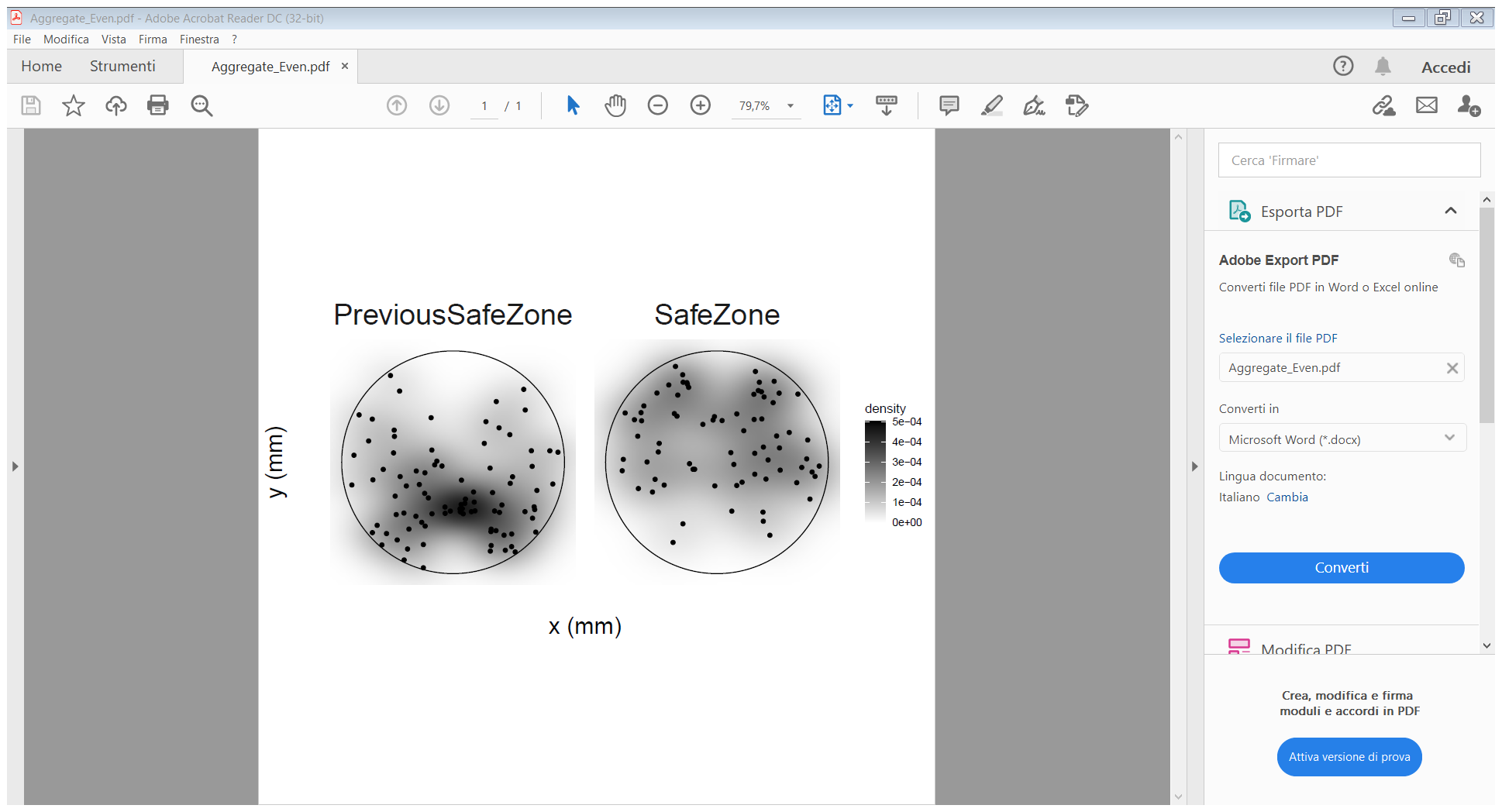

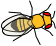

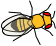

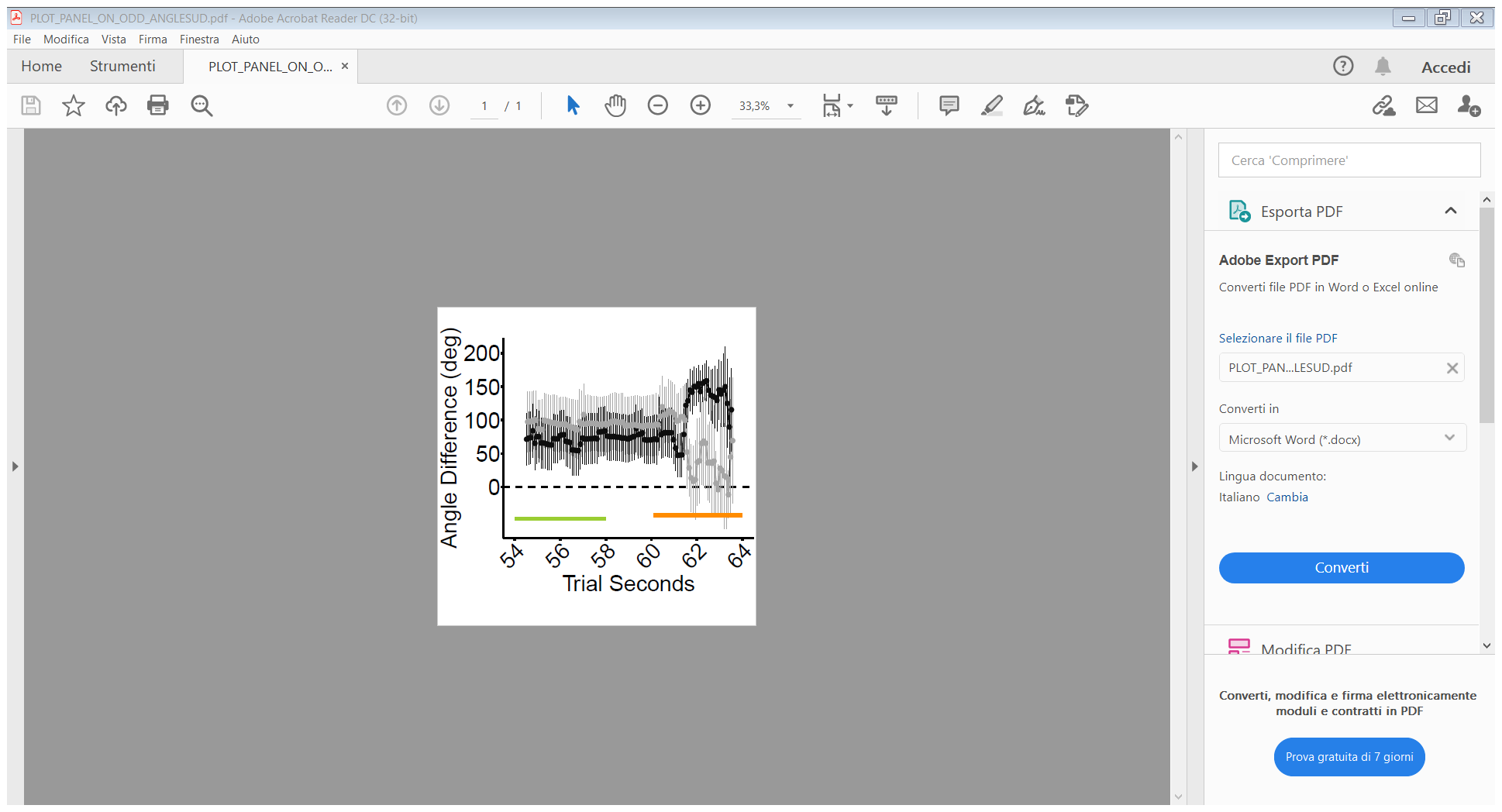

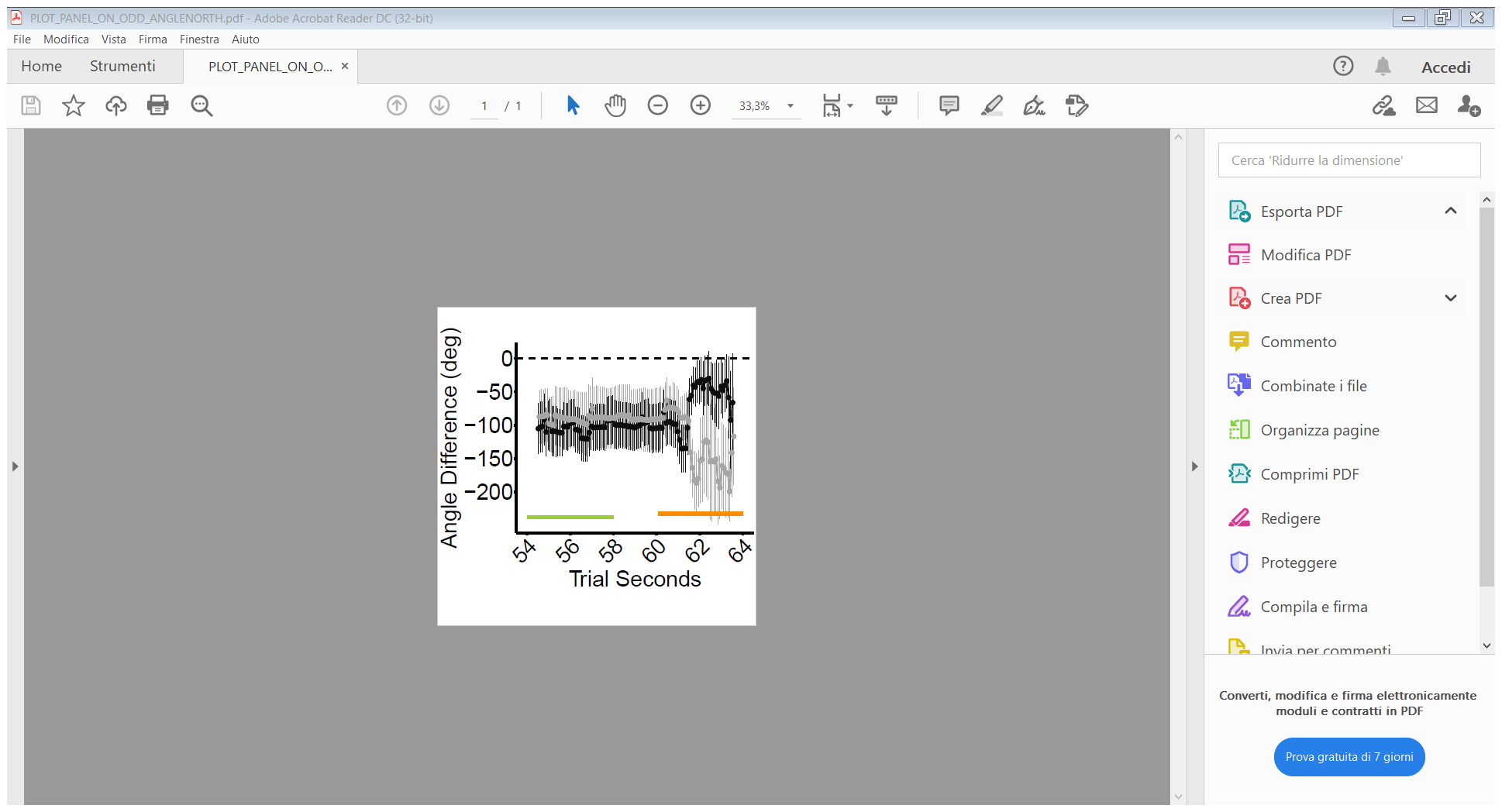

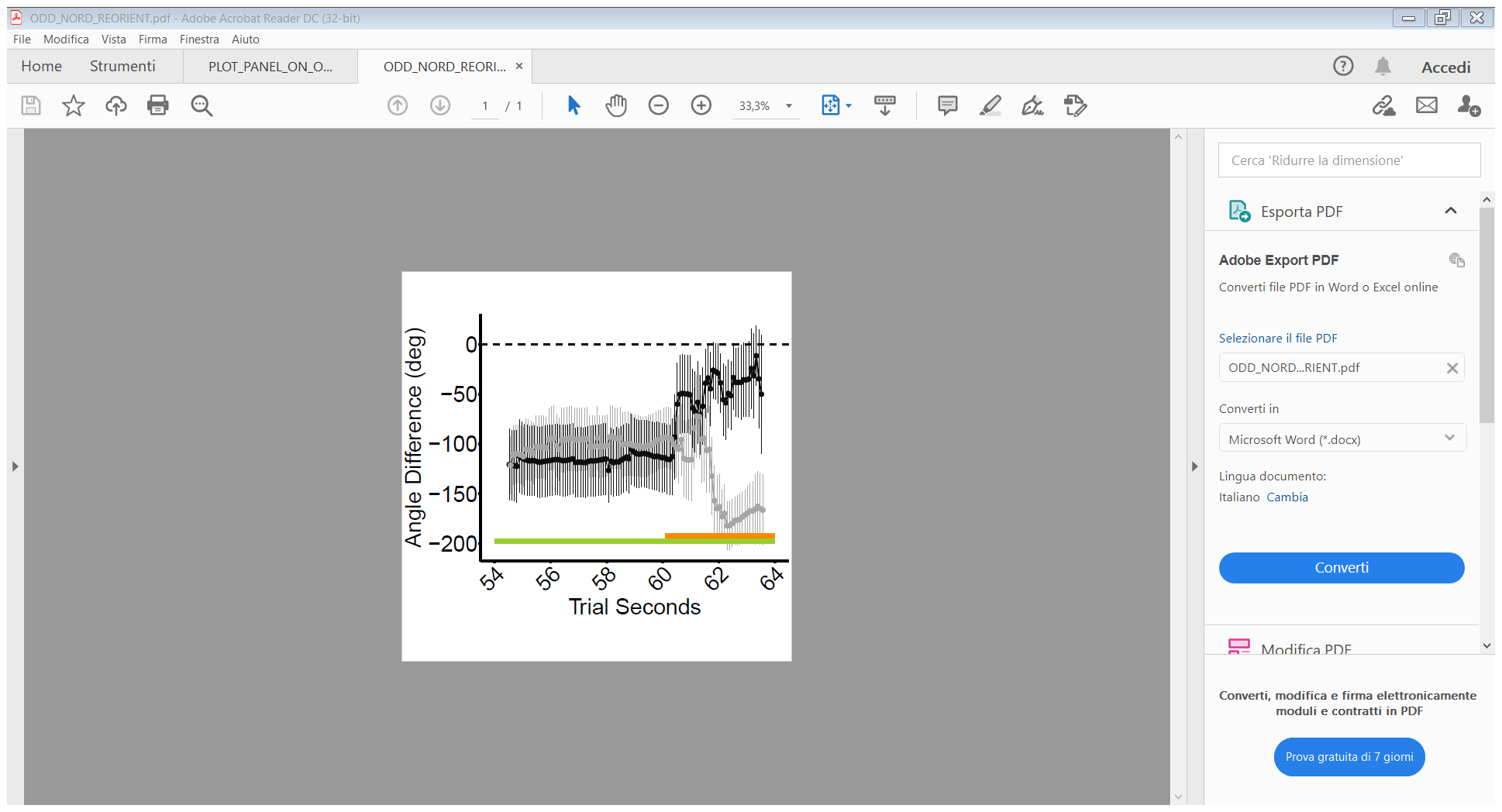

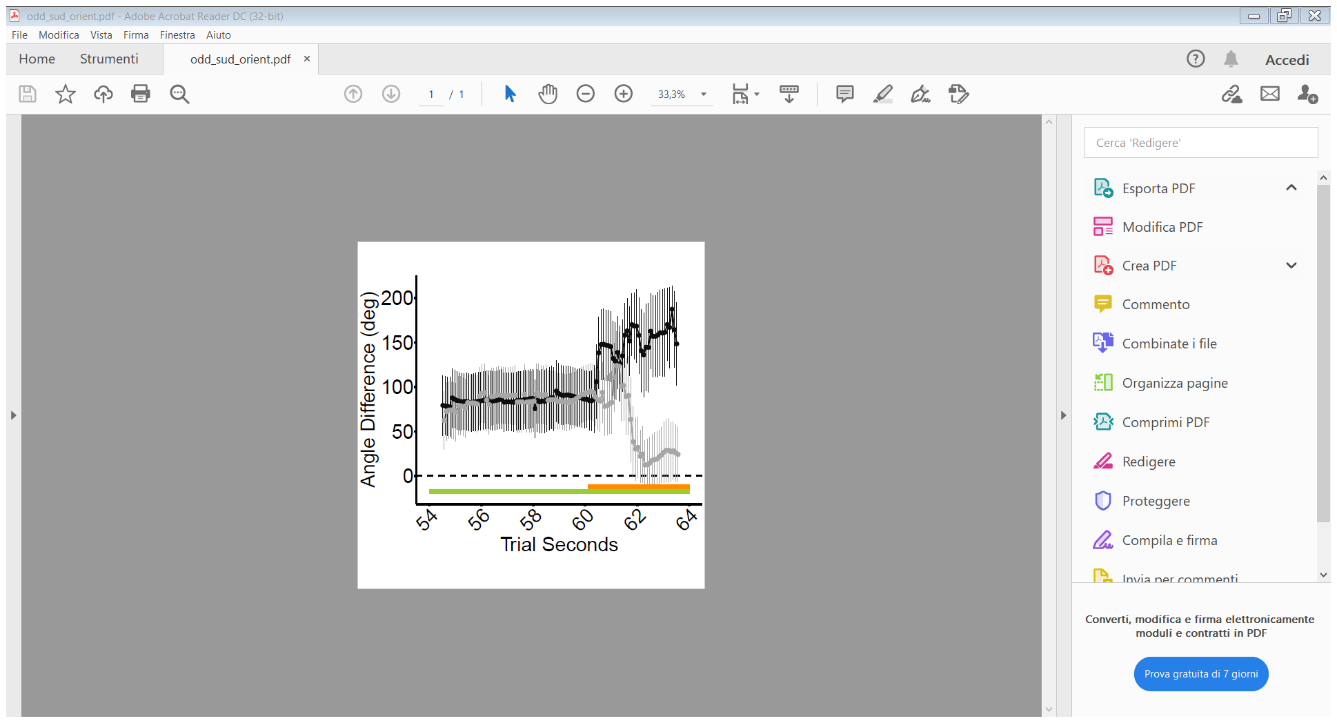

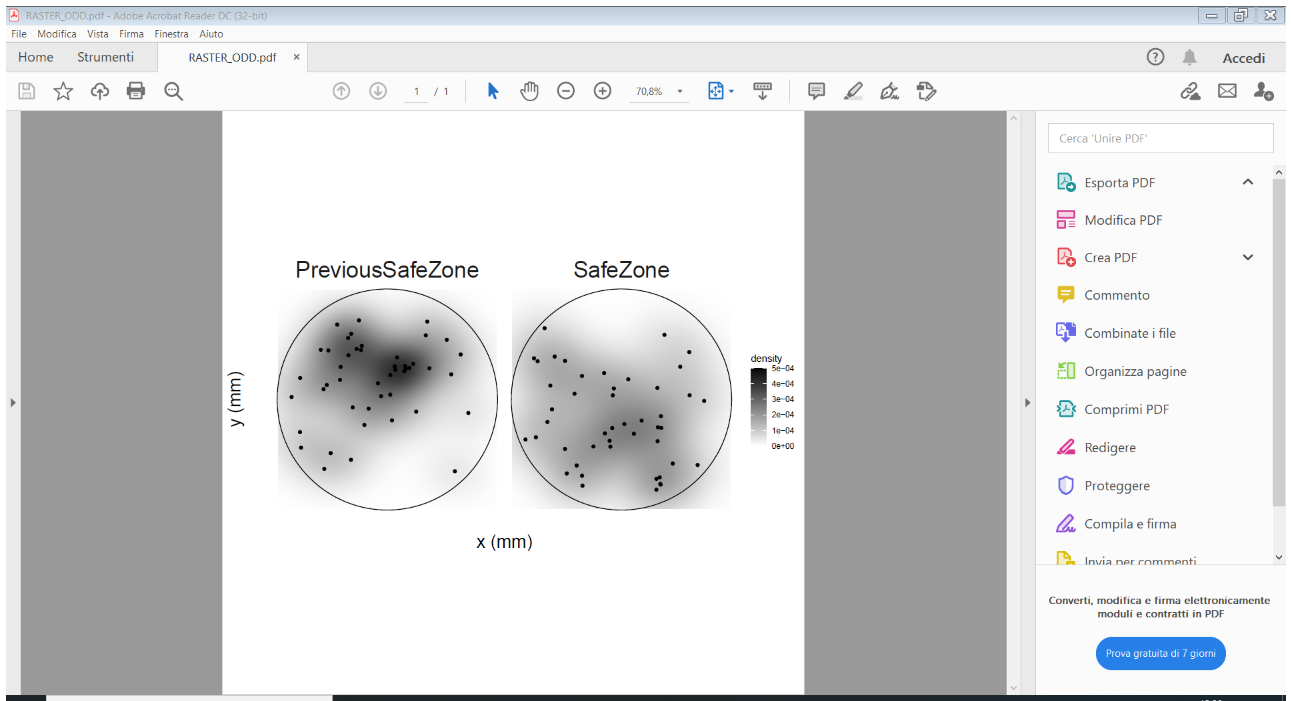

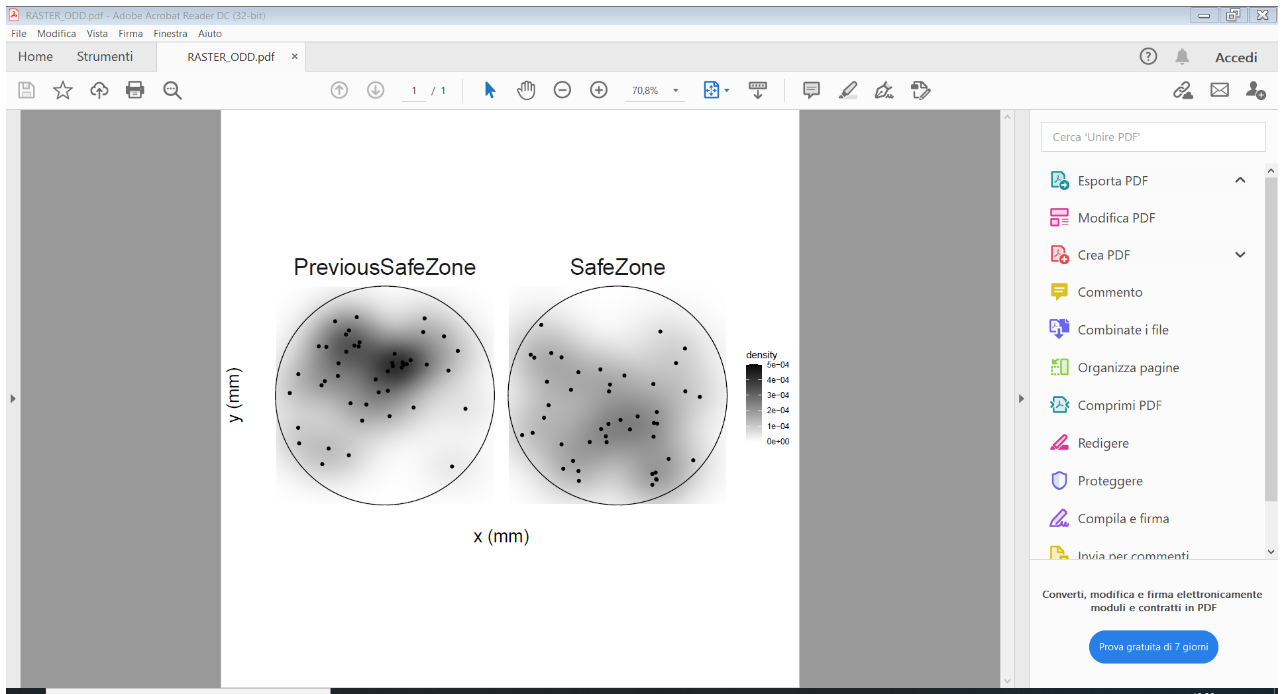

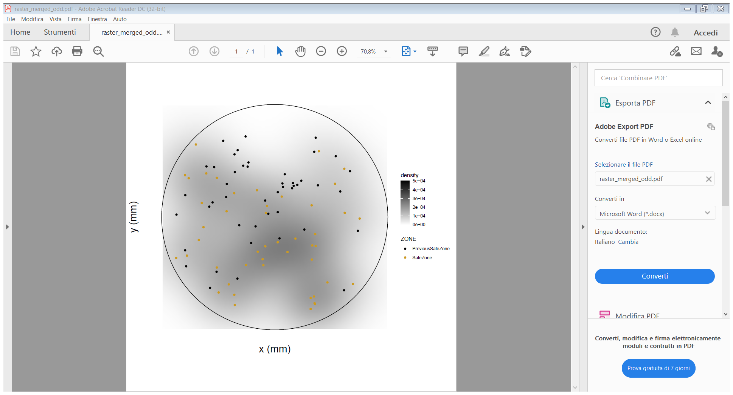

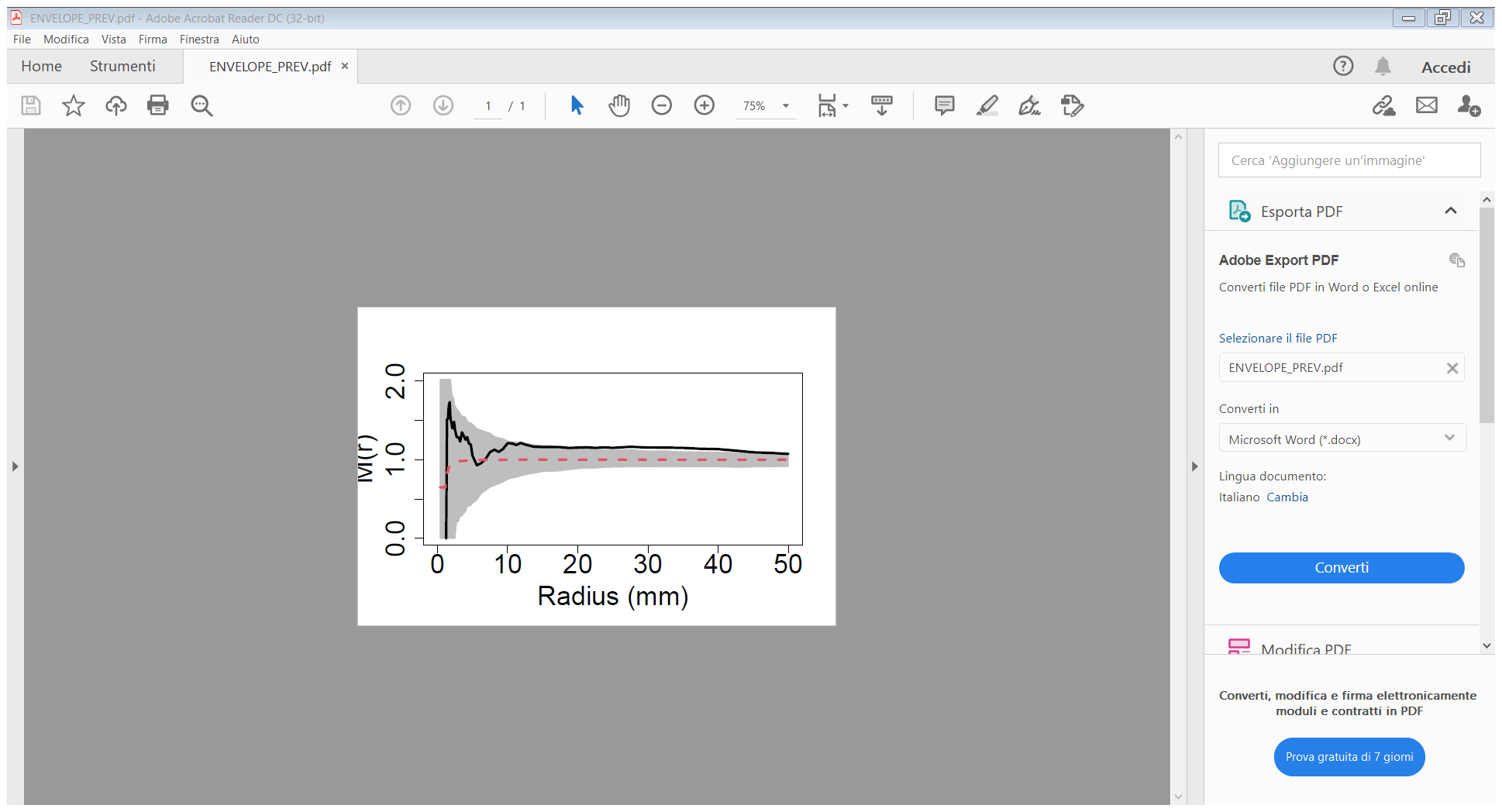

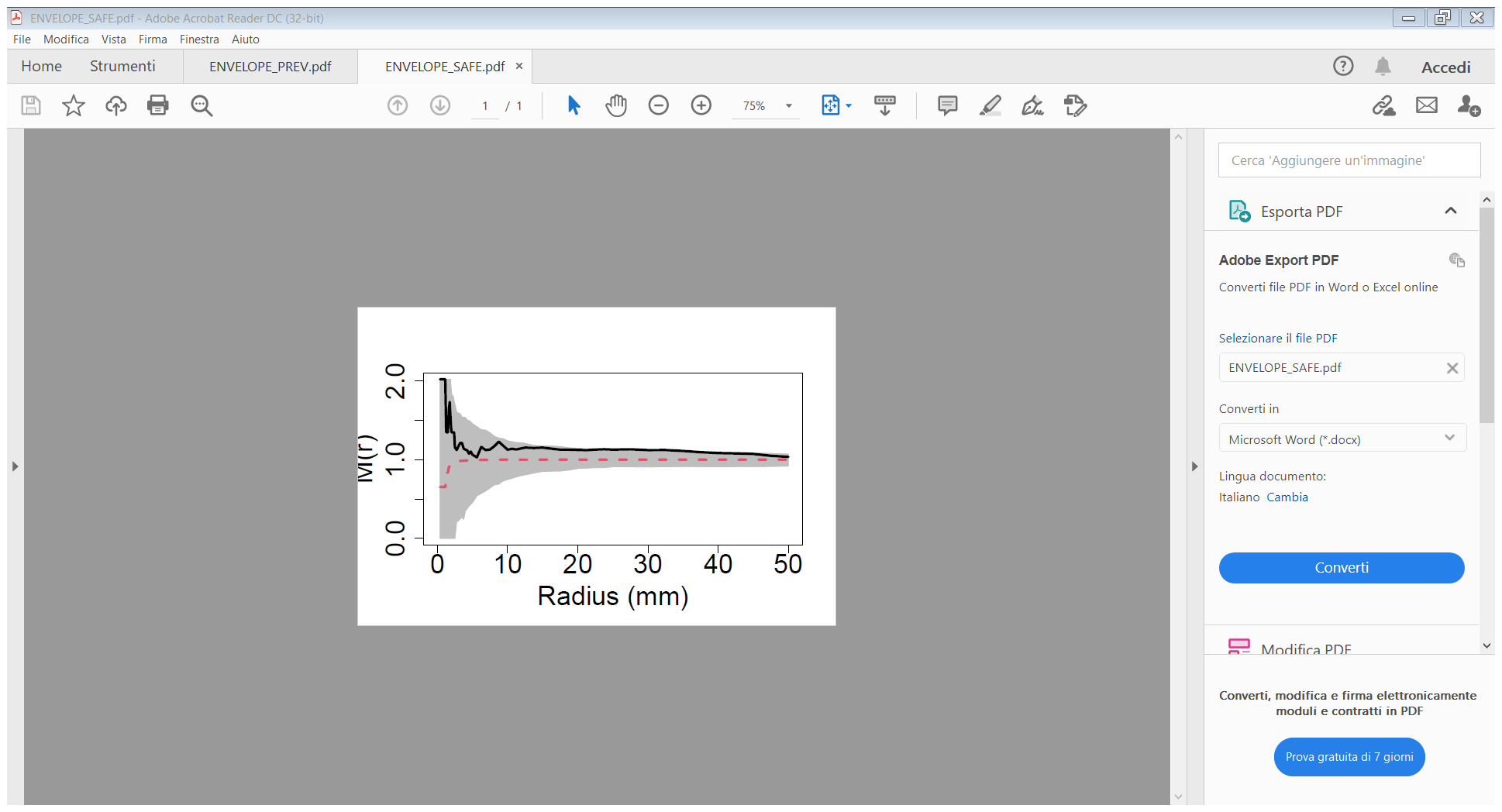

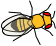

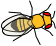

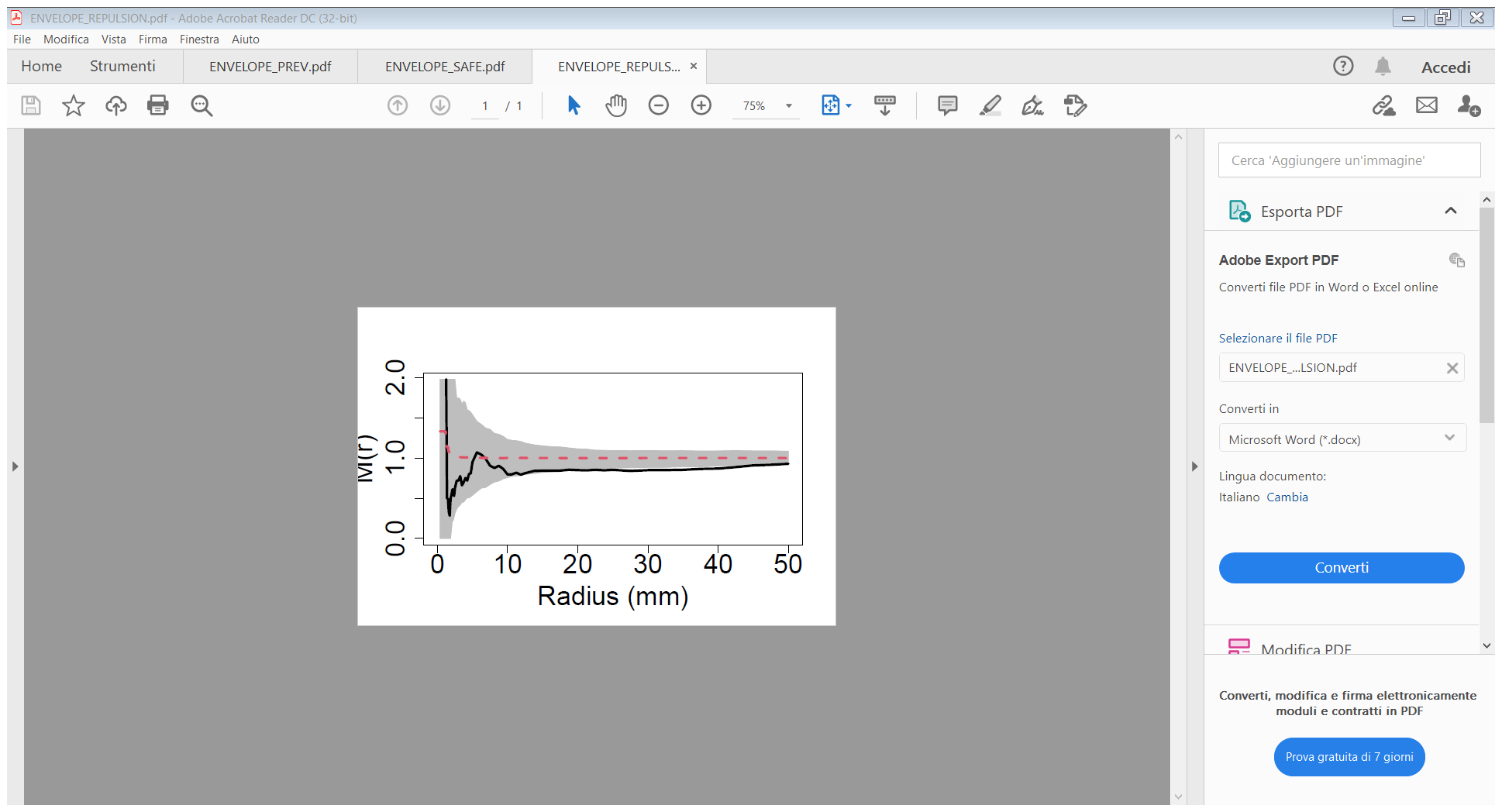


residency

residency

α

α

Bar approached:

Bar approached:

**A**

**B**

**C**

**D**

**E**

**F**

**G**

**H**

M(r)

M(r)

M(r)

α

α

Bar approached:

**I**

**J**

**Supplementary Figure S3. Fruit flies resort to the Nearest Neighbour Rule soon after bitter stimulation onset. Odd-numbered trials.**

Horizontal Green bar (e.g., panel C) = visual patterns displayed. When the green bar is absent (e.g., panel I and J) = visual pattern are not displayed, and flies navigate in a homogeneously lit green environment; horizontal orange bar = stimulation triggered according to fly position. Pointrange = mean ± confidence interval around the mean. **A)** Spatial position of fruit flies, at second 60.1, (when the first pulse of optogenetic stimulation is delivered) that first entered the safe zone, marked by the vertical stripe **B)** Marcon and Puech’s M function value (black line) represents the distance between the observed flies positions compared to 10.000 random distribution simulations (red dashed line and grey shading). A value greater than 1 suggests aggregation. No. positions tested = 63, A goodness-of-fit test (p = 0.061) reveals that flies tend to be more aggregated than expected; **C)** fruit flies that entered the safe zone oriented themselves towards that zone after the onset of the bitter stimulation (See also Table S4; No. orientations = 6830, mean difference = 42.1, std error = 2.33, z.ratio = 18.06, p < 0.0001); **D)** Spatial distribution at second 60.1 of fruit flies that will approach the previous safe zone; **E)** M function value is significantly greater than expected under the null hypothesis, suggesting aggregation of fly positions (No. positions tested = 63, p = 0.011); **F)** the fruit flies that entered the previous safe zone oriented themselves towards it (No. orientations = 6830, mean difference = 52.1, std error = 2.49, z.ratio = 20.96, p < 0.0001); **L)** Spatial position of both groups of flies at second 60.1; **G)** M function value assessing whether the two distributions of flies’ position reported in A), D) or G) consist of two distinct aggregates. **H)** The M function is < 1, thus suggesting spatial repulsion between the two groups of flies, indicating that flies entering the safe zone are spatially segregated from flies that entered the previous safe zone (No. positions = 126, p = 0.014). **I)** the fruit flies re-orient themselves towards the expected location of closest the landmark even when the landmark itself is occluded from vision (in this case the vertical stripe), suggesting that flies retrieve the expected position of the visual target from working memory (see also Table S4; No. orientations = 5286, mean difference = 26.1, std error = 3.72, z.ratio = 7.01, p < 0.0001); **J)** as for I), in this case, the horizontal stripe closest landmark occluded from vision (black bar with a red cross on top) towards which, nonetheless, the flies orient themselves (No. orientations = 5286, mean difference = 27.14, std error = 2.84, z.ratio = 9.63, p < 0.0001).

| **FIGURE 1C. VELOCITY (LME)** | df | BIC |
| --- | --- | --- |
| Number of observed velocities: 8490, Flies n: 40 |  |  |
| Velocity ~ Frame_ins_Zone + (1\|Exp) | 4 | 41710 |
| Velocity ~ Frame_ins_Zone + Zone (1\|Exp) | 5 | 38406 |
| Velocity ~ Frame_ins_Zone*Zone + (1\|Exp) | 6 | **38363** |
|  |  |  |
| **FIGURE 2A. NUMBER OF FLIES (GLMM)** | df | BIC |
| Number of observed flies: 89, nTrial: 15 |  |  |
| No.Flies ~ 1 + (1\|nTrial) | 2 | 438.42 |
| No.Flies ~ Zone + (1\|nTrial) | 3 | 437.69 |
| No.Flies ~ nperiod + (1\|nTrial) | 4 | **395.69** |
| No.Flies ~ Zone + nperiod + (1\|nTrial) | 5 | **395.32** |
| No.Flies ~ Zone*nperiod + (1\|nTrial) | 7 | 403.72 |
|  |  |  |
| **FIGURE 2B. NUMBER OF VISITS (GLMM)** | df | BIC |
| Number of observed visits: 151, nTrial: 15 |  |  |
| No.Visits ~ 1 + (1\|nTrial) | 2 | 597.23 |
| No.Visits ~ Zone + (1\|nTrial) | 3 | 600.48 |
| No.Visits ~ nperiod + (1\|nTrial) | 4 | **553.17** |
| No.Visits ~ Zone + nperiod + (1\|nTrial) | 5 | 557.03 |
| No.Visits ~ Zone*nperiod + (1\|nTrial) | 7 | 566.87 |
|  |  |  |
| **FIGURE 2E. Angle Difference Towards Northern target (LME)** | df | BIC |
| Number of observed angle differences: 8937, Flies n: 39 |  |  |
| Angle Diff ~ 1 + (1\|Exp/nTrial) | 4 | 102282 |
| Angle Diff ~ nperiod + (1\|Exp/nTrial) | 5 | 102264 |
| Angle Diff ~ nperiod + target + (1\|Exp/nTrial) | 6 | 102269 |
| Angle Diff ~ nperiod*target + (1\|Exp/nTrial) | 7 | **101898** |
|  |  |  |
| **FIGURE 2H. Angle Difference Towards Southern target (LME)** | df | BIC |
| Number of observed angle differences: 8937, Flies n: 39 |  |  |
| Angle Diff ~ 1 + (1\|Exp/nTrial) | 4 | 102058 |
| Angle Diff ~ nperiod + (1\|Exp/nTrial) | 5 | 102054 |
| Angle Diff ~ nperiod + target + (1\|Exp/nTrial) | 6 | 102062 |
| Angle Diff ~ nperiod*target + (1\|Exp/nTrial) | 7 | **101648** |
|  |  |  |
| **Zone entered (binomial: Yes/No) as a function of position and orientation at second 60.1 – during EVEN-numbered trials** | df | BIC |
| Number of observed positions/orientations: 95, Flies n: 39 |  |  |
| Zone South ~ 1 + (1\|Exp/nTrial) | 3 | 144.84 |
| Zone South ~ Distance + (1\|Exp/nTrial) | 4 | **144.66** |
| Zone South ~ Distance + Angle Diff + (1\|Exp/nTrial) | 5 | 149.07 |
| Zone South ~ Distance*Angle Diff + (1\|Exp/nTrial) | 6 | 153.12 |
|  |  |  |
| Zone North ~1 + (1\|Exp/nTrial) | 3 | 144.84 |
| Zone North ~ Distance + (1\|Exp/nTrial) | 4 | **140.22** |
| Zone North ~ Distance + Angle Diff + (1\|Exp/nTrial) | 5 | 144.18 |
| Zone North ~ Distance*Angle Diff + (1\|Exp/nTrial) | 6 | 147.61 |
|  |  |  |
| **FIGURE 2K. Angle Difference Towards Northern target (LME)** | df | BIC |
| Number of observed angle differences: 6917, Flies n: 26 |  |  |
| Angle Diff ~ 1 + (1\|Exp/nTrial) | 4 | 79605 |
| Angle Diff ~ nperiod + (1\|Exp/nTrial) | 5 | 79602 |
| Angle Diff ~ nperiod + target + (1\|Exp/nTrial) | 6 | 79610 |
| Angle Diff ~ nperiod*target + (1\|Exp/nTrial) | 7 | **79194** |
|  |  |  |
| **FIGURE 2L. Angle Difference Towards Southern target (LME)** | df | BIC |
| Number of observed angle differences: 6917, Flies n: 26 |  |  |
| Angle Diff ~ 1 + (1\|Exp/nTrial) | 4 | 79701 |
| Angle Diff ~ nperiod + (1\|Exp/nTrial) | 5 | 79702 |
| Angle Diff ~ nperiod + target + (1\|Exp/nTrial) | 6 | 79710 |
| Angle Diff ~ nperiod*target + (1\|Exp/nTrial) | 7 | **79274** |
|  |  |  |
| **SUPPL. FIGURE S3C. Angle Difference Towards Southern target (LME)** | df | BIC |
| Number of observed angle differences: 6830, Flies n: 37 |  |  |
| Angle Diff ~ 1 + (1\|Exp/nTrial) | 4 | 77307 |
| Angle Diff ~ nperiod + (1\|Exp/nTrial) | 5 | 77315 |
| Angle Diff ~ nperiod + target + (1\|Exp/nTrial) | 6 | 77321 |
| Angle Diff ~ nperiod*target + (1\|Exp/nTrial) | 7 | **76659** |
|  |  |  |
| **SUPPL. FIGURE S3F. Angle Difference Towards Northern target (LME)** | df | BIC |
| Number of observed angle differences: 6830, Flies n: 37 |  |  |
| Angle Diff ~ 1 + (1\|Exp/nTrial) | 4 | 77469 |
| Angle Diff ~ nperiod + (1\|Exp/nTrial) | 5 | 77477 |
| Angle Diff ~ nperiod + target + (1\|Exp/nTrial) | 6 | 77485 |
| Angle Diff ~ nperiod*target + (1\|Exp/nTrial) | 7 | **76749** |
|  |  |  |
| **SUPPL. FIGURE S3I. Angle Difference Towards Southern target (LME)** | df | BIC |
| Number of observed angle differences: 5286, Flies n: 27 |  |  |
| Angle Diff ~ 1 + (1\|Exp/nTrial) | 4 | 60990 |
| Angle Diff ~ nperiod + (1\|Exp/nTrial) | 5 | 60975 |
| Angle Diff ~ nperiod + target + (1\|Exp/nTrial) | 6 | 60984 |
| Angle Diff ~ nperiod*target + (1\|Exp/nTrial) | 7 | **60838** |
|  |  |  |
| **SUPPL. FIGURE S3J. Angle Difference Towards Northern target (LME)** | df | BIC |
| Number of observed angle differences: 5286, Flies n: 27 |  |  |
| Angle Diff ~ 1 + (1\|Exp/nTrial) | 4 | 61010 |
| Angle Diff ~ nperiod + (1\|Exp/nTrial) | 5 | 61011 |
| Angle Diff ~ nperiod + target + (1\|Exp/nTrial) | 6 | 61019 |
| Angle Diff ~ nperiod*target + (1\|Exp/nTrial) | 7 | **60884** |
|  |  |  |
| **Zone entered (binomial: Yes/No) as a function of position and orientation at second 60.1 – during ODD-numbered trials** | df | BIC |
| Number of observed positions/orientations: 75, Flies n: 37 |  |  |
| Zone South ~ 1 + (1\|Exp/nTrial) | 3 | 116.27 |
| Zone South ~ Distance + (1\|Exp/nTrial) | 4 | **107.57** |
| Zone South ~ Distance + Angle Diff + (1\|Exp/nTrial) | 5 | 111.86 |
| Zone South ~ Distance*Angle Diff + (1\|Exp/nTrial) | 6 | 115.03 |
|  |  |  |
| Zone North ~1 + (1\|Exp/nTrial) | 3 | 116.27 |
| Zone North ~ Distance + (1\|Exp/nTrial) | 4 | **103.86** |
| Zone North ~ Distance + Angle Diff + (1\|Exp/nTrial) | 5 | 107.87 |
| Zone North ~ Distance*Angle Diff + (1\|Exp/nTrial) | 6 | 111.97 |
|  |  |  |

**Table S4. Models details related to Figure 1, Figure 2, and Supplementary Figure S3.**

LME = Linear Mixed-Effects (model); GLMM = Generalised Linear Mixed Model (in these models, family = poisson); BIC = Bayesian Information Criterion; (1|x) = variable “x” is considered a random effect; (1|x/y) = nested random effect of variable *y* for each *x*; Exp = experiment (single fly); Frame_ins_Zone = frames since the entrance of the fly into a specific zone; nTrial = trial number; nperiod = period number (each period consists of 10 seconds). The “*” sign = interaction between the variables. The variable before the tilde (~) is the outcome to be estimated by the regression model(s). The explanatory variables are placed after the tilde. The models are displayed in increasing order of degrees of freedom (df). BIC was taken into consideration for the selection of the best model. The best model for each figure is the one with the lowest BIC (in bold). If, for example, the best model for Figure 2A (No.Flies ~ nperiod + (1|nTrial)) has a lower BIC than the model considering also zone (No.Flies ~ Zone + nperiod + (1|nTrial)), it means that the role of zone in determining the number of flies is not significant (i.e., the number of flies in the different zones is not significantly different).

Regarding the binomial models employed to predict which zone will be entered based on fly position and orientations at second 60.1, we also tested an additional set of models, but this time considering the mean orientations in the four seconds before the stimulation onset (and not only the orientation at second 60.1, in order to account for fly heading direction). Nevertheless, the fly mean orientation does not improve a model that already accounted for the fly position in the arena. A similar conclusion is also reached for Zone South and for the odd-numbered trials.

Before opting for including training progression (“nTrial”) as random intercept in the models related to Figure 2A and 2B, we ascertained that the effect of this variable (transformed to continuous) on the studied outcomes was negligible (i.e., that there was no trend towards an increase or reduction in the number of visits/flies throughout training in the seconds before the optogenetic stimulation or the ten seconds immediately after).

| Model | df | BIC |
| --- | --- | --- |
| No. Visits ~ Zone + (1\|Exp) | 3 | **1950.9** |
| No. Visits ~ Zone + nTrial + (1\|Exp) | 4 | 1957.0 |
| No. Visits ~ Zone*nTrial + (1\|Exp) | 5 | 1963.7 |
|  |  |  |
| No. Flies ~ Zone + (1\|Exp) | 3 | **425.66** |
| No. Flies ~ Zone + nTrial + (1\|Exp) | 4 | 430.14 |
| No. Flies ~ Zone*nTrial + (1\|Exp) | 5 | 434.57 |

**
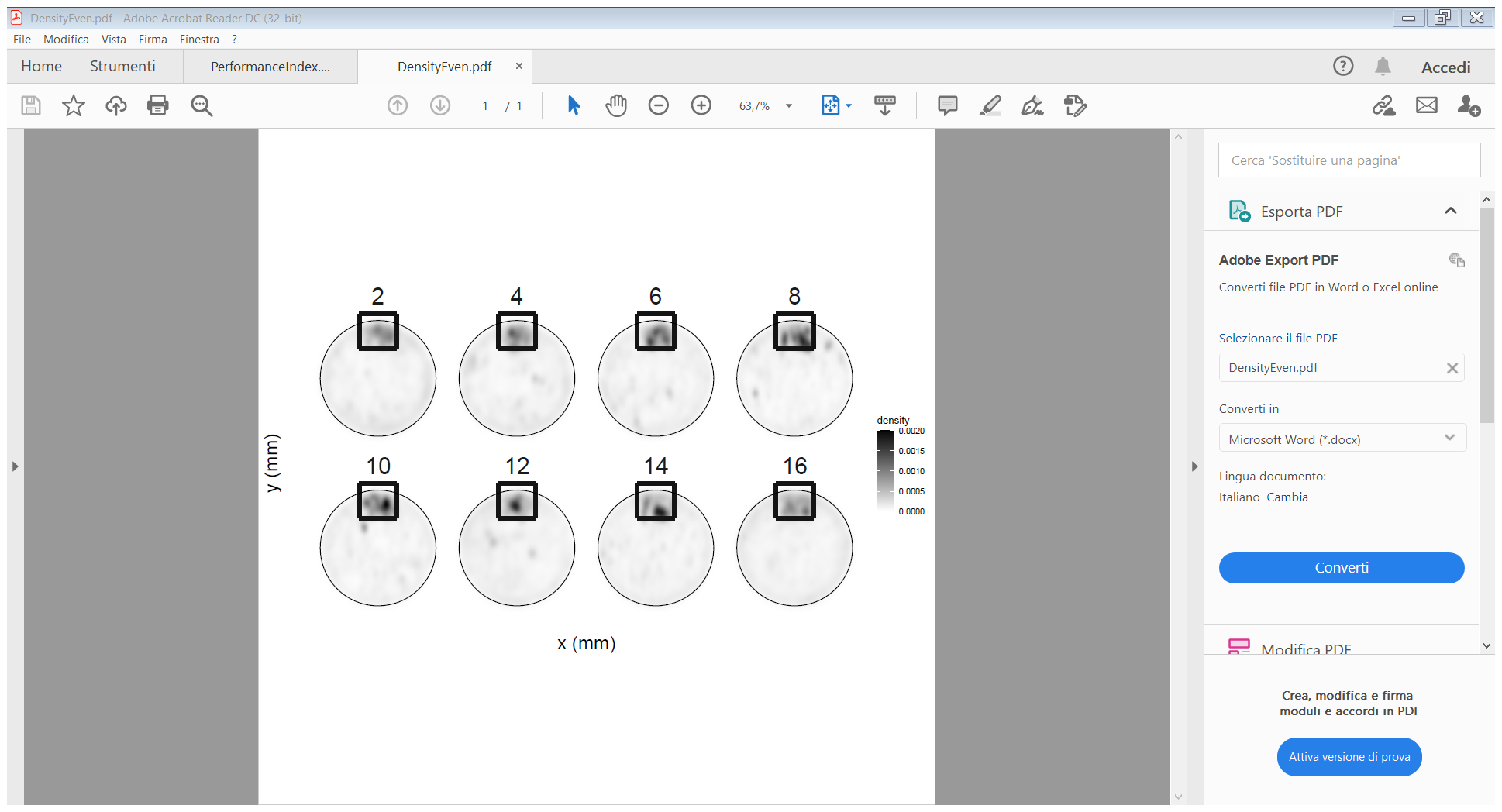

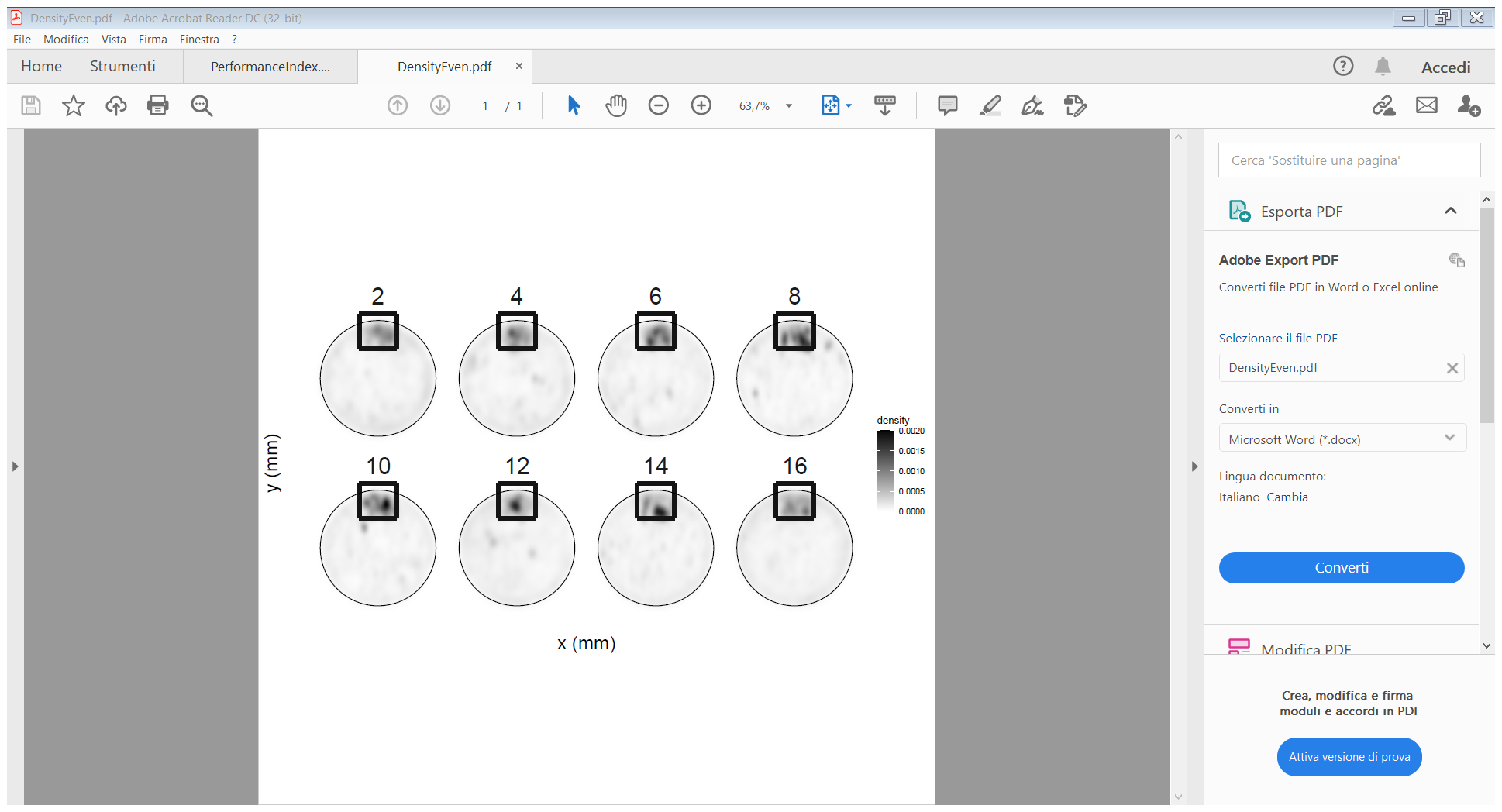

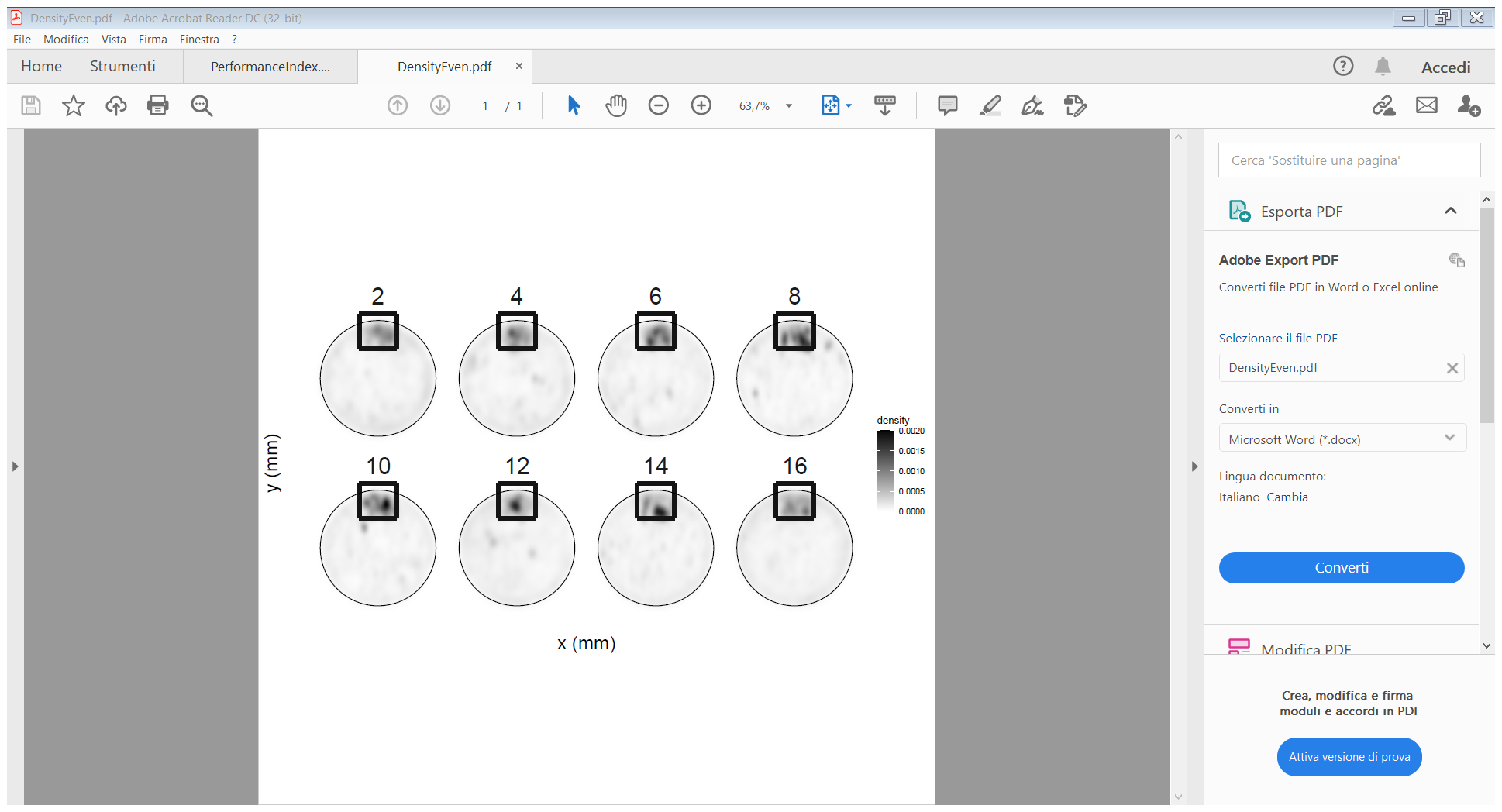

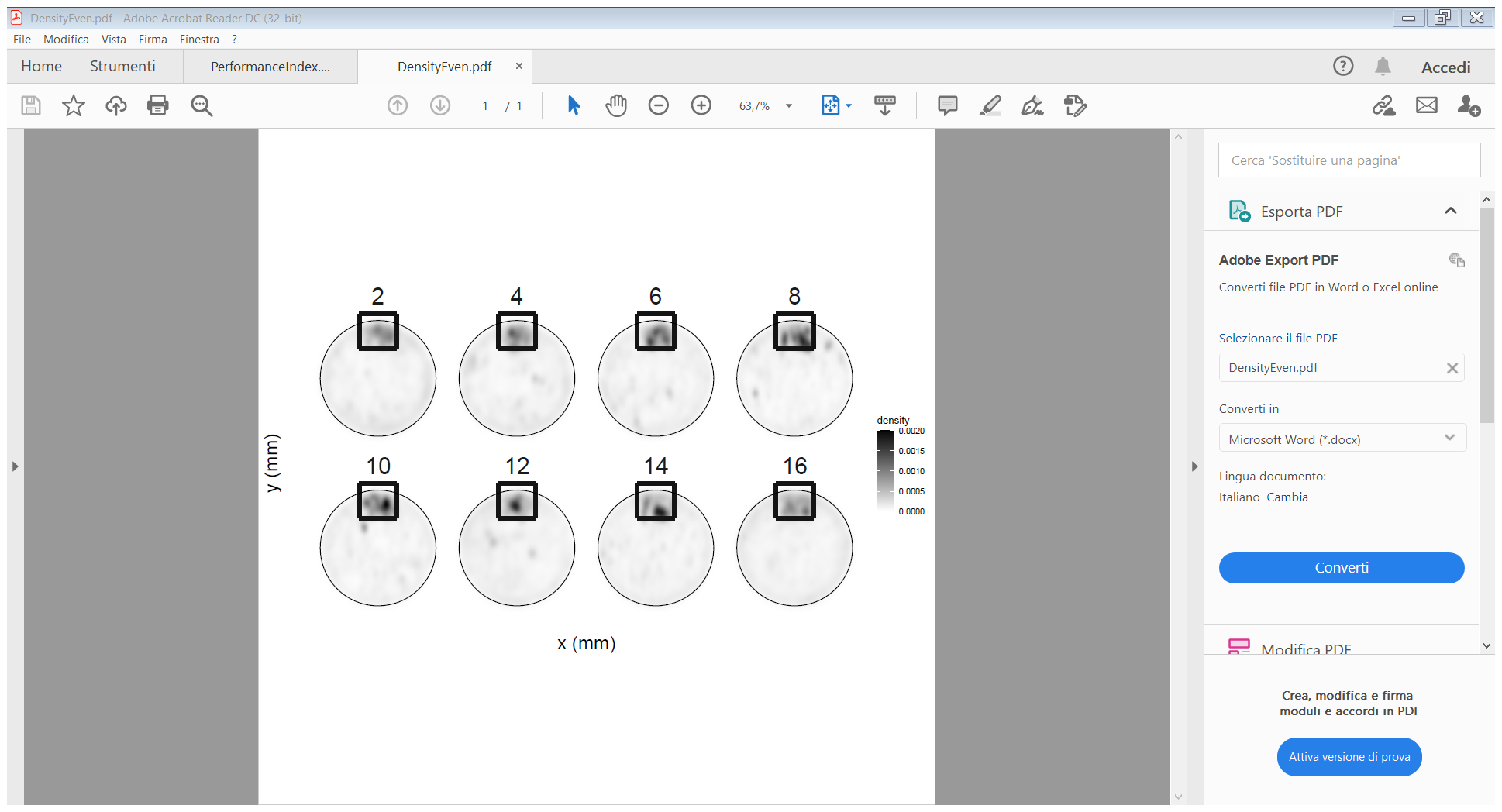

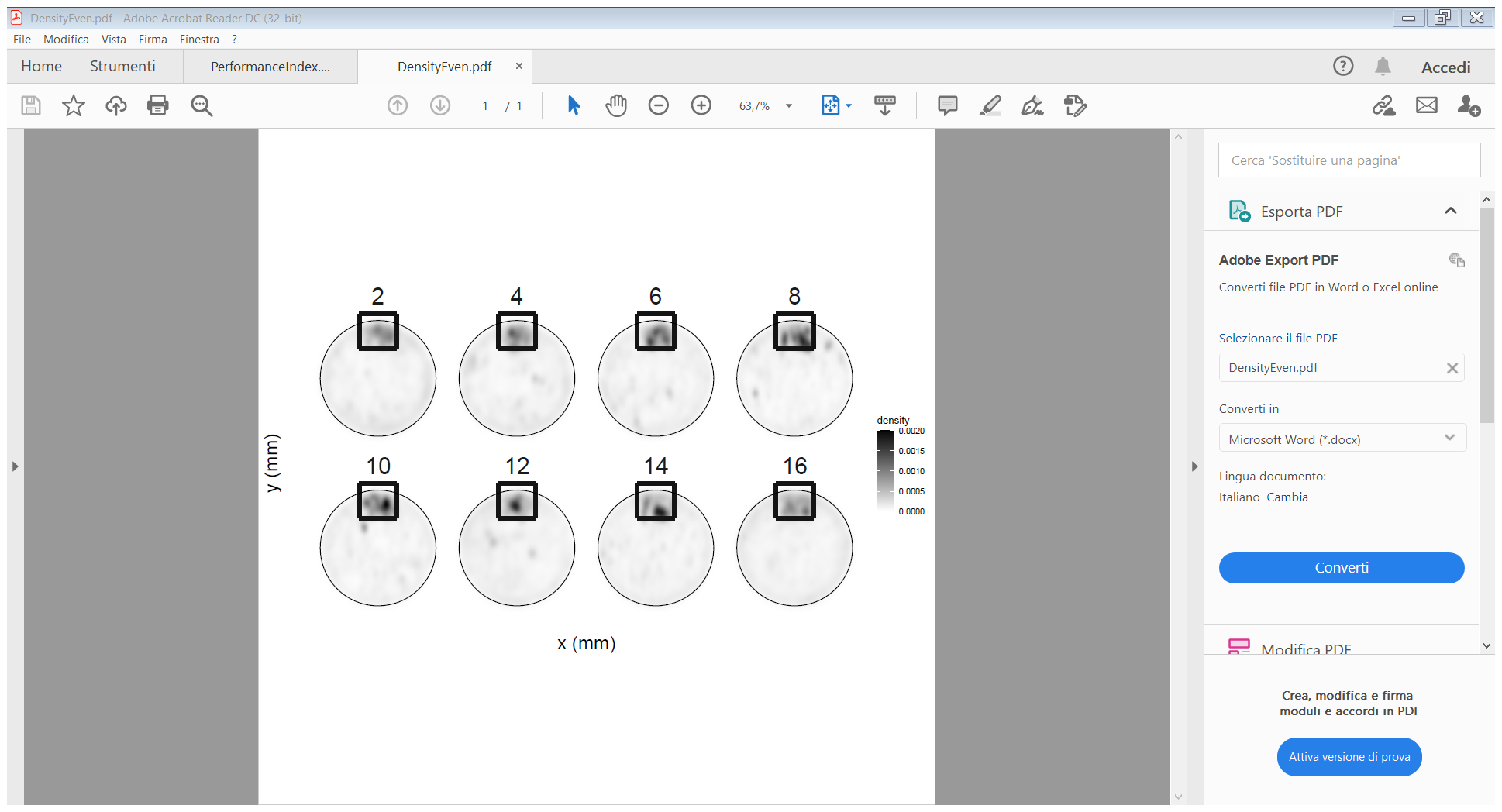

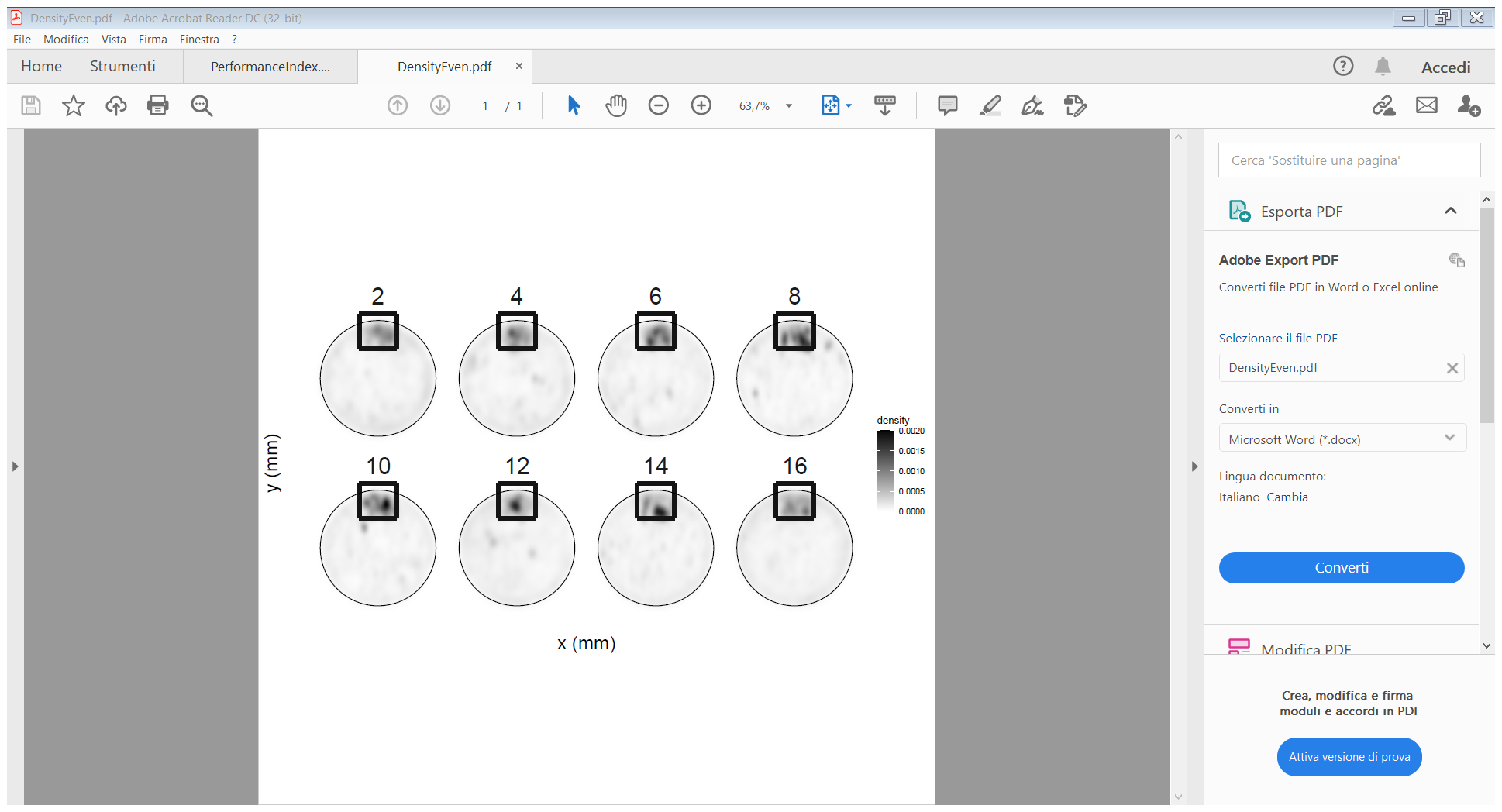

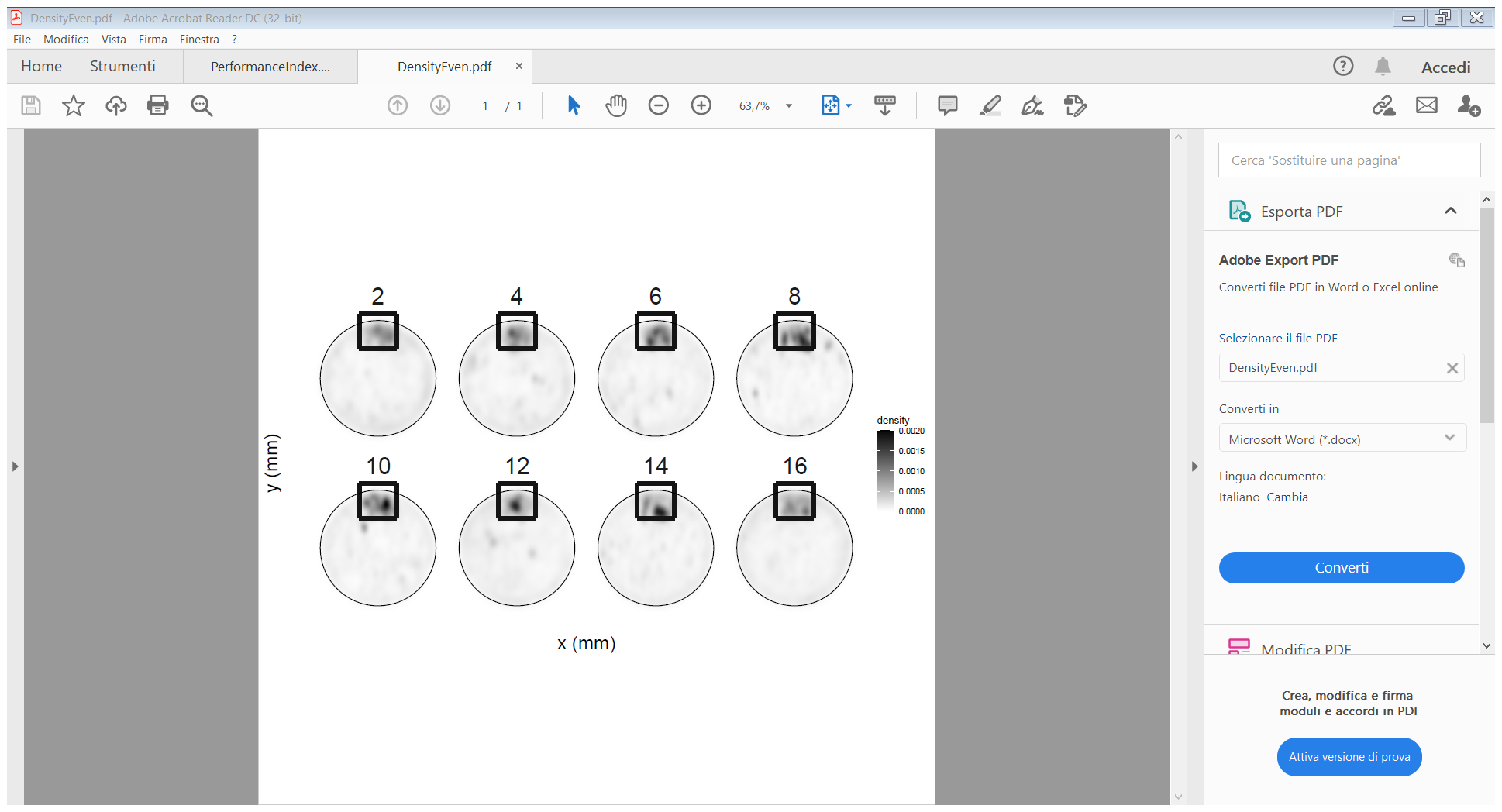

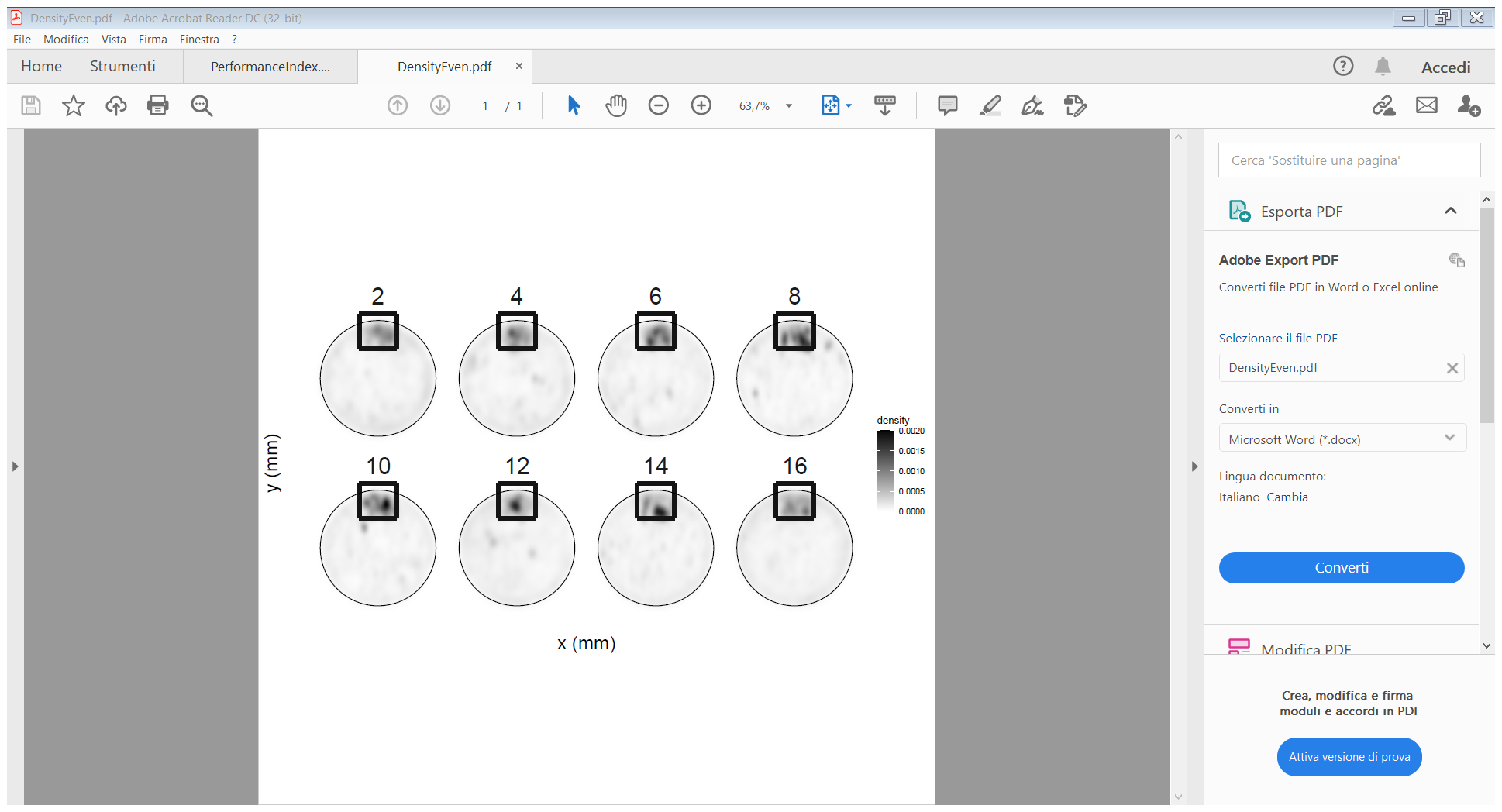

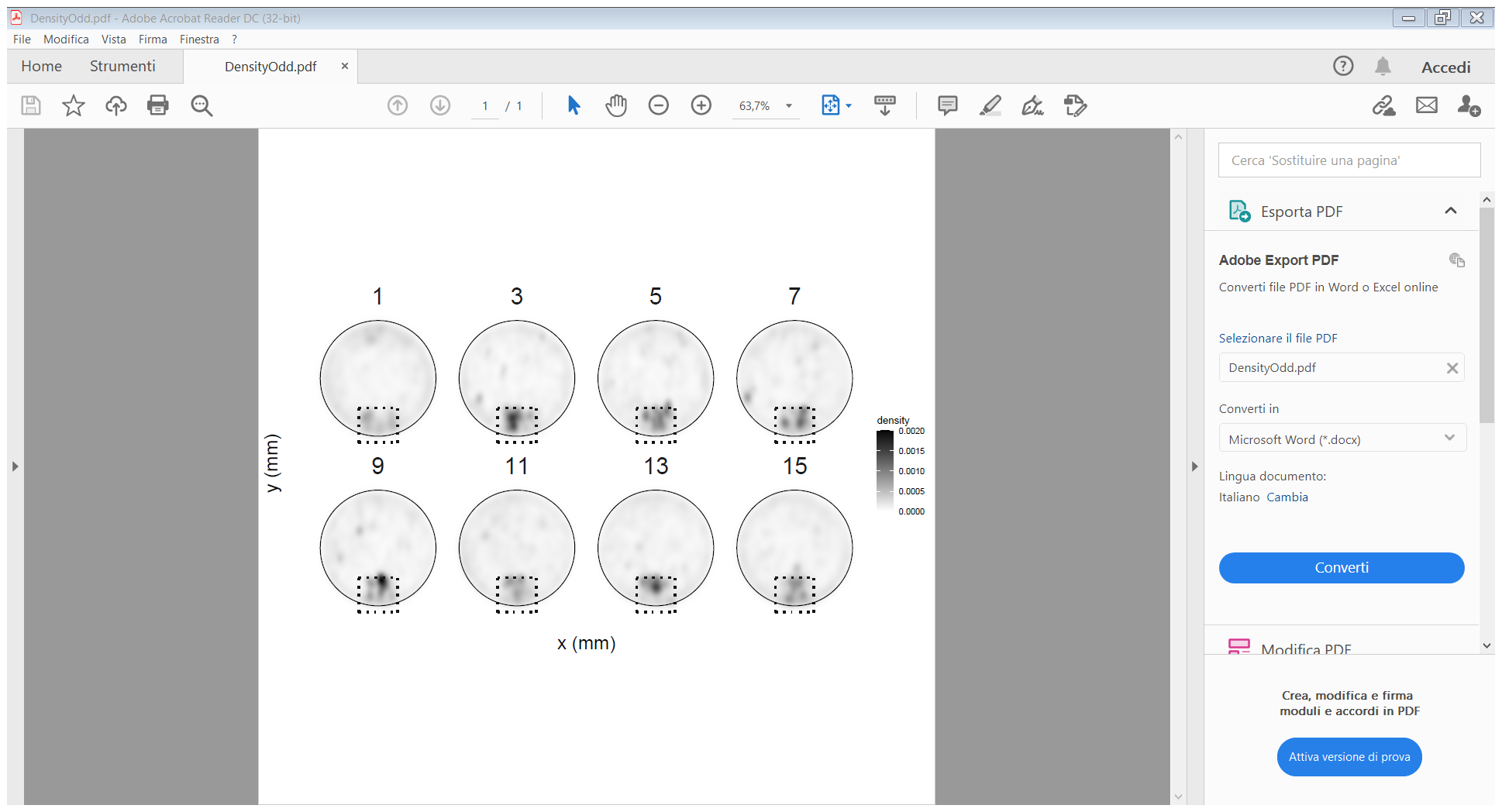

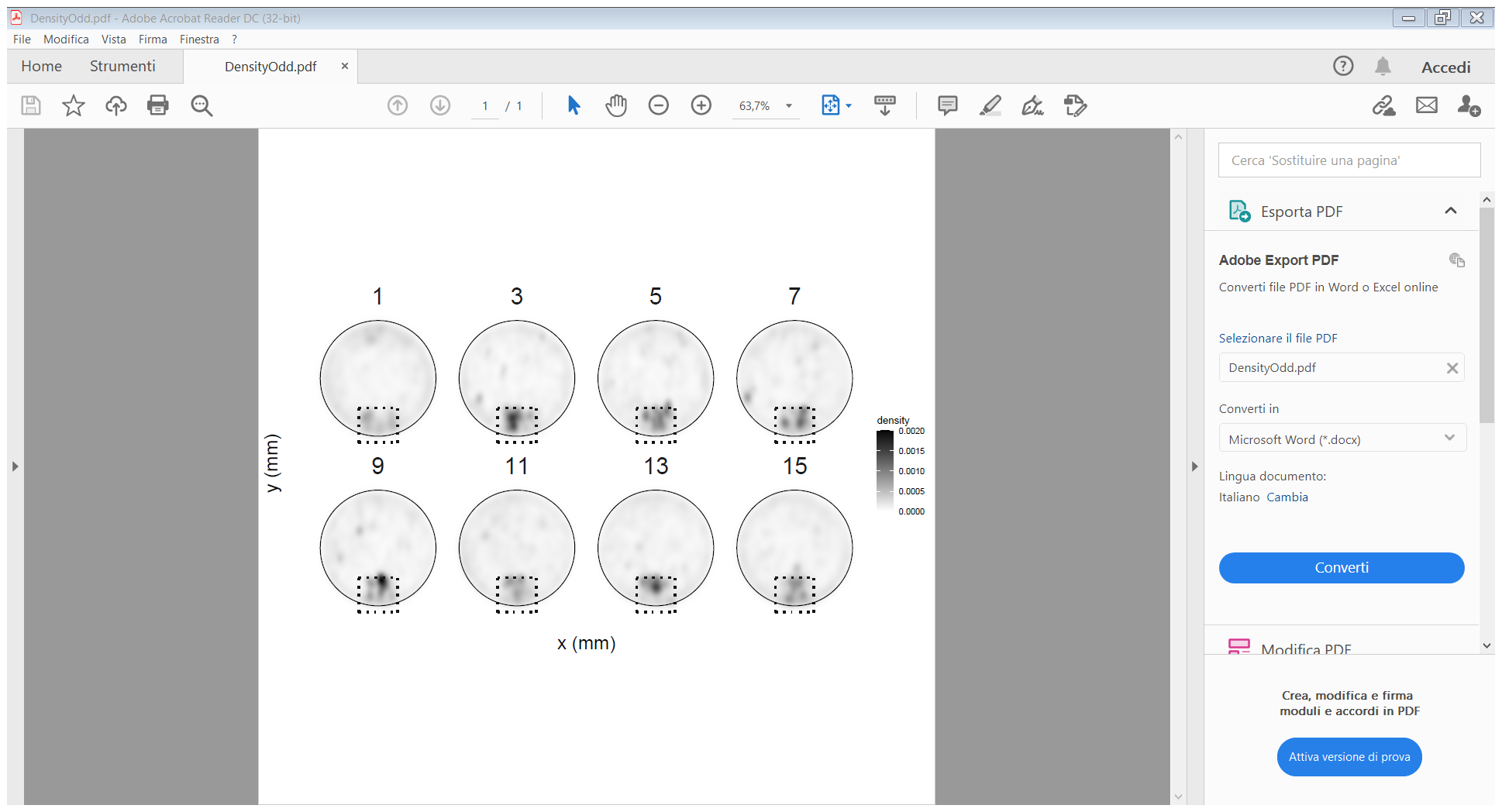

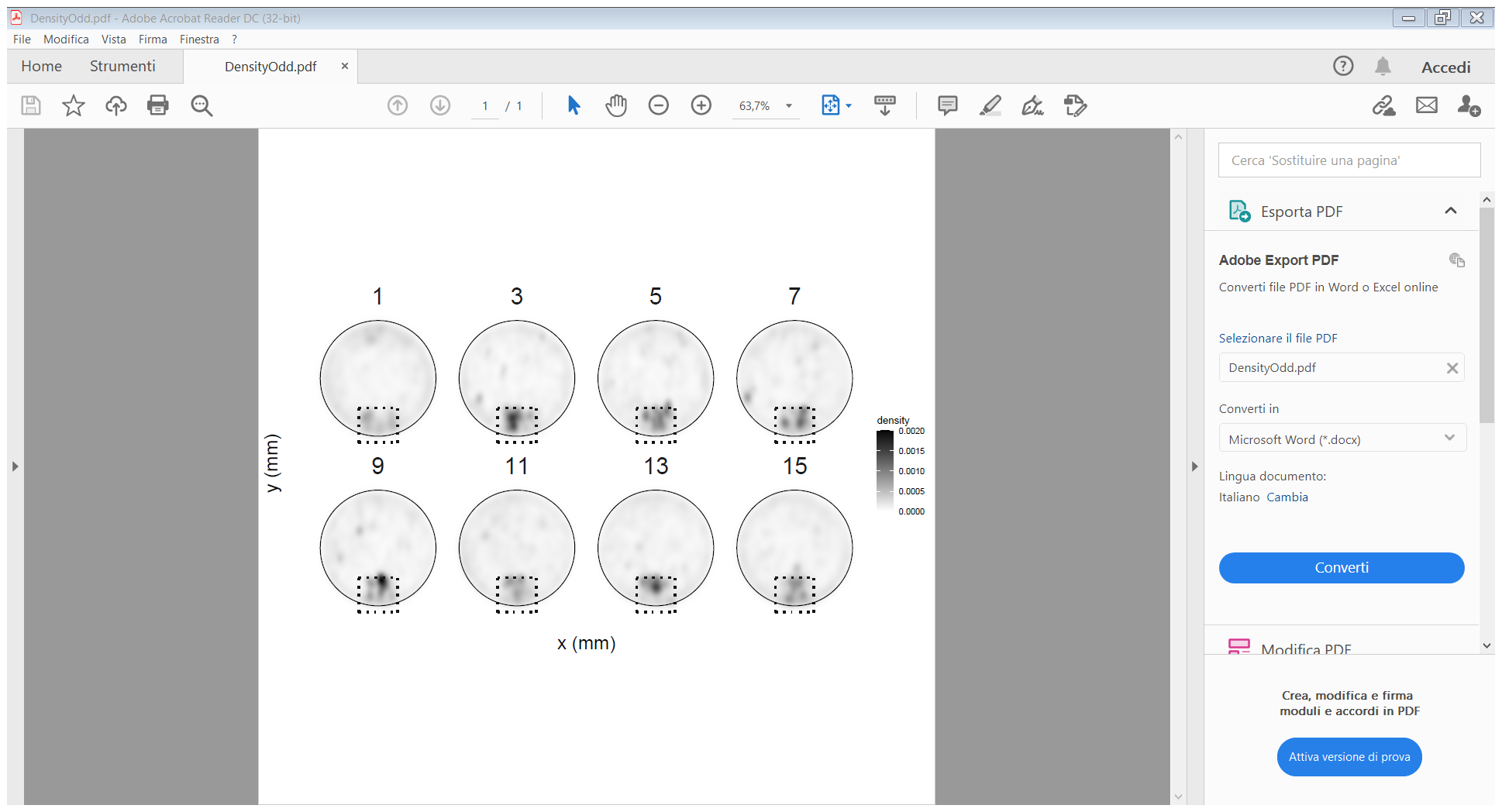

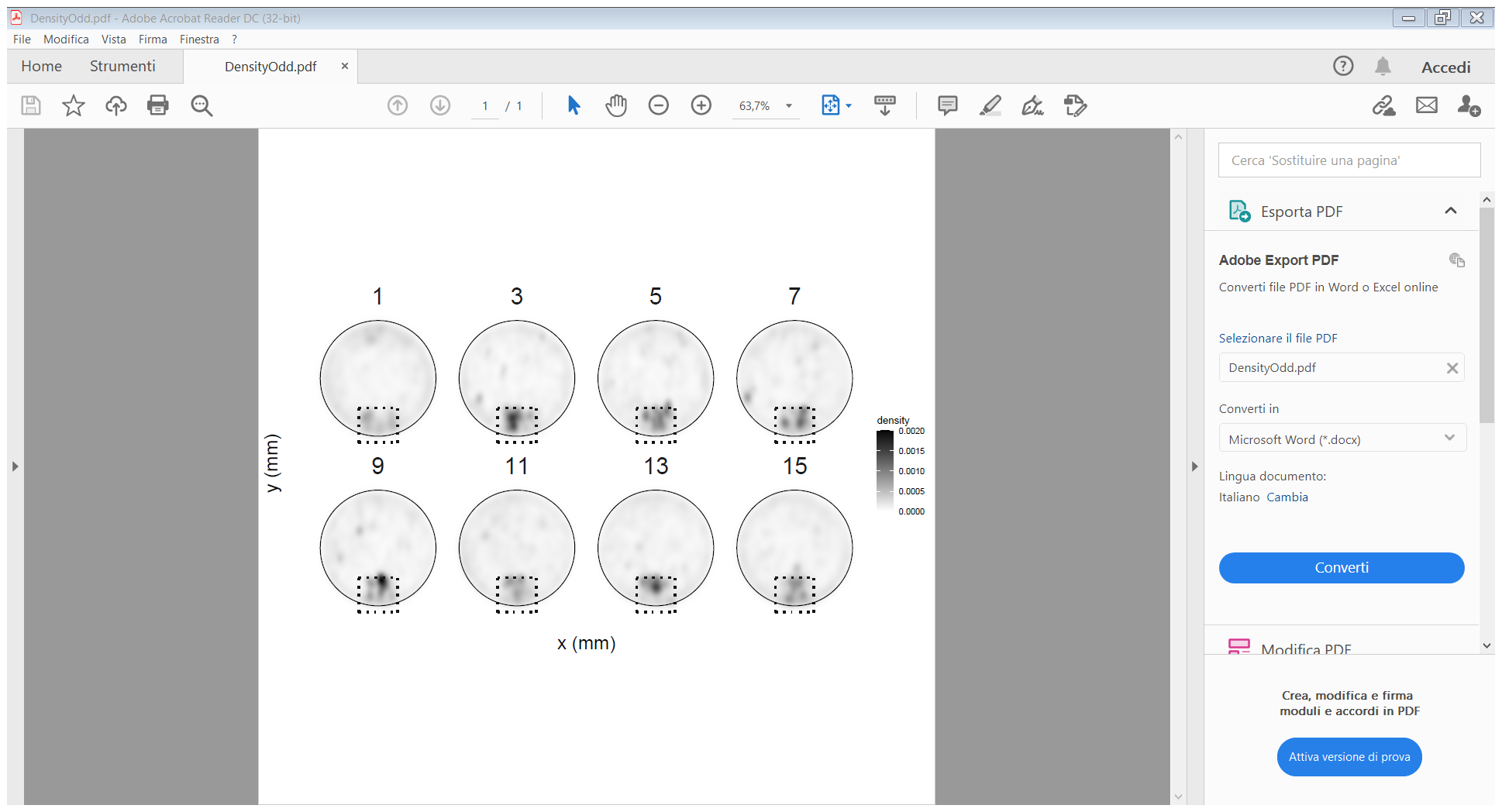

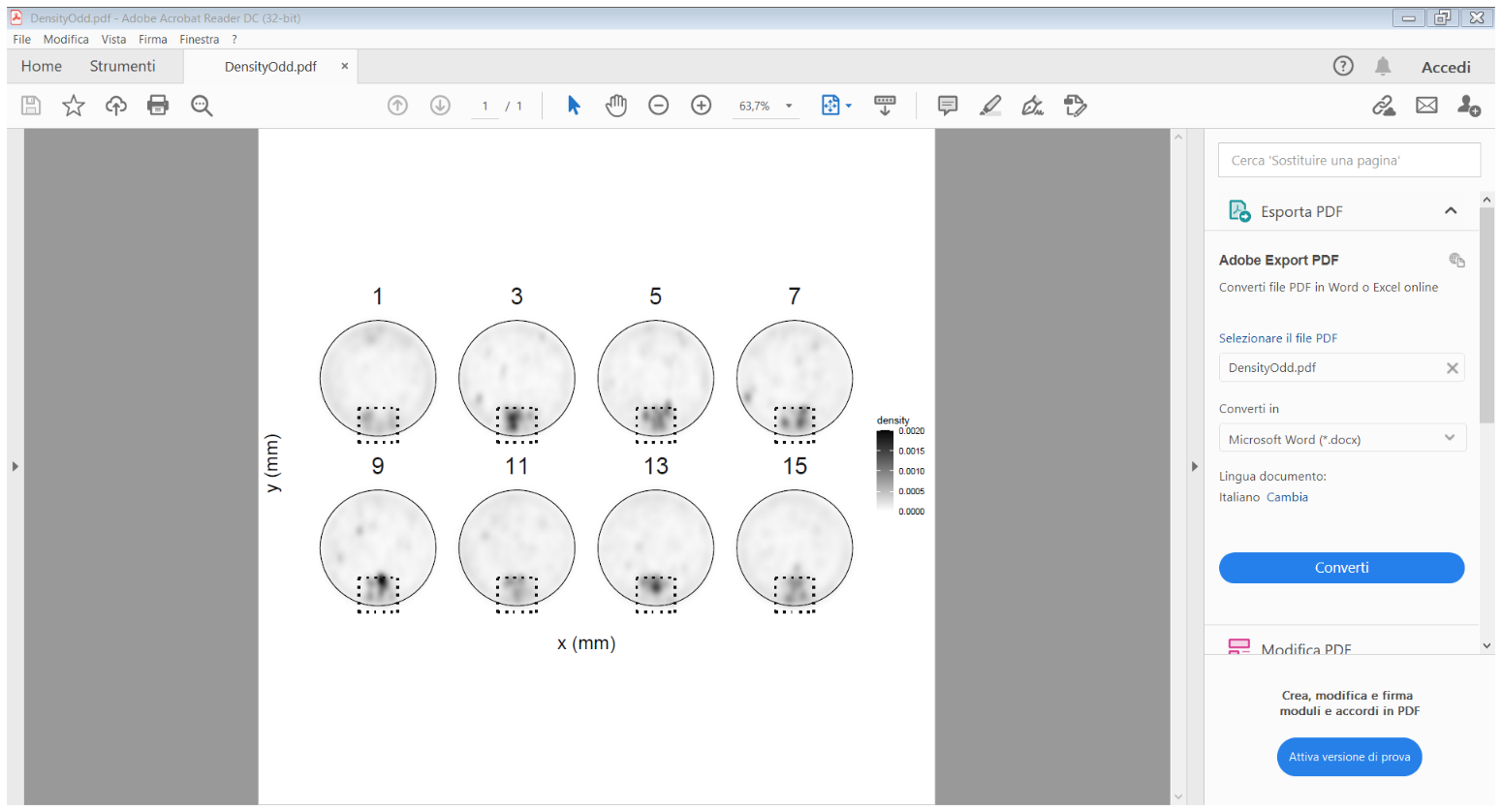

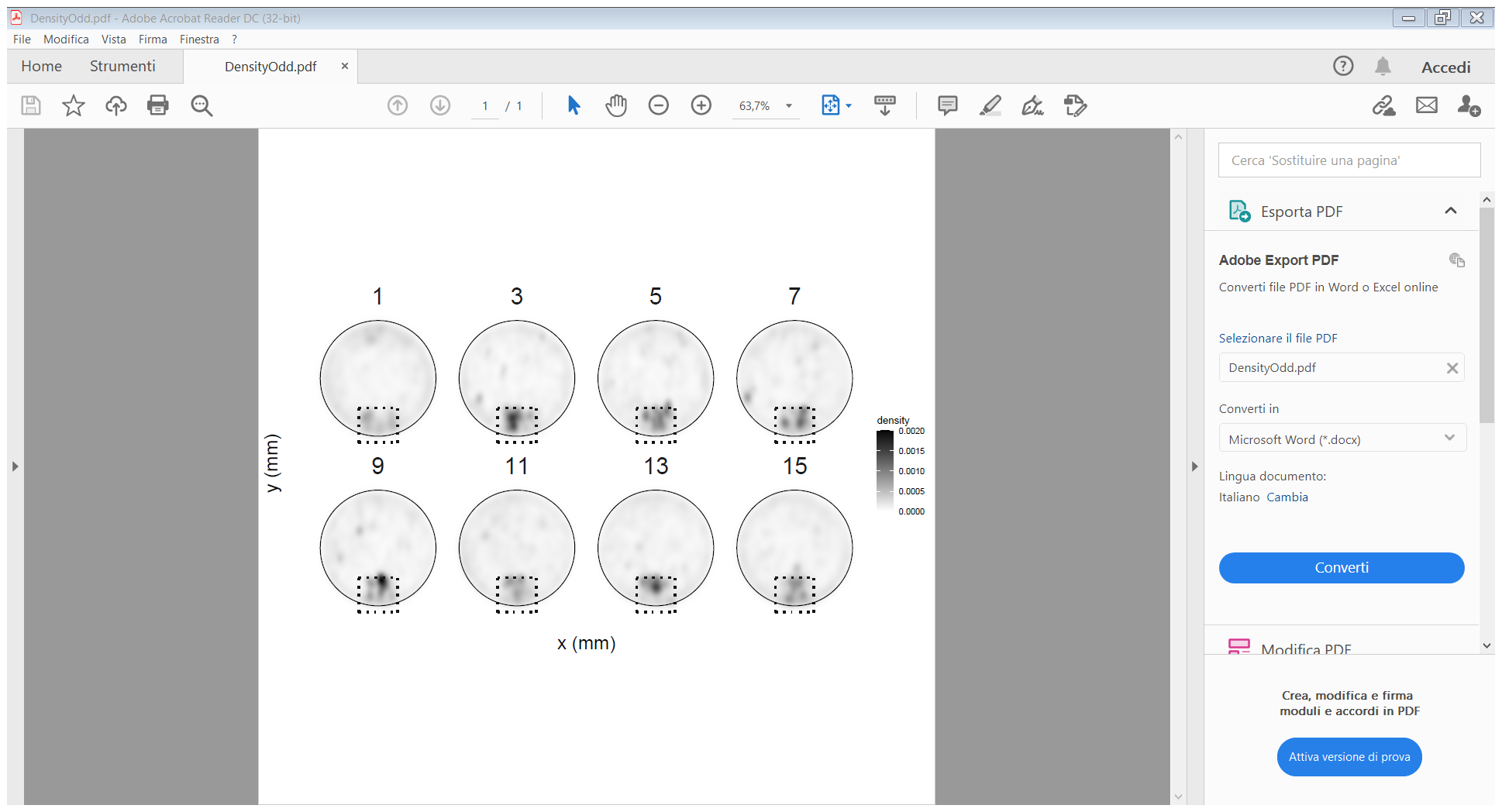

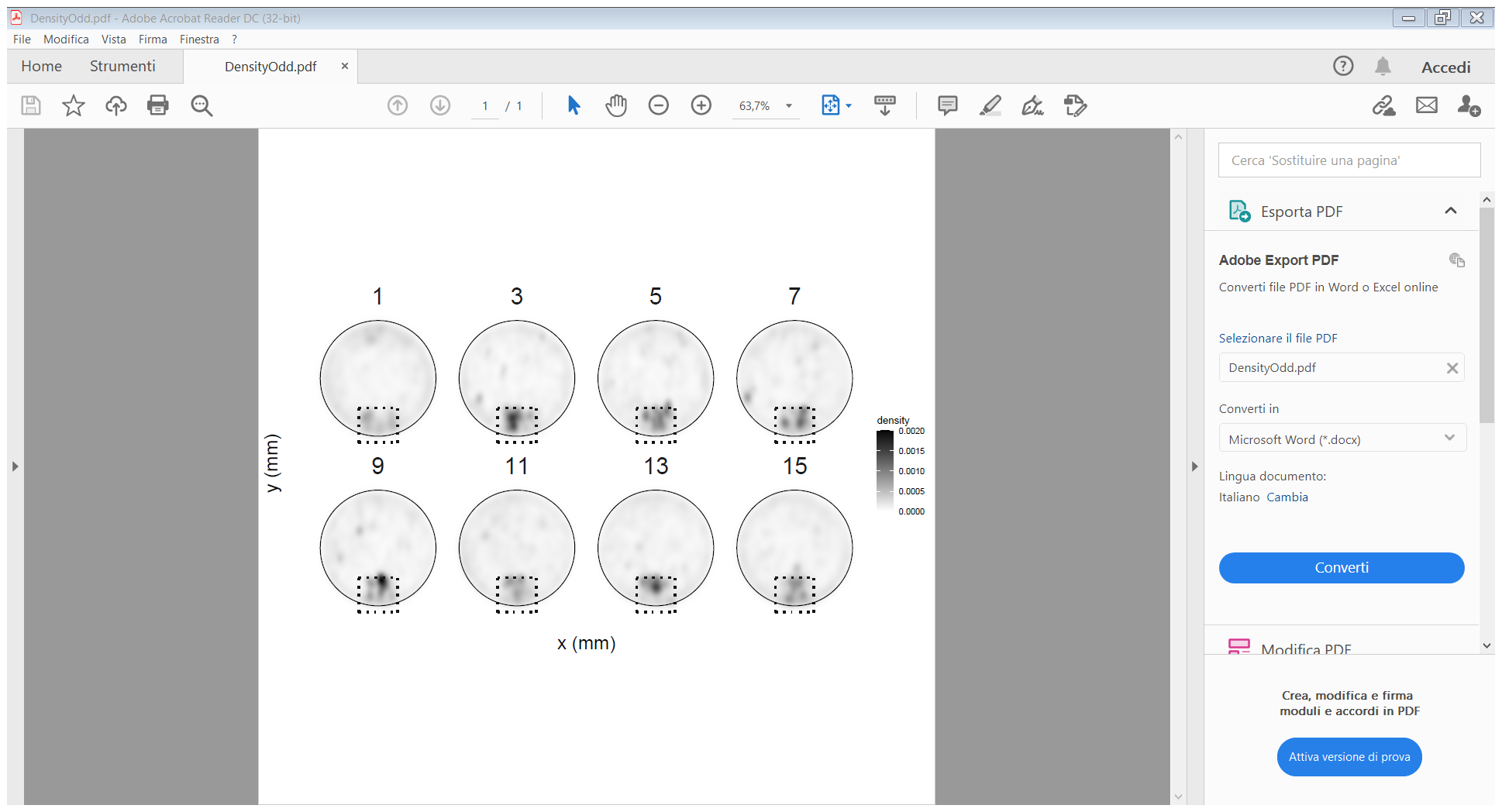

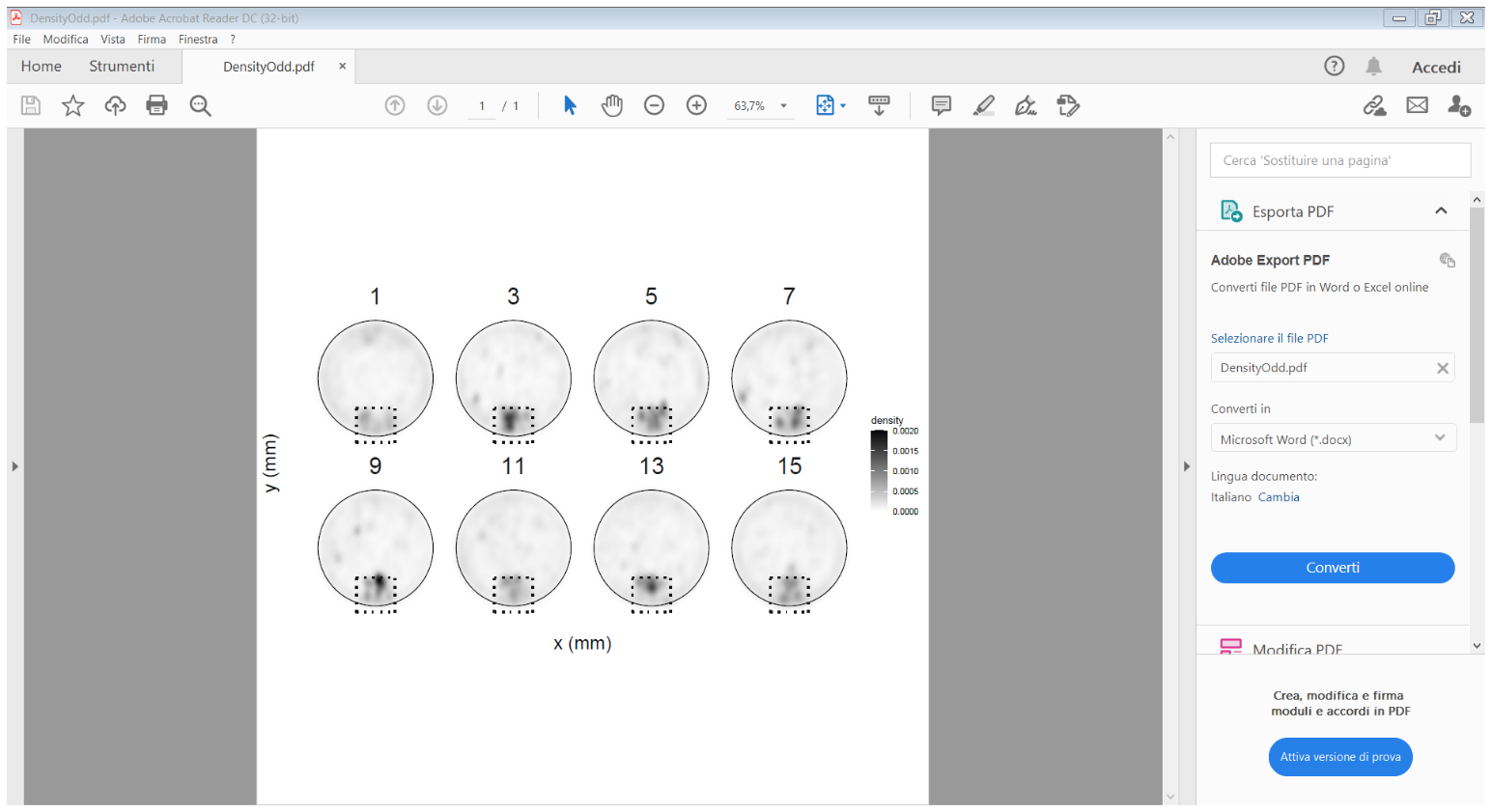

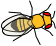

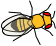

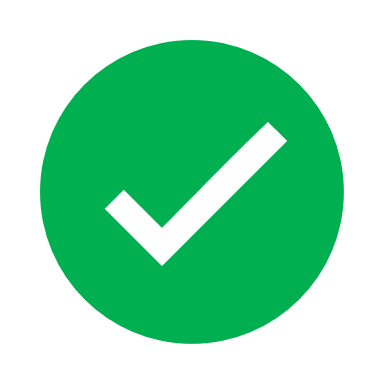

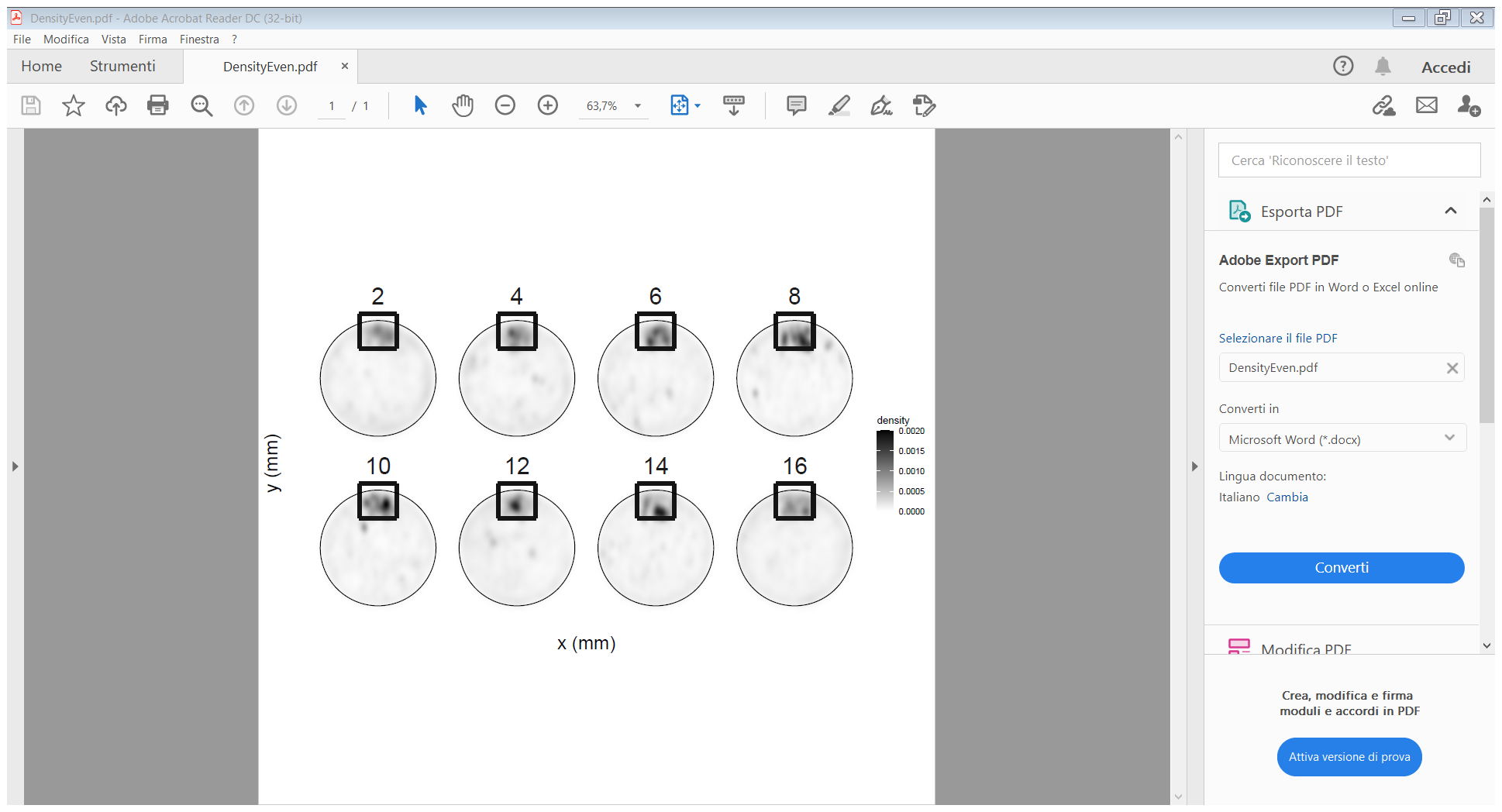

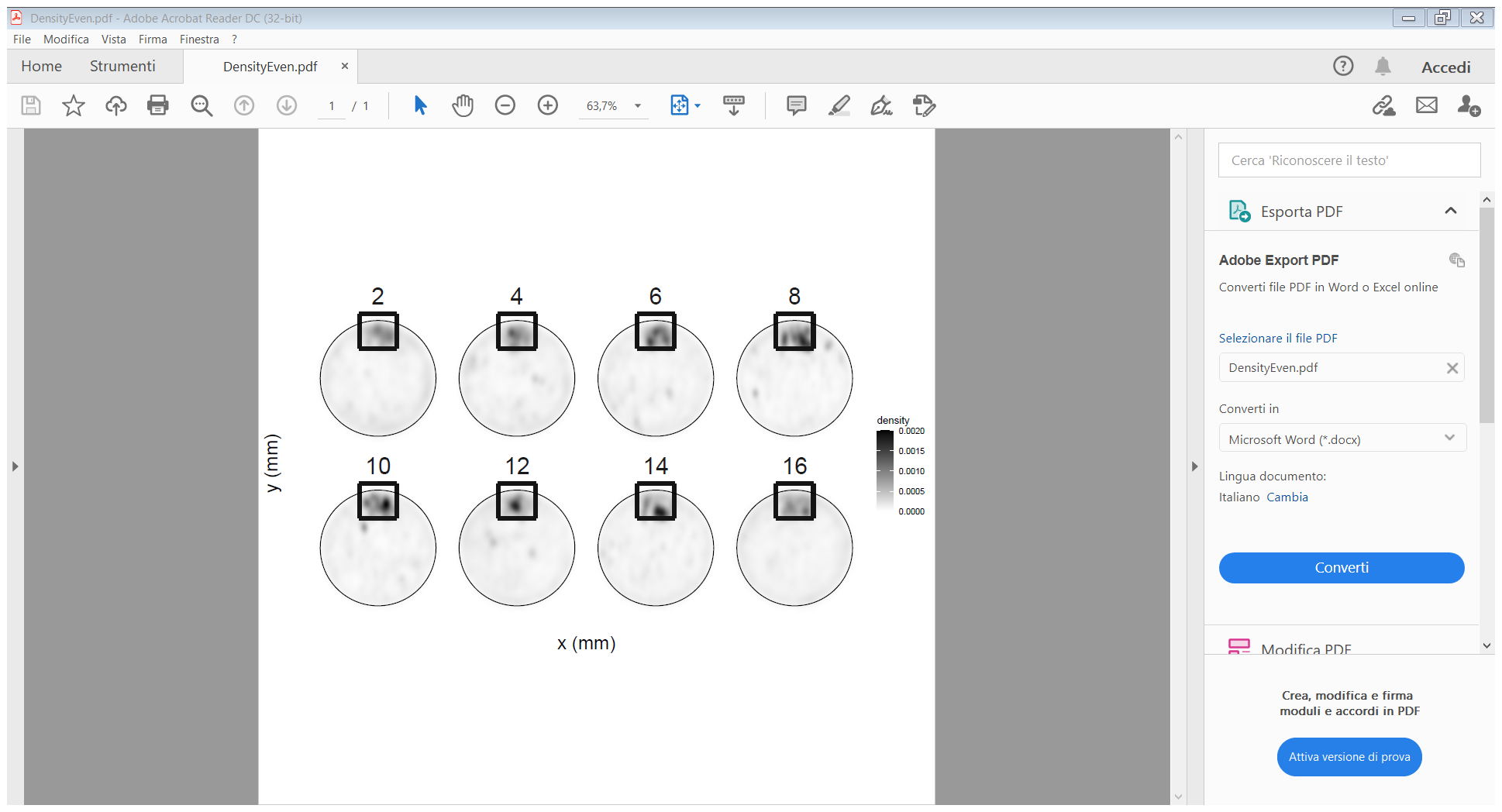

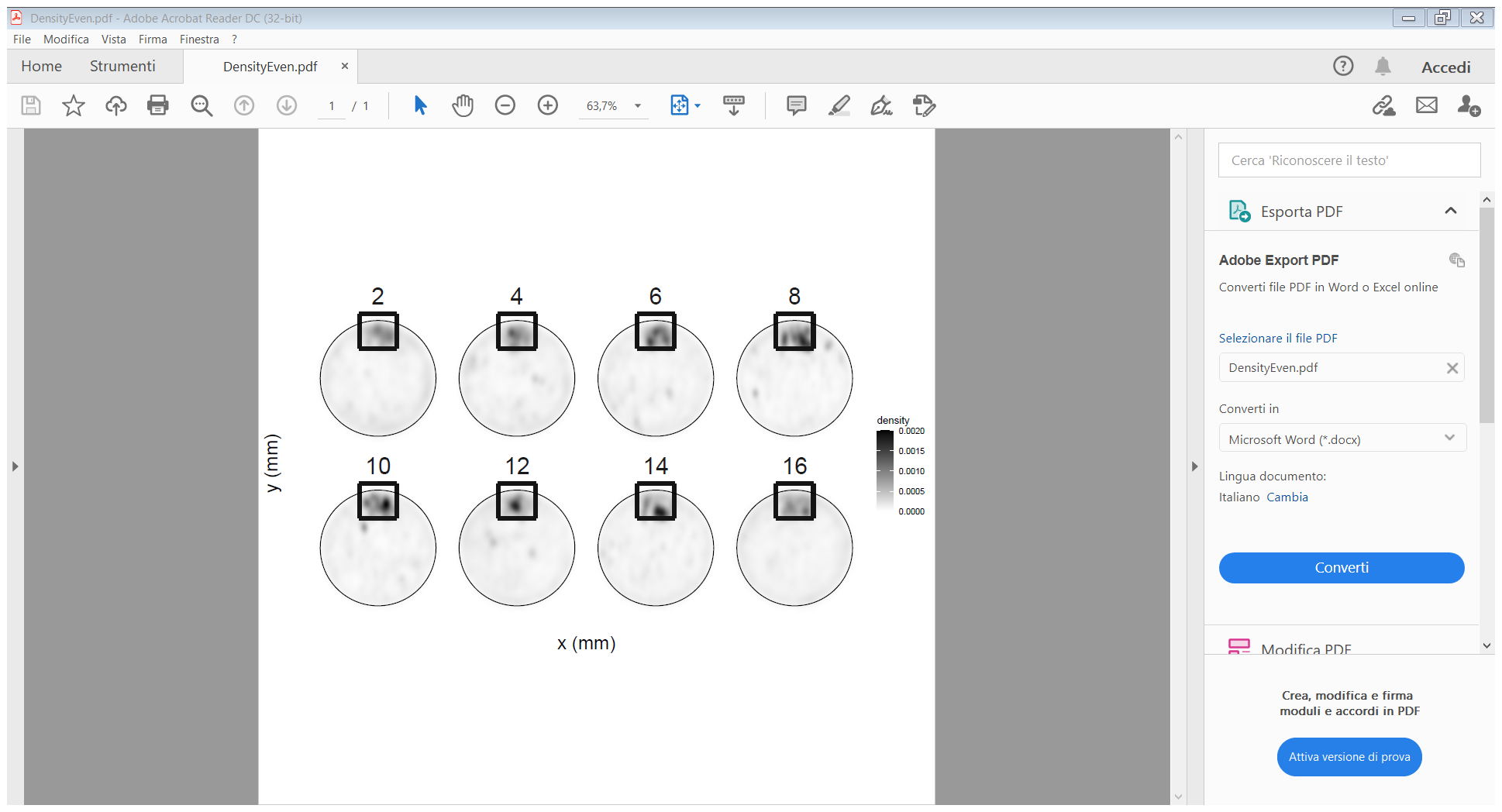

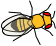

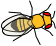
**

Trial 1-4

Trial 5-8

Trial 9-12

Trial 13-16

**A**

**B**

**C**

residency

**Replication Set**

**Supplementary Figure S4, part 1. Fruit flies training – replication set**

Black bars = represent the vertical or horizontal stripe; grey square = the safe zone; red bolt = the fly is being negatively stimulated; tick on green background = the fly is safe from the negative stimulation.

The behavioural paradigm is identical to the one described in the main text. In this set of experiments (no. flies = 41) we sought to replicate the findings related to the application of the Nearest Neighbour Rule reported in the main text. **A)** Performance Index (PI) as a function of training trials. PI = the difference between the time spent in the safe zone and the time spent in the previous (location of the) safe zone, divided by the total time spent in the two zones. The performance index can range from 1 to -1. A value of zero indicates no zone preference. PI was not computed for trial 1 given the absence of a Previous Safe Zone during that trial. Pointrange = mean ± confidence interval around the mean. Flies grow a statistically significant preference for the safe zone, marked by the vertical stripe, throughout training (see also Table S5); **B)** Velocity profile of fruit flies after entering a zone where relief is provided (black line) compared to the profile (grey line) after entering the non-safe zone (Table S6); **C)** density plots describing the residency of flies during training session, when bitter-taste stimulation is triggered if flies leave the safe zone (squared);

residency

residency

α

α

In/to zone marked by:

Bar approached:

Bar approached:

**D**

**E**

**F**

**G**

**H**

**I**

**J**

**K**

**L**

**M**

M(r)

M(r)

M(r)

M(r)

residency

α

Bar approached:

**P**

**N**

**O**

M(r)

residency

α

**S**

Bar approached:

M(r)

**Q**

**R**

**T**

**U**

**Supplementary Figure 4, part 2. Fruit flies resort to the Nearest Neighbour Rule soon after bitter stimulation onset – replication set**

Horizontal Green bar (e.g., panel D) = visual patterns displayed; horizontal orange bar = stimulation triggered according to fly position. Pointrange = mean ± confidence interval around the mean. **D)** In the 10 seconds after the onset of optogenetic stimulation, the difference between the number of flies that approached either one of the two zones is not significant (Table S6); **E)** There is no difference between the mean number of visits to the two zones in the periods considered, further implying that flies searched both zones in the same measure (Table S6); panels from F) to M) are relative to even-numbered trials. **F)** Spatial position of fruit flies, at second 60.1, (when the first pulse of optogenetic stimulation is delivered) that first entered the safe zone, marked by the vertical stripe **G)** Marcon and Puech’s M function value (black line) represents the distance between the observed flies positions compared to 10.000 random distribution simulations (red dashed line and grey shading). A value greater than 1 suggests aggregation. A goodness-of-fit test reveals that flies are significantly more aggregated than expected (p = 0.000 (meaning M(r) > 1), for n = 75 positions tested); **H)** fruit flies that entered the safe zone oriented themselves that zone after the onset of the bitter stimulation (no. orientations tested = 12026, mean orientation difference after-before the onset of stimulation, in degrees = 6.12, std error = 2.06, z.ratio = 2.97, p = 0.003); **I)** Spatial distribution at second 60.1 of fruit flies that will approach the previous safe zone; **J)** M function value is significantly greater than expected under the null hypothesis, suggesting aggregation of fly positions (p = 0.0014, for n = 92 positions); **K)** the fruit flies that entered the previous safe zone oriented themselves towards it; L**)** Spatial position of both groups of flies at second 60.1 (no. orientations tested = 12026, mean orientation difference = -46.16, std error = 1.89, z.ratio = -24.45, p < 0.0001); **M)** M function value assessing whether the two distributions of flies’ position reported in C), F) or I) consist of two distinct aggregates. The M function is < 1, thus suggesting spatial repulsion between the two groups of flies, indicating that flies entering the safe zone are spatially segregated from flies that entering the previous safe zone (p = 0.0011, no. positions tested = 167). From N) to U) are relative to odd-numbered trials. **N)** Spatial position of fruit flies, at second 60.1 (when the first pulse of optogenetic stimulation is delivered), that will first enter the safe zone marked by the vertical stripe; **O)** Marcon and Puech’s M function value (black line) represents the distance between the observed flies positions compared to 10.000 random distribution simulations (red dashed line and grey shading). A value greater than 1 suggests aggregation. A goodness-of-fit test reveals that flies are significantly more aggregated than expected (number of positions = 65, p = 0.000); **P)** fruit flies that entered the safe zone oriented themselves towards the safe zone after the onset of the bitter stimulation (i.e. they reduce significantly the angle difference between their orientation and the segment joining their position to the marker – no. orientations sampled = 9473, mean difference after – before stimulation (degrees) = -26.8, std error = 2.45 z.ratio = -10.929, p < 0.0001); **Q)** Spatial distribution of fruit flies, at second 60.1, that will approach the horizontal bar;

**R)** M function value is significantly greater than expected under the null hypothesis (number of observations = 73, p = 0.000); **S)** the fruit flies that entered the previous safe zone oriented themselves towards that zone (no. orientations = 9473, mean difference = 15.8, std error = 2.59, z.ratio = 6.102, p < 0.0001); **T)** Spatial position of both groups of flies at second 60.1; **U)** M function value assessing whether the two distributions of flies’ position reported in A), D) and G) actually consist of two distinct aggregates. The M function is < 1, thus suggesting spatial repulsion between the two groups of flies, indicating that flies that entered the safe zone were spatially segregated from flies that entered the previous safe zone (number of observations = 138, p = 0.000).

|  | **Performance index** |  | **Performance index** |
| --- | --- | --- | --- |
| *Predictors* | *Estimates, mean (CI 95%)* | *Predictors* | *Estimates, mean (CI 95%)* |
| (Intercept) | 0.29 ^***^ (0.14 – 0.44) | ntrial [10] | 0.28 ^**^ (0.07 – 0.50) |
| ntrial [3] | 0.13  (-0.08 – 0.34) | ntrial [11] | 0.04  (-0.17 – 0.25) |
| ntrial [4] | 0.05  (-0.16 – 0.27) | ntrial [12] | 0.24 ^*^ (0.03 – 0.45) |
| ntrial [5] | 0.03  (-0.18 – 0.24) | ntrial [13] | 0.19  (-0.02 – 0.41) |
| ntrial [6] | 0.21  (-0.00 – 0.42) | ntrial [14] | 0.23 ^*^ (0.02 – 0.44) |
| ntrial [7] | 0.04  (-0.17 – 0.25) | ntrial [15] | 0.18  (-0.03 – 0.39) |
| ntrial [8] | 0.29 ^**^ (0.08 – 0.50) | ntrial [16] | 0.23 ^*^ (0.02 – 0.44) |
| ntrial [9] | 0.21 ^*^ (0.00 – 0.43) |  |  |
| Flies tested = 41 | | | |
| ** p<0.05   ** p<0.01   *** p<0.001* | | | |

**Table S5 related to Supplementary Figure S4. Performance index for each trial.**

ntrial = trial number. CI 95% = 95% confidence interval. The estimates for the intercept and ntrial[3],…, ntrial[16] are the output of a linear mixed-effects model that describes the performance index as a function of trials, for each animal. PI was not computed for trial 1 given the absence of a Previous Safe Zone during that trial. The performance index during ntrial [2] is used as the reference value to compute the performance index estimates during the remaining ntrials.

| **SUPPL. FIGURE S4B. VELOCITY (LME)** | df | BIC |
| --- | --- | --- |
| Number of observed velocities: 46649, Flies n: 41 |  |  |
| Velocity ~ Frame_ins_Zone + (1\|Exp) | 4 | 235339 |
| Velocity ~ Frame_ins_Zone + Zone (1\|Exp) | 5 | 221629 |
| Velocity ~ Frame_ins_Zone*Zone + (1\|Exp) | 6 | **221024** |
|  |  |  |
| **SUPPL. FIGURE S4D. NUMBER OF FLIES (GLMM)** | df | BIC |
| Number of observed flies: 90, nTrial: 15 |  |  |
| No.Flies ~ 1 + (1\|nTrial) | 2 | 512.04 |
| No.Flies ~ Zone + (1\|nTrial) | 3 | 515.12 |
| No.Flies ~ nperiod + (1\|nTrial) | 4 | **426.48** |
| No.Flies ~ Zone + nperiod + (1\|nTrial) | 5 | 429.56 |
| No.Flies ~ Zone*nperiod + (1\|nTrial) | 7 | 438.51 |
|  |  |  |
| **SUPPL. FIGURE S4E. NUMBER OF VISITS (GLMM)** | df | BIC |
| Number of observed visits: 182, Flies n: 41 |  |  |
| No.Visits ~ 1 + (1\|Exp) | 2 | 786.52 |
| No.Visits ~ Zone + (1\|Exp) | 3 | 791.59 |
| No.Visits ~ nperiod + (1\|Exp) | 4 | **699.46** |
| No.Visits ~ Zone + nperiod + (1\|Exp) | 5 | 704.38 |
| No.Visits ~ Zone*nperiod + (1\|Exp) | 7 | 713.84 |
|  |  |  |
| **SUPPL. FIGURE S4H. Angle Difference Towards Northern target (LME)** | df | BIC |
| Number of observed angle differences: 12026, Flies n: 41, nTrial: 15 |  |  |
| Angle Diff ~ 1 + (1\|Exp/nTrial) | 4 | 137104 |
| Angle Diff ~ nperiod + (1\|Exp/nTrial) | 5 | 136822 |
| Angle Diff ~ nperiod + target + (1\|Exp/nTrial) | 6 | 136823 |
| Angle Diff ~ nperiod*target + (1\|Exp/nTrial) | 7 | **136440** |
|  |  |  |
| **SUPPL. FIGURE S4K. Angle Difference Towards Southern target (LME)** | df | BIC |
| Number of observed angle differences: 12026, Flies n: 41, nTrial: 15 |  |  |
| Angle Diff ~ 1 + (1\|Exp/nTrial) | 4 | 137102 |
| Angle Diff ~ nperiod + (1\|Exp/nTrial) | 5 | 136864 |
| Angle Diff ~ nperiod + target + (1\|Exp/nTrial) | 6 | 136864 |
| Angle Diff ~ nperiod*target + (1\|Exp/nTrial) | 7 | **136528** |
|  |  |  |
| **Zone entered (binomial: Yes/No) as a function of position and orientation at second 60.1 – during EVEN-numbered trials** | df | BIC |
| Number of observed positions/orientations: 136, Flies n: 41, nTrial: 15 |  |  |
| Zone South ~ 1 + (1\|Exp/nTrial) | 3 | 202.54 |
| Zone South ~ Distance + (1\|Exp/nTrial) | 4 | **186.59** |
| Zone South ~ Distance + Angle Diff + (1\|Exp/nTrial) | 5 | 190.65 |
| Zone South ~ Distance*Angle Diff + (1\|Exp/nTrial) | 6 | 194.02 |
|  |  |  |
| Zone North ~1 + (1\|Exp/nTrial) | 3 | 202.54 |
| Zone North ~ Distance + (1\|Exp/nTrial) | 4 | **182.20** |
| Zone North ~ Distance + Angle Diff + (1\|Exp/nTrial) | 5 | 185.82 |
| Zone North ~ Distance*Angle Diff + (1\|Exp/nTrial) | 6 | 190.07 |
|  |  |  |
| **SUPPL. FIGURE S4P. Angle Difference Towards Southern target (LME)** | df | BIC |
| Number of observed angle differences: 9473, Flies n: 39 |  |  |
| Angle Diff ~ 1 + (1\|Exp/nTrial) | 4 | 110472 |
| Angle Diff ~ nperiod + (1\|Exp/nTrial) | 5 | 110470 |
| Angle Diff ~ nperiod + target + (1\|Exp/nTrial) | 6 | 110469 |
| Angle Diff ~ nperiod*target + (1\|Exp/nTrial) | 7 | **110323** |
|  |  |  |
| **SUPPL. FIGURE 4S. Angle Difference Towards Northern target (LME)** | df | BIC |
| Number of observed angle differences: 9473, Flies n: 39 |  |  |
| Angle Diff ~ 1 + (1\|Exp/nTrial) | 4 | 110099 |
| Angle Diff ~ nperiod + (1\|Exp/nTrial) | 5 | 110107 |
| Angle Diff ~ nperiod + target + (1\|Exp/nTrial) | 6 | 110093 |
| Angle Diff ~ nperiod*target + (1\|Exp/nTrial) | 7 | **110019** |
|  |  |  |
| **Zone entered (binomial: Yes/No) as a function of position and orientation at second 60.1 – during ODD-numbered trials** | df | BIC |
| Number of observed positions/orientations: 136, Flies n: 41 |  |  |
| Zone South ~ 1 + (1\|Exp/nTrial) | 3 | 159.28 |
| Zone South ~ Distance + (1\|Exp/nTrial) | 4 | **132.49** |
| Zone South ~ Distance + Angle Diff + (1\|Exp/nTrial) | 5 | 136.38 |
| Zone South ~ Distance*Angle Diff + (1\|Exp/nTrial) | 6 | 140.22 |
|  |  |  |
| Zone North ~1 + (1\|Exp/nTrial) | 3 | 159.28 |
| Zone North ~ Distance + (1\|Exp/nTrial) | 4 | 135.73 |
| Zone North ~ Distance + Angle Diff + (1\|Exp/nTrial) | 5 | **135.48** |
| Zone North ~ Distance*Angle Diff + (1\|Exp/nTrial) | 6 | 139.92 |
|  |  |  |

**Table S6. Models details related to the replication set described in Suppl. Figure 4.**

LME = Linear Mixed-Effects (model); GLMM = Generalised Linear Mixed Model (in these models, family = poisson); BIC = Bayesian Information Criterion; (1|x) = variable “x” is considered a random effect; (1|x/y) = nested random effect of variable *y* for each *x*; Exp = experiment (single fly); Frame_ins_Zone = frames since the entrance of the fly into a specific zone; nTrial = trial number; nperiod = period number (each period consists of 10 seconds). The “*” sign = interaction between the variables. The variable before the tilde (~) is the outcome to be estimated by the regression model(s). The explanatory variables are placed after the tilde. The models are displayed in increasing order of degrees of freedom (df). BIC was taken into consideration for the selection of the best model. The best model for each figure is the one with the lowest BIC (in bold). If, for example, the best model for Suppl. Figure S4D (No.Flies ~ nperiod + (1|nTrial)) has a lower BIC than the model considering also zone (No.Flies ~ Zone + nperiod + (1|nTrial)), it means that the role of zone in determining the number of flies is not significant (i.e., the number of flies in the different zones is not significantly different).

Regarding the binomial models to predict which zone will be entered based on fly position and orientations at second 60.1, we also tested an additional set of models, but this time considering the mean orientations in the four seconds before the stimulation onset (and not only the orientation at second 60.1, in order to account for fly heading direction). Nevertheless, the fly mean orientation does not improve a model that already accounted for the fly position in the arena (regarding the Zone North, model “m1” accounting for distance to the zone BIC = 186.59, model “m1 + mean orientation” BIC = 190.54, model of interaction between distance and orientation BIC = 194.89). A similar conclusion is also reached for Zone South and for the odd-numbered trials.

Before opting for including training progression (“nTrial”) as random intercept in the models related to Figure 2A and 2B, we ascertained that the effect of this variable (transformed to continuous) on the studied outcomes was negligible (i.e., that there was no trend towards an increase or reduction in the number of visits/flies throughout training) – see next page.

| Model | df | BIC |
| --- | --- | --- |
| No. Visits ~ Zone + (1\|Exp) | 3 | **2735.5** |
| No. Visits ~ Zone + nTrial + (1\|Exp) | 4 | 2742.5 |
| No. Visits ~ Zone*nTrial + (1\|Exp) | 5 | 2749.3 |
|  |  |  |
| No. Flies ~ Zone + (1\|Exp) | 3 | 501.98 |
| No. Flies ~ Zone + nTrial + (1\|Exp) | 4 | **498.78*** |
| No. Flies ~ Zone*nTrial + (1\|Exp) | 5 | 503.06 |

*****in this specific case, the effect size of “ntrial” was negligible when compared to the constant value (i.e., intercept). In fact, the number of flies to the two zones was reduced by 2% for each trial (β = -0.026, std. error = 0.009) with an intercept of β = 1.98, std. error = 0.16.

residency

**A**

In/to zone that is:

M(r)

M(r)

α

**H**

Zone approached:

**B**

**C**

**D**

**E**

**F**

**G**

**L**

**M**

residency

M(r)

α

**K**

Zone approached:

**I**

**J**

*Legend on next page*

**Supplementary Figure S5, part 1. The nearest neighbour rule is applied even in an ambiguous visual environment.**

Black bars = represent the vertical or horizontal stripe; red bolt = the fly is being negatively stimulated;

Green bar = visual patterns displayed; orange bar = stimulation triggered according to fly position. Pointrange = mean ± confidence interval around the mean. **A)** Data presented in this figure are related to a subgroup of flies (n = 40) that were trained in an ambiguous visual environment. The animals were presented two, diametrically-opposed vertical bars, and the safe zone was moved alternately between the two **B)** Performance Index (PI) as a function of training trials. The 0 value marks indifference in the time spent in the safe or previous safe zone. PI was not computed for trial 1 given the absence of a Previous Safe Zone during that trial. Flies showed a stable preference for the safe zone throughout training, but such preference did not significantly increase throughout the training (see also Table S7); **C)** During the probe session, fruit flies spent an equal amount of time close to the vertical bar at the northern-end of the arena as well as to the bar at the southern end (no. of comparisons = 79, mean difference in time spent (s) = -3.04, std error = 1.81, t.ratio = -1,677, p = 0.097), this supports the concept that this group of flies did not learn the sequential change of position of the safe zone (at north during even-numbered trials, at south during odd-numbered trials). Otherwise, the flies should have shown a preference, or tended to prefer, the vertical bar at the southern end of the arena during the probe session (i.e., where the safe zone could be found during the probe/trial no. 17). **D)** In the 10 seconds after the onset of optogenetic stimulation, the difference between the number of flies that approached either one of the two zones is not significant (see also Tables S8); **E)** There is no difference between the mean number of visits to the two zones in the periods considered, further implying that flies searched both zones in the same measure (see also Tables S8). **F)** Spatial position of fruit flies, at second 60.1 (when the first pulse of optogenetic stimulation is delivered), that will first enter the safe zone; **G)** Marcon and Puech’s M function value (black line) represents the distance between the observed flies positions compared to 10.000 random distribution simulations (red dashed line and grey shading). A value greater than 1 suggests aggregation. A goodness-of-fit test reveals that flies are significantly more aggregated than expected (number of observations = 76, p < 0.001); **H)** fruit flies that entered the safe zone oriented themselves towards the safe zone after the onset of the bitter stimulation (no. orientations = 10800, mean difference after – before stimulation (degrees) = 23, std error = 2.29, z.ratio = 10.046, p <0.0001). **I)** Spatial distribution of fruit flies, at second 60.1, that will approach the previous safe zone; **J)** M function value is significantly greater than expected under the null hypothesis (number of observations = 76, p < 0.001); **K)** the fruit flies that entered the previous safe zone oriented themselves towards the previous safe zone (no. orientations = 10800, mean difference = - 56.6, std error = 2.36, z.ratio = -23.94, p <0.0001); **L)** Spatial position of both groups of flies at second 60.1; **M)** M function value assessing whether the two distributions of flies’ position reported in F), I) or L) actually consist of two distinct aggregates. The M function is < 1, thus suggesting spatial repulsion between the two groups of flies, indicating that flies that entered the safe zone were spatially segregated from flies that entered the previous safe zone (number of observations = 152, p < 0.001).

*Continues on next page*

residency

M(r)

M(r)

Zone approached:

α

**P**

**N**

**O**

**T**

**U**

residency

M(r)

α

Zone approached:

**S**

**Q**

**R**

**Supplementary Figure 5, part 2. The nearest neighbour rule is applied even in an ambiguous visual environment.**

**N)** Spatial position of fruit flies, at second 60.1, that will first enter the safe zone; **O)** Marcon and Puech’s M function value (black line) represents the distance between the observed flies positions compared to 10.000 random distribution simulations (red dashed line and grey shading). Flies are significantly more aggregated than expected (number of observations = 63, p = 0.01); **P)** the fruit flies that entered the safe zone oriented themselves towards the safe zone after the onset of the bitter stimulation (no. orientations = 8743, mean difference = -32.3, std. error = 2.62, z.ratio = -12.357, p < 0.0001). **Q)** Spatial distribution, at second 60.1, of fruit flies that will approach the previous safe zone; **R)** M function value is significantly greater than expected under the null hypothesis (number of observations = 74, p = 0.007); **S)** the fruit flies that entered the previous safe zone oriented themselves towards the previous safe zone (no. orientations = 8743, mean difference = 44.3, std. error = 2.38, z.ratio = 18.593, p < 0.0001 ); **T)** Spatial position of both groups of flies at second 60.1; **U)** M function value assessing whether the two distributions of flies’ position reported in N), Q) or T) actually consist of two distinct aggregates. The M function is < 1, thus suggesting spatial repulsion between the two groups of flies, indicating that flies that entered the current safe zone were spatially segregated from flies that entered the previous safe zone (number of observations = 137, p = 0.009).

|  | **Performance index** |  | **Performance index** |
| --- | --- | --- | --- |
| *Predictors* | *Estimates, mean (CI 95%)* | *Predictors* | *Estimates, mean (CI 95%)* |
| (Intercept) | 0.41 ^***^ (0.26 – 0.57) | ntrial [10] | 0.08  (-0.13 – 0.29) |
| ntrial [3] | 0.02  (-0.19 – 0.23) | ntrial [11] | 0.10  (-0.11 – 0.31) |
| ntrial [4] | 0.07  (-0.14 – 0.28) | ntrial [12] | 0.04  (-0.17 – 0.25) |
| ntrial [5] | 0.07  (-0.14 – 0.28) | ntrial [13] | 0.04  (-0.17 – 0.25) |
| ntrial [6] | 0.06  (-0.15 – 0.27) | ntrial [14] | 0.08  (-0.13 – 0.29) |
| ntrial [7] | 0.07  (-0.14 – 0.28) | ntrial [15] | 0.09  (-0.12 – 0.30) |
| ntrial [8] | 0.13  (-0.08 – 0.34) | ntrial [16] | -0.01  (-0.22 – 0.20) |
| ntrial [9] | -0.03  (-0.25 – 0.18) |  |  |
| Flies tested = 40 | | | |
| ** p<0.05   ** p<0.01   *** p<0.001* | | | |

**Table S7 related to Figure S5B. Performance Index of flies trained in an ambiguous visual environment.**

ntrial = trial number. CI 95% = 95% confidence interval. The estimates for the intercept and ntrial[3],…, ntrial[16] are the output of a linear mixed-effects model that describes the performance index as a function of trials, for each animal. PI was not computed for trial 1 given the absence of a Previous Safe Zone during that trial. The performance index during ntrial [2] is used as the reference value to compute the performance index estimates during the remaining ntrials.

| **SUPPL. FIGURE S5D. NUMBER OF FLIES (GLMM)** | df | BIC |
| --- | --- | --- |
| Number of observed flies: 90, nTrials: 15 |  |  |
| No.Flies ~ 1 + (1\|nTrial) | 2 | 497.95 |
| No.Flies ~ Zone + (1\|nTrial) | 3 | 496.36 |
| No.Flies ~ nperiod + (1\|nTrial) | 4 | 435.46 |
| No.Flies ~ Zone + nperiod + (1\|nTrial) | 5 | **433.87** |
| No.Flies ~ Zone*nperiod + (1\|nTrial) | 7 | 496.36 |
|  |  |  |
| **SUPPL. FIGURE S5E. NUMBER OF VISITS (GLMM)** | df | BIC |
| Number of observed visits: 176, Flies n: 40 |  |  |
| No.Visits ~ 1 + (1\|Exp) | 2 | 783.02 |
| No.Visits ~ Zone + (1\|Exp) | 3 | 788.08 |
| No.Visits ~ nperiod + (1\|Exp) | 4 | **710.24** |
| No.Visits ~ Zone + nperiod + (1\|Exp) | 5 | 715.27 |
| No.Visits ~ Zone*nperiod + (1\|Exp) | 7 | 724.13 |
|  |  |  |
| **SUPPL. FIGURE S5H. Angle Difference Towards Northern target – EVEN-numbered trials (LME)** | df | BIC |
| Number of observed angle differences: 10800, Flies n: 40 |  |  |
| Angle Diff ~ 1 + (1\|Exp/nTrial) | 4 | 125650 |
| Angle Diff ~ nperiod + (1\|Exp/nTrial) | 5 | 125615 |
| Angle Diff ~ nperiod + target + (1\|Exp/nTrial) | 6 | 125606 |
| Angle Diff ~ nperiod*target + (1\|Exp/nTrial) | 7 | **125168** |
|  |  |  |
| **SUPPL. FIGURE S5K. Angle Difference Towards Southern target – EVEN-numbered trials (LME)** | df | BIC |
| Number of observed angle differences: 10800, Flies n: 40 |  |  |
| Angle Diff ~ 1 + (1\|Exp/nTrial) | 4 | 126030 |
| Angle Diff ~ nperiod + (1\|Exp/nTrial) | 5 | 125959 |
| Angle Diff ~ nperiod + target + (1\|Exp/nTrial) | 6 | 125954 |
| Angle Diff ~ nperiod*target + (1\|Exp/nTrial) | 7 | **125381** |
|  |  |  |
| **SUPPL. FIGURE S5P. Angle Difference Towards Southern target – ODD-numbered trials (LME)** | df | BIC |
| Number of observed angle differences: 8743, Flies n: 38 |  |  |
| Angle Diff ~ 1 + (1\|Exp/nTrial) | 4 | 101571 |
| Angle Diff ~ nperiod + (1\|Exp/nTrial) | 5 | 101529 |
| Angle Diff ~ nperiod + target + (1\|Exp/nTrial) | 6 | 101530 |
| Angle Diff ~ nperiod*target + (1\|Exp/nTrial) | 7 | **101013** |
|  |  |  |
| **SUPPL. FIGURE S5S. Angle Difference Towards Northern target – ODD-numbered trials (LME)** | df | BIC |
| Number of observed angle differences: 8743, Flies n: 38 |  |  |
| Angle Diff ~ 1 + (1\|Exp/nTrial) | 4 | 101663 |
| Angle Diff ~ nperiod + (1\|Exp/nTrial) | 5 | 101639 |
| Angle Diff ~ nperiod + target + (1\|Exp/nTrial) | 6 | 101642 |
| Angle Diff ~ nperiod*target + (1\|Exp/nTrial) | 7 | **101209** |
|  |  |  |
| **Zone entered (binomial: Yes/No) as a function of position and orientation at second 60.1 – during EVEN-numbered trials** | df | BIC |
| Number of observed positions/orientations: 119, Flies n: 40 |  |  |
| Zone South ~ 1 + (1\|Exp/nTrial) | 3 | 179.23 |
| Zone South ~ Distance + (1\|Exp/nTrial) | 4 | **163.96** |
| Zone South ~ Distance + Angle Diff + (1\|Exp/nTrial) | 5 | 168.63 |
| Zone South ~ Distance*Angle Diff + (1\|Exp/nTrial) | 6 | 173.36 |
|  |  |  |
| Zone North ~1 + (1\|Exp/nTrial) | 3 | 179.23 |
| Zone North ~ Distance + (1\|Exp/nTrial) | 4 | **165.37** |
| Zone North ~ Distance + Angle Diff + (1\|Exp/nTrial) | 5 | 168.09 |
| Zone North ~ Distance*Angle Diff + (1\|Exp/nTrial) | 6 | 172.81 |
|  |  |  |
| **Zone entered (binomial: Yes/No) as a function of position and orientation at second 60.1 – during ODD-numbered trials** | df | BIC |
| Number of obs: 95, Flies n: 37 |  |  |
| Zone South ~ 1 + (1\|Exp/nTrial) | 3 | 143.30 |
| Zone South ~ Distance + (1\|Exp/nTrial) | 4 | **133.39** |
| Zone South ~ Distance + Angle Diff + (1\|Exp/nTrial) | 5 | 137.94 |
| Zone South ~ Distance*Angle Diff + (1\|Exp/nTrial) | 6 | 142.48 |
|  |  |  |
| Zone North ~1 + (1\|Exp/nTrial) | 3 | 143.30 |
| Zone North ~ Distance + (1\|Exp/nTrial) | 4 | **134.02** |
| Zone North ~ Distance + Angle Diff + (1\|Exp/nTrial) | 5 | 138.57 |
| Zone North ~ Distance*Angle Diff + (1\|Exp/nTrial) | 6 | 142.74 |

**Table S8. Models details related to Suppl. Figure S5.**

LME = Linear Mixed-Effects (model); GLMM = Generalised Linear Mixed Model (in these models, family = poisson); BIC = Bayesian Information Criterion; (1|x) = variable “x” is considered a random effect; (1|x/y) = nested random effect of variable *y* for each *x*; Exp = experiment (single fly); nTrial = trial number; nperiod = period number (each period consists of 10 seconds). The “*” sign = interaction between the variables. The variable before the tilde (~) is the outcome to be estimated by the regression model(s). The explanatory variables are placed after the tilde. The models are displayed in increasing order of degrees of freedom (df). BIC was taken into consideration for the selection of the best model. The best model for each figure is the one with the lowest BIC (in bold). If, for example, the best model for Figure 3E (No.Flies ~ nperiod + (1|nTrial)) has a lower BIC than the model also considering zone (No.Flies ~ Zone + nperiod + (1|nTrial)), it means that the role of zone in determining the number of flies is not significant (i.e., the number of flies in the different zones is not significantly different)
